# A Cluster Randomized Trial of a Vaccination Communication Educational Intervention: Impact on COVID-19 Vaccine Uptake in Veterans

**DOI:** 10.1007/s11606-026-10209-9

**Published:** 2026-02-12

**Authors:** Karen H. Seal, Adam Kaplan, Jennifer K. Manuel, Daniel Bertenthal, Natalie Purcell, Beth M. DeRonne, Karen Anderson Oliver, Denise Esserman, Nicole McCamish, Marie Mesidor, Brandon Griffin, Brian Borsari, Nicole A. Woodruff, Hajra Usman, Jeffrey M. Pyne

**Affiliations:** 1 San Francisco VA Health Care System, San Francisco, CA USA; 2 Department of Medicine, University of California, San Francisco, CA USA; 3 Department of Psychiatry and Behavioral Sciences, University of California, San Francisco, CA USA; 4 Center for Care Delivery and Outcomes Research, Minneapolis Veterans Affairs Health Care System, Minneapolis, MN USA; 5 Departments of Social and Behavioral Sciences and Nursing, University of California, San Francisco, CA USA; 6 Center for Mental Healthcare and Outcomes Research, Central Arkansas Veterans Healthcare System, Little Rock, AR USA; 7 Department of Biostatistics, Yale School of Public Health, New Haven, CT USA; 8 Northern California Institute for Research and Education, San Francisco, CA USA; 9 Central Arkansas Veterans Healthcare System, Little Rock, AR USA; 10 Department of Psychiatry, University of Arkansas for Medical Sciences, Little Rock, AR USA

**Keywords:** COVID-19, vaccination, veterans, clinical trial, motivational interviewing

## Abstract

**Background:**

Despite its safety and effectiveness, COVID-19 vaccine uptake declined later in the pandemic.

**Objective:**

To evaluate the effectiveness of a Motivational Interviewing (MI)-informed educational intervention for healthcare providers and staff on vaccination communication. We hypothesized that educating providers and staff about non-judgmental, collaborative discussions would enhance vaccine uptake in veterans.

**Design:**

A multi-site cluster randomized controlled implementation-effectiveness trial conducted at ten separate Veterans Health Administration (VA) facilities from February 2022 to May 2023.

**Participants:**

Veteran patients with a clinical visit at an enrolled VA facility within a year of the start of the trial and at least one primary care visit during 1-year follow-up.

**Interventions:**

VA facilities were randomized to either a Vaccine Communication Educational Intervention (VCI), which included provider and staff MI training and implementation facilitation, or usual care (UC) vaccination promotional activities.

**Main Measures:**

Primary outcome was receipt of any COVID-19 vaccine, and secondary outcome was completion of the COVID-19 vaccine primary series; receipt of COVID-19 booster and influenza (flu) vaccinations were exploratory outcomes.

**Key Results:**

Among 338,718 veterans, there was no significant difference between facilities randomized to VCI and UC regarding receipt of any COVID-19 vaccine (Adjusted Odds Ratio = 1.16, 95% Confidence Interval (0.89, 1.50), *p* = 0.28). There were also no significant differences in COVID-19 primary series and booster completion, or flu vaccine uptake. Significant predictors of vaccination among veterans included age, non-White race, Hispanic ethnicity, and greater primary care utilization.

**Conclusions:**

MI-informed vaccination communication education of VA providers and staff did not significantly improve uptake of COVID-19 and flu vaccination in veterans. Targeted outreach to sub-populations (e.g., younger veterans) and increased primary care utilization may enhance vaccination uptake. Lessons learned from this trial, including barriers to implementation, may inform future vaccine communication interventions given increasing vaccine hesitancy amid current outbreaks and the threat of future pandemics.

**Trial Registration:**

ClinicalTrials.gov Identifier NCT05027464.

**Supplementary Information:**

The online version contains supplementary material available at 10.1007/s11606-026-10209-9.

The COVID-19 pandemic resulted in significant global morbidity and mortality, with the USA recording approximately 1,065,200 excess deaths by January 2022.^1^ Following emergency use authorization in December 2020, vaccination was recommended and prevented severe illness and death.^2^ Veterans with chronic health conditions were a focus of vaccination efforts by the Veterans Health Administration (VA), where nearly 70% of patients completed the initial primary COVID-19 vaccine series by mid-2022.^3^ However, vaccination rates within the VA and across the USA declined in subsequent years due to a rise in vaccine hesitancy fueled by perceived safety concerns, misinformation, and sociopolitical influences.^4–6^

Healthcare providers play a crucial role in addressing vaccine hesitancy.^7–9^ However, time pressure, patient relationships, and personal beliefs about vaccination often dissuade them from engaging in vaccine-related discussions.^10,11^ VA providers are trained in Motivational Interviewing (MI), an evidence-based communication strategy, emphasizing empathy, patient autonomy, and collaborative decision-making, and MI has been used effectively for various behavioral targets (e.g., smoking cessation).^12–16^ While two trials of MI clinician training showed positive effects on vaccine uptake in the pediatric population ^16,17^, in the adult population, there has been only one trial of an MI-styled chatbot that increased vaccine readiness ^18^, and an ongoing trial of MI education for mental health providers to encourage vaccine uptake ^19^; all other publications have involved evaluations of MI clinician trainings in vaccine communication; hence the need for additional trials.^20^

The COVID-19 Vaccine Acceptance Study (CoVAcS) tested an MI-informed communication educational intervention for VA healthcare providers to encourage discussions about COVID-19 vaccination during later stages of the COVID-19 pandemic. The study tested the hypothesis that vaccine uptake would be greater among patients receiving care in VA facilities implementing provider vaccination communication training compared to facilities relying on usual vaccination efforts alone.^21^

## METHODS

### Trial Design Overview

Trial design and implementation strategy have been described previously (Supplement: Trial Protocol).^21^ CoVAcS was a pragmatic hybrid type 1 implementation-effectiveness cluster randomized controlled trial (CRCT) conducted during the COVID-19 pandemic from February 2022 to May 2023. Ten VA healthcare facilities were enrolled and randomized into two groups for 1 year: Vaccine Communication Educational Intervention (VCI), an MI-based training for healthcare providers focused on vaccine communication or usual care (UC), i.e., standard VA-wide COVID-19 vaccination activities.^21^ The research team utilized Implementation Facilitation as the implementation strategy,^22,23^ collaborating with designated staff at VCI facilities to implement and promote the intervention. Outcomes were assessed at the individual patient level.

### Study Sites 

During a 6-month pre-implementation phase, lead study investigators (KHS, JMP, JKM) conducted virtual meetings with VA facility leadership in the Sierra Pacific and South-Central VA regions to present the trial’s rationale, objectives, methodology, and facility-level randomization process. Investigators outlined expectations for facilities randomized to the VCI. This involved identifying at least one VA staff member (e.g., Health Behavior Coordinator) at each intervention facility to serve as an internal facilitator to collaborate with the research team (external facilitators) in encouraging staff (who voluntarily agreed) to participate in a virtual MI educational session on vaccine communication. Facilities assigned to the UC group had no additional requirements beyond reporting their ongoing vaccine promotional activities, a requirement of VCI facilities as well. The trial was approved by the VA Central Institutional Review Board (IRB)**,** and VA facility leaders provided informed consent for facility participation via email prior to enrollment.

### Randomization/Blinding

Randomization was conducted at the level of enrolled VA healthcare facilities, which included their affiliated community-based outpatient clinics. To attempt to balance across study sites, randomization was stratified by VA region (i.e., Sierra Pacific and South-Central US) and constrained on baseline COVID-19 primary series vaccination completion rates at each enrolled facility.^24^ Veteran patients were unaware of their facility’s random assignment. While VA providers and most research staff were unblinded to group allocation, study statisticians remained blinded.

### Implementation of the Intervention

#### Vaccine Communication Educational Intervention (VCI)

Using implementation facilitation, research team members collaborated with designated internal facilitators at VCI facilities to schedule and promote the MI-based training on vaccine communication for VA healthcare providers and staff across primary care, mental health, nursing, pharmacy, and other disciplines, including administrative staff, i.e., schedulers, peer counselors, health coaches etc. The MI-based training was developed by research team members with expertise in MI communication training (JKM, JMP, MM, BG, BB) and was delivered virtually by local VA staff with MI training expertise (Supplement: e-Fig. 1, eFigure2, Training Video). Vaccine communication trainings were often integrated into existing virtual meetings to maximize attendance. The training introduced MI techniques designed to improve vaccine-related conversations, including skills such as reflective listening, non-judgmental communication, affirming patient autonomy, and seeking permission before sharing vaccine information. Virtual trainings encouraged learner participation (e.g., responding to scenarios) to enhance skill development. To practice MI communication techniques, virtual synchronous VCI follow-up training sessions were offered monthly throughout the 1-year trial period.

#### Usual Condition (UC)

UC and VCI facilities continued usual VA-wide COVID-19 vaccination promotional activities. This involved having a COVID-19 coordinator at each facility to oversee and provide training and education on COVID-19 vaccination, including encouraging vaccination in staff and patients. To track the availability of COVID-19 (and flu) vaccines and vaccine-related promotional activities at facilities, UC and VCI facility contacts were surveyed at baseline and 3 and 6 months, and data were collected using REDCap.^25^

### Data Sources

Study outcomes were assessed at the individual patient level using VA administrative data. The primary source was the VA Corporate Data Warehouse (CDW), a comprehensive repository containing all VA electronic health records, as well as non-VA COVID-19 and flu vaccination records. These included the VA COVID Shared Data Resource (CSDR), a dataset tracking COVID-19 vaccinations within the VA system and the VA Information Resource Center (VIReC) Medicare COVID-19 Vaccination Analytic File, a dataset capturing Medicare claims for COVID-19 vaccines administered outside the VA. CDW also included vaccine records from the IZ Gateway, a system that queries most state Immunization Information Systems (IIS), triggered when patients receive care at VA facilities. While IZ Gateway records were used in sensitivity analyses, they were excluded from primary analyses due to their availability only after trial completion (July 2023) and potential for ascertainment bias among veterans with higher VA healthcare utilization.

### Outcomes

Vaccination events were counted if they occurred after the first Pfizer vaccine received Food and Drug Administration (FDA) emergency use authorization (December 11, 2020) and included vaccines from Moderna, Pfizer, Johnson & Johnson, and Novavax.^21^ The main analyses included veterans who had at least one clinical visit at an enrolled VA facility within 1 year prior to trial start and at least one primary care visit during the 1-year study period (*N* = 338,718).

The primary outcome was receipt of at least one dose of *any* approved COVID-19 vaccine (either primary series or booster) during a 12-month follow-up period. This outcome was selected as primary because distinguishing between primary series and booster doses was subject to misclassification, particularly for veterans receiving COVID-19 vaccinations outside the VA. The secondary outcome was completion of the initial COVID-19 primary vaccine series among previously unvaccinated veterans. Exploratory outcomes were receipt of one or more COVID-19 booster doses among those eligible, and receipt of the seasonal flu vaccine (as a spill-over effect from the COVID-19 vaccine-focused intervention).

### Sample Size

Power calculations were based on enrollment of ten parent VA facilities, each with an estimated nine to ten associated community clinics and an average of at least 1000 veterans per clinic. Sample size calculation is described in detail elsewhere.^21^

### Statistical Analysis

All analyses followed intention-to-treat principles. Variables were summarized using descriptive statistics. Multi-level multi-variable logistic regression models (GLMM) regressed vaccination outcomes on study arm and variables used in covariate-constrained randomization, i.e., facility baseline average COVID-19 primary series rates and geographical region. Other covariates, i.e., patient age, race, ethnicity, birth sex, and number of primary care visits 1 year prior to study start, were included a priori based on their potential impact on vaccination. We incorporated random effects for patients nested within health care clinics within parent facilities (i.e., three-level hierarchy). Odds ratios, *p*-values, and 95% confidence intervals (CI) were reported where hypothesis tests were two-sided at the 0.05 level of statistical significance. All analyses were performed using R version 4.4.1 (https://cran.r-project.org/).^26^ Separate sensitivity analyses were conducted by refitting the regression model after (1) removing the eligibility constraint of at least one primary care visit during follow-up, (2) removing Medicare records, (3) including IZ gateway records, (4) reassigning small facilities to parent VAMCs, (5) adjusting for misclassified vaccinations, and (6) removing health care utilization.

## RESULTS

**Figure 1:**
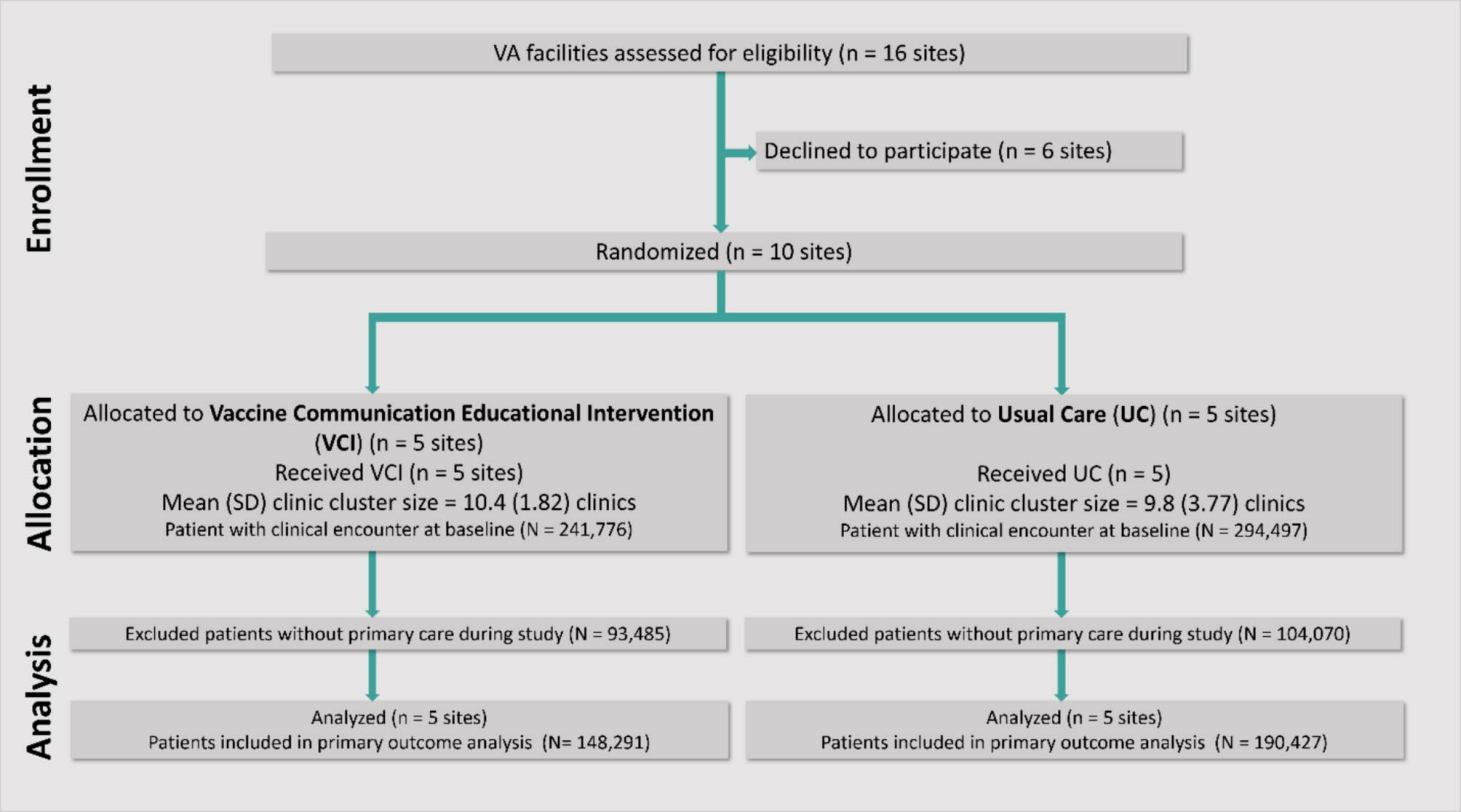
CONSORT diagram showing enrollment, allocation, and analysis of VA healthcare facilities (and patients in facility clinics) in the cluster randomized controlled trial, February 2022-May 2023**.**

Ten of 16 VA healthcare facilities in the Sierra-Pacific and South-Central regions consented to trial participation and were enrolled and randomized. This represented six of eight VA facilities from the Sierra-Pacific region and four of eight VA facilities from the South-Central region. Six facilities were either non-responsive or declined to participate citing time and staffing constraints during the pandemic. Of the ten enrolling facilities, five were randomized to VCI (including 32 Sierra-Pacific and 20 South-Central clinics, *N* = 148,291 patients) and five facilities were randomized to UC (including 27 Sierra-Pacific and 22 South-Central clinics, *N* = 190,427 patients) (Fig. 1).

Across the five VCI facilities, 1,575 providers and staff were trained in 45 separate virtual trainings, with an average of 9.2 trainings per facility (range: 5–12 trainings) and 315 participants trained per facility (range 71–413 participants). Trainings ranged from 10 to 120 min depending on facility preference and constraints during COVID-19; monthly optional trainings were poorly attended. Surveys of the ten VA facilities confirmed that background COVID-19 vaccine access, availability, and promotional activities were well-balanced between arms (Supplement: eTable 1).

The median age was 67 years [interquartile range (IQR) = 53–74 years], primarily male (88.6%), White (63.6%), and urban dwelling (68.6%). The baseline COVID-19 primary series completion rate was 78.2%. Despite randomization, there were differences in baseline characteristics. In the UC arm, a higher proportion of veterans were White, lived in the South-Central region, and had greater primary care utilization. In contrast, the VCI arm had a higher proportion of Black or African American and Sierra-Pacific region veterans (Table 1).

**Table 1:** Baseline Characteristics of 338,718 Veterans at 10 VA Health Care Systems, February 2022

	Usual care (*N* = 190,427)	Intervention (*N* = 148,291)	Overall (*N* = 338,718)
	*N* (%)	*N* (%)	*N* (%)
Patient age, yrs			
Median [interquartile range (IQR)]	67.0 [52.0, 75. 0]	67.0 [53.0, 74.0]	67.0 [53.0, 74.0]
Patient sex^€^			
Female	18,094 (9.5%)	14,406 (9.7%)	32,500 (9.6%)
Male	168,777 (88.6%)	131,215 (88.5%)	299,992 (88.6%)
Race			
American Indian or Alaska Native	1,810 (1.0%)	1,081 (0.7%)	2,891 (0.9%)
Asian	3,975 (2.1%)	8,586 (5.8%)	12,561 (3.7%)
Black or African American	32,826 (17.2%)	33,337 (22.5%)	66,163 (19.5%)
Native Hawaiian or Other Pacific Islander	1,839 (1.0%)	1,097 (0.7%)	2,936 (0.9%)
White	132,860 (69.8%)	85,508 (57.7%)	218,368 (64.5%)
More than one race	2,329 (1.2%)	3,124 (2.1%)	5,453 (1.6%)
Declined, unknown, or missing	14,502 (7.6%)	10,318 (7.0%)	24,820 (7.3%)
Ethnicity^€^			
Not Hispanic or Latino	161,567 (84.8%)	129,305 (87.2%)	290,872 (85.9%)
Hispanic or Latino	17,336 (9.1%)	9,005 (6.1%)	26,341 (7.8%)
Unknown	8,869 (4.7%)	8,264 (5.6%)	17,133 (5.1%)
VA region			
South Central VA Health Care Network	106,494 (55.9%)	62,528 (42.2%)	169,022 (49.9%)
Sierra Pacific Network	83,933 (44.1%)	85,763 (57.8%)	169,696 (50.1%)
Rurality^€^			
Highly rural	1,514 (0.8%)	1,399 (0.9%)	2,913 (0.9%)
Rural	59,849 (31.4%)	40,958 (27.6%)	100,807 (29.8%)
Urban	129,036 (67.8%)	103,225 (69.6%)	232,261 (68.6%)
Baseline primary series completion			
No	45,291 (23.8%)	28,626 (19.3%)	73,917 (21.8%)
Yes	145,136 (76.2%)	119,665 (80.7%)	264,801 (78.2%)
Primary care utilization, prior year (tertiles)			
0–1 primary care visits	76,330 (40.1%)	71,949 (48.5%)	148,279 (43.8%)
2 primary care visits	44,486 (23.4%)	33,706 (22.7%)	78,192 (23.1%)
3–118 primary care visits	69,611 (36.6%)	42,636 (28.8%)	112,247 (33.1%)
Care Assessment Need (CAN) 1-year event probability			
Median [IQR]	0.11 [0.01, 0.97]	0.11 [0.013, 0.97]	0.11 [0.013, 0.98]

^€^Unknown or declined to answer < 2% is not shown for tabular display

### Primary Outcome: Receipt of Any COVID-19 Vaccination Dose

The raw proportion of any COVID vaccinations in the year prior in VCI and UC was 79.8% and 76.9%, respectively. During the study, the raw proportions were 38.9% (VCI) and 34.4% (UC). The unadjusted differences in proportions between groups during the year prior and the study period were 2.9% and 4.5%, respectively, favoring VCI, with non-overlapping confidence intervals (Supplement: eTable 2). After adjustment, there was no significant difference between groups in receiving any dose of COVID-19 vaccine (Adjusted Odds Ratio (aOR) = 1.16, 95% CI: [0.89–1.50], *p* = 0.28). Most of the variability in receipt of any COVID-19 vaccine dose was explained by patient characteristics. Veterans who were older (≥ 50 years), resided in the Sierra Pacific region (vs. South-Central U.S.), all non-White races (except Native American/Alaska Natives), and Hispanic or Latino patients had greater odds of receiving any COVID-19 vaccination dose. Having had a greater number of primary care visits in the prior year was positively associated with higher odds of receiving any COVID-19 vaccine dose (Table 2).

**Table 2:** Receipt of Any COVID-19 Vaccine Dose Multi-level Multi-variable Logistic Regression Effect Estimates in 338,718 Veterans at 10 VA Health Care Facilities

	Receipt of any COVID-19 vaccine dose (*N* = *338,718)*	
	Odds ratio (95% CI)	*p*
Intervention arm		0.281
Usual care	1.00 (reference)	
Vaccine acceptance intervention	1.16 (0.89, 1.50)	
Baseline vaccination rate (centered)	1.01 (0.98, 1.04)	0.514
VA region		< 0.001
South Central VA Health Care Network	1.00 (reference)	
Sierra Pacific Network	1.91 (1.42, 2.57)	
Age		< 0.001
≤ 49	1.00 (reference)	
50–74	4.02 (3.92, 4.12)	
75–120	6.39 (6.22, 6.57)	
Race		< 0.001
White	1.00 (reference)	
Black or African American	1.62 (1.59, 1.65)	
American Indian/Alaska Native	0.95 (0.88, 1.03)	
Native Hawaiian/other Pacific Islander	1.16 (1.10, 1.22)	
Asian	1.81 (1.74, 1.89)	
More than one race	1.26 (1.18, 1.33)	
Declined, unknown, or missing	1.07 (1.03, 1.11)	
Ethnicity		< 0.001
Not Hispanic or Latino	1.00 (reference)	
Hispanic or Latino	1.18 (1.14, 1.22)	
Declined to answer	0.92 (0.86, 0.98)	
Unknown	1.05 (1.01, 1.09)	
Sex		< 0.001
Female	1.00 (reference)	
Male	0.99 (0.97, 1.02)	
Unknown sex	0.51 (0.47, 0.55)	
Baseline primary care utilization, prior year (tertiles)		< 0.001
0–1 primary care visits	1.00 (reference)	
2 primary care visits	1.34 (1.32, 1.37)	
3–118 primary care visits	1.74 (1.71, 1.77)	

### Secondary Outcome: Completion of COVID-19 Primary Series

The VCI was not associated with completing the COVID-19 primary series compared to UC (aOR = 0.94, 95% CI: [0.67–1.32], *p* = 0.71). Black or African American and Asian races had significantly higher rates of primary series completion compared to White patients (Table 3).

**Table 3:** Completion of Primary Series and Receipt of COVID-19 Booster Dose Multi-level Multi-variable Logistic Regression Effect Estimates in Veterans at 10 VA Health Care Facilities

	Completion of primary series *(N* = *73,843)*		Receipt of COVID-19 booster dose *(N* = *259,988)*	
	Odds ratio (95% CI)	*p*	Odds ratio (95% CI)	*p*
Intervention arm		0.706		0.477
Usual care	1.00 (reference)		1.00 (reference)	
Vaccine acceptance intervention	0.94 (0.67, 1.32)		1.10 (0.85, 1.43)	
Baseline vaccination rate (centered)	1.06 (0.88, 1.27)	0.542	1.00 (0.98, 1.03)	0.824
VA region		0.003		< 0.001
South Central VA Health Care Network	1.00 (reference)		1.00 (reference)	
Sierra Pacific Network	1.79 (1.22, 2.61)		1.93 (1.44, 2.58)	
Age		< 0.001		< 0.001
≤ 49	1.00 (reference)		1.00 (reference)	
50–74	0.71 (0.61, 0.82)		3.68 (3.58, 3.78)	
75–120	0.76 (0.60, 0.96)		5.03 (4.88, 5.18)	
Race		< 0.001		< 0.001
White	1.00 (reference)		1.00 (reference)	
Black or African American	1.80 (1.50, 2.15)		1.46 (1.43, 1.50)	
American Indian/Alaska Native	1.10 (0.59, 2.05)		1.03 (0.94, 1.13)	
Native Hawaiian/Other Pacific Islander	1.21 (0.75, 1.93)		1.08 (1.02, 1.14)	
Asian	2.78 (2.01, 3.84)		1.56 (1.49, 1.63)	
More Than One Race	1.73 (1.13, 2.67)		1.22 (1.15, 1.31)	
Declined, Unknown, or Missing	1.38 (1.07, 1.77)		1.05 (1.01, 1.09)	
Ethnicity		0.124		< 0.001
Not Hispanic or Latino	1.00 (reference)		1.00 (reference)	
Hispanic or Latino	1.17 (0.93, 1.49)		1.13 (1.09, 1.17)	
Declined to answer	0.72 (0.39, 1.35)		0.93 (0.86, 1.00)	
Unknown or missing	1.26 (0.96, 1.64)		0.99 (0.95, 1.03)	
Sex		0.233		< 0.001
Female	1.00 (reference)		1.00 (reference)	
Male	0.86 (0.71, 1.04)		0.98 (0.95, 1.01)	
Unknown sex	0.96 (0.70, 1.32)		0.76 (0.69, 0.84)	
Baseline primary care utilization, prior year (tertiles)		0.606		< 0.001
0–1 Primary care visits	1.00 (reference)		1.00 (reference)	
Medium*	–- (––, ––)		1.27 (1.24, 1.29)	
High*	0.96 (0.84, 1.11)		1.56 (1.53, 1.60)	

^*^Health-care utilization categories for primary series completion differed from the other COVID-19 vaccine outcomes, where the bins were 0 to 1 versus 1 to 118 primary health care visits, without a "middle" category. Tertiles could not be measured for primary care visits for participants included in the analysis of primary series completion. Medium for Booster outcome was 2 to 3 visits, and High for Booster outcome was greater than 3 and up to 118 primary care visits

### Exploratory Outcomes

Patients assigned to VCI were not more likely than those in UC facilities to receive one or more COVID-19 boosters (aOR = 1.10, 95% CI: [0.85–1.43], *p* = 0.48). As with other outcomes, participant characteristics were significantly associated with increased odds of receiving COVID-19 boosters (*p*-values < 0.001) (Table 3).

The VCI did not have a spillover-over effect on increasing flu vaccination (aOR = 1.11, 95% CI: [0.98–1.26], *p* = 0.12). Having received flu vaccination in the prior year was most strongly associated with receiving flu vaccine during the trial (aOR = 7.36, 95% CI: [7.24–7.48], *p* < 0.001) (Table 4).

**Table 4:** Influenza Vaccine Receipt Multi-level Multi-variable Logistic Regression Estimates in Veterans at 10 VA Health Care Facilities

	Receipt of influenza vaccine *(N* = *338,718)*	
	Odds ratio (95% CI)	***p***
Intervention arm		0.115
Usual care	1.00 (reference)	
Vaccine acceptance intervention	1.11 (0.98, 1.26)	
Influenza vaccine in prior year	7.36 (7.24, 7.48)	< 0.001
Baseline vaccination rate (centered)	1.01 (0.99, 1.02)	0.399
VA region		0.113
South Central VA Health Care Network	1.00 (reference)	
Sierra Pacific Network	1.12 (0.97, 1.30)	
Age		< 0.001
≤ 49	1.00 (reference)	
50–74	1.88 (1.84, 1.92)	
75–120	2.39 (2.33, 2.45)	
Race		< 0.001
White	1.00 (reference)	
Black or African American	1.03 (1.01, 1.05)	
American Indian/Alaska Native	0.97 (0.89, 1.06)	
Native Hawaiian/Other Pacific Islander	1.19 (1.12, 1.26)	
Asian	1.40 (1.34, 1.47)	
More Than One Race	1.11 (1.05, 1.19)	
Declined, Unknown, or Missing	1.00 (0.97, 1.04)	
Ethnicity		< 0.001
Not Hispanic or Latino	1.00 (reference)	
Hispanic or Latino	1.14 (1.10, 1.17)	
Declined to answer	0.96 (0.89, 1.03)	
Unknown	0.99 (0.95, 1.03)	
Sex		< 0.001
Female	1.00 (reference)	
Male	0.98 (0.95, 1.00)	
Unknown sex	0.68 (0.64, 0.73)	
Baseline primary care utilization, prior year (tertiles)		< 0.001
0–1 primary care visits	1.00 (reference)	
2–3 primary care visits	0.99 (0.97, 1.01)	
4–118 primary care visits	1.07 (1.05, 1.10)	

### Sensitivity Analyses

For receipt of any COVID-19 dose (primary outcome), across all sensitivity analyses that either modified the data source or relaxed eligibility criteria, the outcomes were consistent with the main results. Incorporating the assumed rates of misclassified vaccination status also did not alter the results (Supplement, eTables 2–10).

### Adverse Events

No adverse events were reported during the trial.

## DISCUSSION

This trial demonstrated that MI-informed provider communication training delivered later in the pandemic did not significantly improve uptake of any COVID vaccine dose in patients at VCI compared to UC facilities. During the pandemic, systemic challenges to intervention implementation, persistent vaccine hesitancy in patients who remained either unvaccinated or incompletely vaccinated late in the pandemic, robust usual care vaccination activities across VA, and other factors several steps removed from the provider intervention may have attenuated intervention effects, yet our findings provide valuable insights.

Implementation challenges may have limited penetration of the VCI at intervention facilities and, in turn, attenuated the effect on patient vaccination rates. First, only ten of the 16 targeted VA facilities opted into the trial. This may reflect low acceptability among facility leadership for an optional provider educational intervention during the pandemic.^27^ Second, among VCI facilities, there were barriers to adoption. It was difficult to identify local staff to promote and conduct VCI trainings and for VA providers, tasked with additional COVID-related duties, to opt in or be released to participate in the training, which was most pronounced at rural clinics with greater staffing shortages (i.e., South-Central region).

Post-pandemic, vaccine hesitancy has become even more prevalent, possibly reflecting low levels of trust in government agencies, conflicting information about vaccine effectiveness and harms, and the overall politicization of vaccination.^5,6,28,29^ Thus, there is an ongoing need to equip clinicians with communication skills to successfully engage patients in conversations about vaccination amid ongoing infectious disease outbreaks and possible future pandemics. We selected provider MI communication training emphasizing empathic listening and shared decision-making rather than a patient educational intervention because research has demonstrated that *how* a clinician communicates about vaccines may instill more trust in vaccines than providing information.^30,31^

The CoVAcS trial started 2 years after the declaration of a global pandemic and 14 months after authorization of the first COVID vaccine.^32,33^ Following VA and public health guidance for COVID-19 vaccination ^34^, when the trial commenced, nearly 80% of VA patients in this sample (median age of 67) had already completed primary vaccination. Patients lacking primary COVID-19 vaccination at this late stage were likely persistently resistant to vaccination, which may explain the larger null effect on primary vaccine series completion.^35^ Importantly, MI has been shown to be less effective with patients with little to no ambivalence about behavioral change.^36^

Finally, by design, effectiveness was measured using patient vaccination outcomes that were several steps removed from the provider/staff educational intervention. Specifically, study outcomes depended on (1) the intervention being implemented optimally at study sites, (2) providers attending the VCI sessions, (3) providers engaging patients in MI-informed vaccine discussions with (4) high fidelity, and (5) patients agreeing to receive a COVID (and/or flu) vaccine. Specifically, given time constraints in clinical practice, separate qualitative data revealed that some providers struggled to incorporate MI-styled communication about vaccination in routine patient encounters.^37^ This trial encouraged participation of all clinician types assuming that any might discuss vaccination with patients. Nevertheless, future research might target this communication intervention to staff, such as nurses, who directly administer vaccines, or to trusted primary care providers ^9^, who are more likely to successfully engage patients in vaccine-related conversations. To decrease barriers to participation and participant burden, staff informed consent was not obtained. Thus, change in providers’ self-efficacy, skills application, or satisfaction with vaccine communication could not be evaluated. Assessing these more proximal provider-level outcomes would shed light on downstream patient outcomes.

Our results revealed several characteristics strongly associated with COVID-19 vaccine uptake, highlighting outreach targets within the VA system to improve vaccination rates later in the pandemic. Specifically, targeting younger veterans, White and non-Hispanic populations, those living in Southern states (representing more rural populations), and patients not actively engaged in primary care might improve vaccination rates. Other studies corroborate the need for outreach to younger and rural populations and engagement in primary care to increase vaccination rates.^38^ In contrast to studies conducted outside the VA ^39^, Black/African American and Hispanic veterans in this VA sample had higher vaccination rates than White, non-Hispanic patients. This may reflect that the VA system has fewer barriers to accessing healthcare for minoritized populations than other systems.^40,41^

A strength of this study was the cluster randomization design ^42^, which avoided cross-contamination at individual study sites. Access to the VA electronic health record, Medicare, and IZ Gateway data facilitated ascertainment of patient vaccination outcomes and sensitivity analyses to address potential misclassification errors, which confirmed main study findings. Limitations include a relatively small number of facilities or “clusters” (10) leading to potential selection bias and some between-group differences in patient characteristics even after randomization. Multi-level, multi-variable logistic regression models incorporating random effects addressed these baseline differences but could not account for unmeasured variability across sites that commonly occurs in pragmatic trials. The study was conducted in a primarily male veteran population, which may limit generalizability to women and non-veteran populations.

In conclusion, this trial enrolled ten VA facilities encompassing 338,718 patients and implemented an MI-informed vaccine communication educational intervention with over 1500 VA providers and staff during a global pandemic and found no significant between-group differences in COVID-19 vaccination. Lessons learned include (1) implementing an educational intervention for healthcare staff *before* or risk competing priorities during the pandemic; (2) delegating local champions to deliver MI vaccination communication training in person, in small groups to facilitate MI skills practice; (3) targeting the intervention to personnel directly involved in vaccination, who have continuity relationships with patients, and who have more dedicated time for MI-styled conversations; and (4) emphasizing the use of MI-informed communication earlier when more patients are ambivalent about vaccination and open to discussion. Considering these insights, growing vaccine hesitancy, and the effectiveness of MI communication training for other behavioral change targets,^13,14,17^ similar vaccination communication educational interventions deserve further study.

## Supplementary Information

Below is the link to the electronic supplementary material.

ESM 1 (DOCX 13.1 MB)
