# Supplementary material for: A Cluster Randomized Trial of a Vaccination Communication Educational Intervention: Impact on COVID-19 Vaccine Uptake in Veterans
Source: J Gen Intern Med. 2026 Feb 12;41(8):2217–26. doi: 10.1007/s11606-026-10209-9 (PMC13241570; doi:10.1007/s11606-026-10209-9)
Supplement: Supplementary file 1 — (DOCX 13.1 MB) [file 11606_2026_10209_MOESM1_ESM.docx]

**Contents:**

1. **Provider Vaccine Communication Educational Intervention (VCI) Protocol and Toolkit**
   1. Trial Protocol
   2. eFigure1- Provider and Staff Training Slides
   3. eFigure2- Vaccine Communication Pocket Card
   4. Link to Training Video demonstrating MI communication for vaccination communication
2. **e-Table 1:** Experience of VA facilities randomized to the Vaccine Communication Educational Intervention (VCI) vs. Usual Care (UC) arms with COVID-19 vaccine access, availability, and educational activities assessed at 0, 6, 12 months
3. **Supplementary Text, eTables 2-10:** Sensitivity analyses with description

##### **SDR 21-141: Developing and Testing a COVID-19 Vaccination Acceptance Intervention**

**Multiple PIs: Karen H. Seal, MD MPH (corresponding), Jennifer K. Manuel, PhD,**

**Jeffrey Pyne, MD**

**[Version Number 5.0 Version Date 12.15.23]**

##### **Abstract**

**Background**: The COVID-19 pandemic has resulted in significant loss of life with total case and death counts increasing daily. COVID-19 vaccines are effective in preventing symptomatic disease, severe disease, disease transmission, and death. Despite the efficacy and safety of COVID-19 vaccines, many people remain hesitant about vaccination with increased hesitancy among younger individuals (less than 50 years old), Black and Hispanic adults, and those living in rural areas. Recent surveys of Veterans indicate that approximately 35% are unsure whether they will receive a vaccine, highlighting the critical need for evidence-based motivational interventions to increase COVID-19 vaccine acceptance in this population who, without vaccination, remain at risk of severe adverse outcomes and death. The overall objective of this study is to implement and test a COVID-19 vaccine acceptance intervention that is responsive to the needs of Veterans and VA healthcare providers (HCPs) and staff. VA HCPs and PACT staff are consistently identified by patients as trusted sources of vaccine information, thereby best suited to initiate vaccine acceptance discussions with patients. **Methods**: This evidence- informed Vaccine Acceptance Intervention will be conducted in VISNs 16 and 21 as a Hybrid Type 2 pragmatic implementation-effectiveness trial, guided by the i-PARiHS framework, and using Implementation Facilitation as the overarching implementation strategy. The specific aims are as follows: Aim 1) Examine the effectiveness of a Vaccine Acceptance Intervention versus Usual Care on Veterans’ vaccination rates in a one-year cluster randomized controlled trial. The implementation team will include VISN-level external facilitators, VAMC- level internal facilitators, and clinic-level site champions to support PACT staff and Whole Health Coaches and Peers in implementing the Vaccine Acceptance Intervention with Veterans. Aim 2) Survey Veterans to understand what factors are associated with the decision to accept or not accept COVID vaccination among previously unvaccinated Veterans and through qualitative interviews, learn more about these factors and how they differ between sociodemographically and clinically diverse Veteran subgroups. Aim 3) Learn what aspects of the Vaccine Acceptance Intervention and implementation strategies were helpful and not helpful in improving Veteran vaccine acceptance by conducting mid-trial and end-of-trial qualitative interviews with VISN 16 and 21 study stakeholders using purposive sampling of VA staff and HCPs from sites with high and low vaccination rates. **Impact**: This is the first multi-site randomized controlled trial to test an MI-informed vaccine acceptance intervention that can be rapidly scaled across VA to improve COVID-19 vaccine acceptance in Veterans. Furthermore, information gained will be used to inform healthcare systems’ strategies to improve future vaccination and public health campaigns.

[Version Number 5.0 / Version Date 12.15.23] VA Central IRB Protocol Template Page **2** of **53**

#####

##### List of Abbreviations

ACTIV-3 Accelerating COVID-19 Therapeutic Interventions and Vaccines AE- Adverse Event

CAN Scores- Care Assessment Need Scores

CAVHS- Central Arkansas Veterans Healthcare System CDC- Centers for Disease Control and Prevention CDW- Clinical Data Warehouse

CEAL- Community Engagement Alliance Co-I- Co-Investigator

COVID-19- Coronavirus disease 2019 CRE- CREATE

CSHIIP- Center for the Study of Healthcare Innovation, Implementation & Policy DART- Data Access Request Tracker

DoD- Department of Defense

FDA- Food and Drug Administration

GLVA- Greater Los Angeles Veteran Affairs HBC- Health Behavior Coordinator

HCP- Health Care Provider

HIPAA- Health Insurance Portability Accountability Act HPDP- Health Promotion Disease Prevention

IF- Implementation Facilitator ICN- Integration Control Number

IIR- Investigator Initiated Research

i-PARiHS- integrated Promoting Action on Research Implementation in Health Services IRB- Institutional Review Board

MD- Doctor of Medicine MHS- Mental Health Specialty MI- Motivational Interviewing

MIRECC- Mental Illness Research Education and Clinical Center

MWCCS- Multicenter AIDS Cohort Study and the Women's Interagency HIV Study

NCP- National Center for Health Promotion and Disease Prevention NIH- National Institutes of Health

OARS- Open Questions, Affirmations, Reflections, Summaries OHE- Office of Health Equity

OI&T- Office of Information and Technology PACT- Program Aligned Care Team

PCORI- Patient Centered Office and Research Institute PEI- National Partnered Evaluation Initiative

PHD- Doctor of Philosophy PHI- Protected Health Information PI- Principal Investigator

POC- Point of Contact

PTSD- Post-Traumatic Stress Disorder

QUERI- Quality Enhancement Research Initiative RRT- Rapid Response Team

SAE- Serious Adverse Event

SARS CoV-2- Severe Acute Respiratory Syndrome Coronavirus-2

SCALE- [Veteran and Staff Perceptions of VHA Large Scale Adverse Event](http://www.hsrd.research.va.gov/research/abstracts.cfm?Project_ID=2141701615) [Communications](http://www.hsrd.research.va.gov/research/abstracts.cfm?Project_ID=2141701615) SFVAHCS- San Francisco Veteran Affairs Health Care System

SQL- Structured Query Language

UAMS- University of Arkansas for Medical Sciences UC- Usual Care

UCSF- University of California San Francisco

VA HSR&D- Veteran Affairs Health Services Research and Development VACO- Veteran Administration Central Office VAI -Vaccine Acceptance Intervention

VAMC- Veteran Affairs Medical Center

VAPHCS- Veteran Affairs Puget Sound Health Care System VEP- Veteran Engagement Panel

VHA- Veteran Health Administration

VINCI- Veteran Informatics Computing Infrastructure VISN- Veterans Integrated Service Networks

VSSC- Veterans Support Service Center

wHOPE- Whole Health Options and Pain Education study WOC- Worker without compensation

**Contents**

[1.0 Study Personnel 6](#_bookmark0)

[2.0 Introduction 7](#_bookmark1)

[4.0 Resources and Personnel 14](#_bookmark2)

- 1. [Study Procedures 20](#_bookmark3)
  2. [Study Design 20](#_bookmark4)
  3. [Recruitment Methods 23](#_bookmark5)
  4. [Informed Consent Procedures/Enrollment 25](#_bookmark6)
  5. [Inclusion/Exclusion Criteria (All Study Aims) 30](#_bookmark7)
  6. [Additional Study Methods 31](#_bookmark8)

[5.8. Study Evaluations for Aims 1, 2, and 3 37](#_bookmark9)

- 1. [Data Analysis 38](#_bookmark10)
  2. [Withdrawal of Subjects 43](#_bookmark11)
  3. [Safety Assessments 43](#_bookmark12)
  4. [Specification of Safety Parameters 43](#_bookmark13)
  5. [Methods and Timing for Assessing, Recording, and Analyzing Safety Parameters 44](#_bookmark15)

[6.2 Data Safety Monitoring 44](#_bookmark17)

[7.0 Reporting 44](#_bookmark18)

[8.0 Privacy and Confidentiality 45](#_bookmark19)

[9.0 Communication Plan 47](#_bookmark20)

[10.0 References 48](#_bookmark21)

# **1.0** **Study Personnel**

##### San Francisco VA Health Care System:

Karen H. Seal, MD, MPH (PI, SFVAHCS) [karen.seal@va.gov](mailto:karen.seal@va.gov) Jennifer K. Manuel, PhD (PI, SFVAHCS) [jennifer.manuel@va.gov](mailto:jennifer.manuel@va.gov)

##### Central Arkansas VA Healthcare System:

Jeffrey Pyne, MD (PI, CAVHS) [Jeffrey.pyne@va.gov](mailto:Jeffrey.pyne@va.gov)

##### Bedford VA Healthcare System

Rani Elwy, PhD (Co-I, Bedford VA) [rani.elwy@va.gov](mailto:rani.elwy@va.gov)

## **2.0** **Introduction**

**COVID-19 is now the leading cause of death in the U.S.** As of August 2021, there were over 35 million cases and over 600,000 deaths attributed to COVID-19 in the U.S. COVID-19 has had a devastating impact on society, with racial and ethnic minority populations most adversely affected by the pandemic.^1^ In December 2020, the FDA granted emergency use authorizations for the first COVID-19 vaccines. As of August 2021, 68% of the U.S. population ages 12 and older had received at least one dose of the vaccine and 58% are fully vaccinated.^2^ Veteran vaccination rates are lower, with recent reports citing that 47% of Veterans have received at least one dose. The emergence of vaccines has reduced COVID-19 cases and deaths, but overall acceptance of the vaccine is declining. This leaves those who remain unvaccinated at risk of severe disease and death.

**Reasons for vaccine hesitancy are multi-factorial.** The World Health Organization defines vaccine hesitancy as “a delay in acceptance or refusal of vaccination despite availability of vaccination services.”^3^ Vaccine hesitancy is complex and context specific, varying across time, place, and vaccines. It is influenced by factors such as complacency, convenience and confidence. Reasons for not getting vaccinated include barriers such as lack of transportation or access to psychological services and cognitive barriers including perceived lack of risk, need for vaccine, or social benefit, which may be more pronounced in individuals with lower levels of education,^4^ and underlying mental health and substance use disorders.^5,6^ Additional barriers include negative attitudes toward vaccines, prior negative experiences with vaccination (i.e., needle phobia), misinformation about vaccines (e.g., belief that the vaccine causes COVID), and general lack of trust in the healthcare system and government.^7^ In a recent study, the most cited reasons for COVID-19 vaccine hesitancy included (1) concerns about side effects, safety, and efficacy, and (2) the unprecedented speed with which the vaccine was developed, manufactured and approved for emergency use authorization.^8^

**Sociodemographic factors are associated with greater COVID-19 vaccine hesitancy or distrust,** including younger age (< 50 years old), Black or Hispanic ethnicity, lower education, rurality, Republican or independent political partisanship, and not having received a flu vaccine in the prior year.^8,9^ Vaccination rates are higher in White adults (50%) compared to Black and Hispanic adults (36%). Similarly, 16% of White adults say they will “Wait and see” before deciding to receive a vaccine compared to 24% of Black and Hispanic adults.^1^ The above sociodemographic data are from non-VA sources. Current Veteran vaccination rates are lower overall and vaccination rates are higher in Black Veterans compared to White and Hispanic Veterans (details on vaccination rate data for VISN 16 and 21 Veterans are in the Appendix).

**Low vaccination rates among Black and Hispanic/Latinx populations** may be attributed to greater distrust of COVID-19 vaccine safety and scientists and medical providers compared to Whites. In “Distrust, Race and Research,” a landmark 2002 JAMA Internal Medicine study, Corbie-Smith and colleagues found that compared with White Americans, Black Americans were more likely to believe that physicians would ask them to participate in harmful research, expose them to unnecessary risks, not fully explain the research, or treat them as part of an experiment without their consent.^10^ This mistrust among racial/ethnic minorities must be considered within the larger context of systemic racism as well as historical trauma associated with research and healthcare in the U.S.^11,120^

**COVID-19 vaccination rates may vary widely by geographic regions**. As of early August 2021, 46% of Veterans in VISN 16 and 58% of Veterans in VISN 21 have received at least one dose of the vaccine. Variation in vaccination rates are even more pronounced at the Veterans Administration Medical Center (VAMC) facility level. In VISN 16, 63% of Veterans in New Orleans, LA have received at least one dose compared to 39% of Veterans in Alexandria, LA. These findings are consistent with overall vaccine acceptance trends which have demonstrated higher vaccination rates in urban centers compared to rural areas.^13,14^ Lower COVID-19 vaccination rates in rural communities is concerning given that compared with their urban counterparts, rural adults are at higher risk for the chronic diseases (i.e., heart disease, COPD, diabetes, obesity, cancer)^15^ that are also associated with worse COVID-19 clinical outcomes.^16^ In rural communities, racial and ethnic disparities in access to care and health outcomes persist,^17^ which may lower vaccination rates further.

**Healthcare providers’ (HCP) recommendations about vaccination is strongly associated with patients’ acceptance of vaccination.**^18^ In VA, HCPs include all members of the Patient Aligned Care Teams (PACT), i.e., the medical providers (physicians and nurse practitioners), registered nurse (RN) care managers, licensed vocational nurses, medical support assistants, social workers, dieticians, pharmacists, and mental health providers embedded in primary care. HCPs are more likely to recommend vaccination to patients if they were vaccinated themselves, hold positive attitudes about vaccination, are knowledgeable about vaccination (e.g., understand and can articulate efficacy, safety and value), and are confident in leading vaccination conversations with patients. Thus, ***provider trainings in communication*** strategies may be an important strategy in increasing vaccine uptake in patients, especially as vaccination rates have been declining in recent months.^19^

**Scientists speculate that COVID-19 may become an endemic virus^20^ and boosters may be required to protect individuals from variants and declines in immunity over time.** Current studies show that two doses of the mRNA vaccines- those produced by Pfizer and Moderna- confer more than 90% efficacy and that immunity lasts at least six months after **completing vaccination.^21,22^** The Johnson and Johnson single-dose vaccine has similar effectiveness and durability.^23^ Nevertheless, all three companies are currently testing COVID-19 boosters. This is because researchers suspect that immunity might wane over time, such as after one year or more, and might not provide sufficient protection against coronavirus variants that could emerge and evolve. Therefore, a vaccinated person might need a booster dose to stay protected against the original coronavirus strain, as well as newly emerging variants, similar to the rationale for administering the seasonal flu vaccine annually. Currently, the need for and timing for COVID- 19 booster doses has not been established, and at this time, booster doses of

COVID-19 vaccine are not recommended by the CDC ([Interim Clinical Considerations for Use of](https://www.cdc.gov/vaccines/covid-19/clinical-considerations/covid-19-vaccines-us.html?CDC_AA_refVal=https%3A%2F%2Fwww.cdc.gov%2Fvaccines%2Fcovid-19%2Finfo-by-product%2Fclinical-considerations.html) [COVID-19 Vaccines | CDC](https://www.cdc.gov/vaccines/covid-19/clinical-considerations/covid-19-vaccines-us.html?CDC_AA_refVal=https%3A%2F%2Fwww.cdc.gov%2Fvaccines%2Fcovid-19%2Finfo-by-product%2Fclinical-considerations.html)). Nevertheless, in late May 2021, the U.S. Surgeon General Dr.

Vivek Murthy has told Americans to be prepared to have a COVID-19 vaccine booster within one year.^24^ One question currently under investigation is whether the booster vaccine will be the same or a different product from the original COVID-19 vaccine.^25^ Thus, if and when booster doses of COVID-19 vaccine are approved and recommended for Americans, the VA may find itself in a similar position of needing strategies to overcome similar or even greater barriers to booster dose acceptance than with the original COVID-19 vaccines. This study will use surveys and qualitative interviewing techniques to explore Veterans’ and VA staff attitudes and perceptions of COVID-19 booster vaccination.

**More immediately, the CDC has released updated guidance that the flu vaccine may be given at the same time as the COVID-19 vaccination, provided the individual is not ill at the time.**^26^ This updated guidance is because there have been no reports of reduced immunogenicity with the administration of both vaccines simultaneously, as originally thought. It is recommended that each vaccine be given at different anatomical sites (different limbs) as an intra-muscular injection to mitigate local reaction. Given concerns about co-infection with seasonal flu and COVID-19, for Veterans not yet vaccinated against COVID-19, but who regularly get flu vaccine, messaging and coaching could be targeted to encourage uptake of both vaccines and simultaneous administration for convenience.

**Preliminary Studies in addition to ongoing Phase I Pre-implementation Project**

##### 1. Telephone Motivational Coaching to Encourage Flu Vaccination and Prevent COVID- 19 Co-Infection in High-Risk Patients within the SFVAHCS: A Quality Improvement Study (10/16/20-12/7/20) (Seal and Manuel, mPIs).

**Objective:** To conduct a Quality Improvement (QI) study to examine the feasibility of motivational interviewing (MI)-informed outreach calls for seasonal flu vaccination acceptance among higher-risk unvaccinated Veterans in the SFVAHCS.

**Methods:** First, Whole Health coaches and other staff members trained in Whole Health and motivational interviewing (MI) participated in a brief training focused on using MI to encourage acceptance of the seasonal flu vaccine. The VA National Center for Health Promotion and Disease Prevention (NCP), “Moving to Vaccine Acceptance” scripts were adapted as electronic medical record (EMR) note templates that served as scripts, and upon completion, as documentation of the telephone encounter. VA administrative data were used to identify 198 Veterans in the SFVAHCS with Care Assessment Need (CAN) scores > 95 with no documentation of having received seasonal flu vaccine. After patients confirmed not having received the seasonal flu vaccine, a Whole Health coach, Peer Specialist, or other VA staff member engaged them in an MI-styled telephone conversation about their interest in and barriers to receiving flu vaccine, especially in light of risk for co-infection with COVID-19 and flu. Consistent with MI communication, coaches asked opened-ended questions, listened in a non- judgmental manner, and asked permission to share information about flu vaccine, including specific information about where and when they could receive flu vaccination.

**Results:** 488 Veterans were initially identified for the intervention. VA staff made MI-styled flu outreach calls from 10/16/20 through 12/7/20. Of the original 488 Veterans, 290 veterans reported having already received a flu vaccine (although not documented), resulting in 198 veterans remaining eligible for the intervention (see **Table 1** below). The mean age was 73.4 years (SD±11.2 years), 96% were male, 56% were rural, 70% were Caucasian, and 4% were Hispanic. Overall, 53 patients (27%) received vaccinations within 30 days of an MI coaching call. Vaccination rates within 30 days were highest among Veterans 85 and older (47%) followed by Veterans 75-84 (27%). Unexpectedly, rates were higher among Veterans who received primary care at rural versus urban facilities (40% vs. 21%) and who resided in rural areas (32% vs. 23%). Due to the small number of women and ethnic minority Veterans, we were unable to detect differences in these subgroups.

**Discussion/Limitations:** There were several limitations to this QI project including that the sample was small, single-site and a non-generalizable population of the oldest and sickest Veterans in the health care system. The rapid pace of the seasonal vaccination cycle underscored the importance of up-to-date and accurate vaccination information for prioritizing staff resources. The oldest and rural Veterans benefitted the most from the MI-styled flu outreach calls perhaps because they were the most isolated and lacked ready access to flu vaccine services.

**Conclusion/Impacts:** This QI project demonstrated the feasibility of MI-styled outreach calls to encourage flu vaccination, which might generalize to other vaccines such as COVID-19. It also demonstrated that a work force of VA Whole Health Coaches, Peer Specialists, and others trained in Whole Health can be quickly re-purposed to use their MI and coaching skills to promote a target health behavior, in this case, seasonal flu vaccination to decrease risk for COVID-19 co-infection.

##### **Table 1.** 30-Day Influenza Immunization Rates for 198 Unvaccinated Veterans Contacted (10/16/2020 – 12/7/2020)


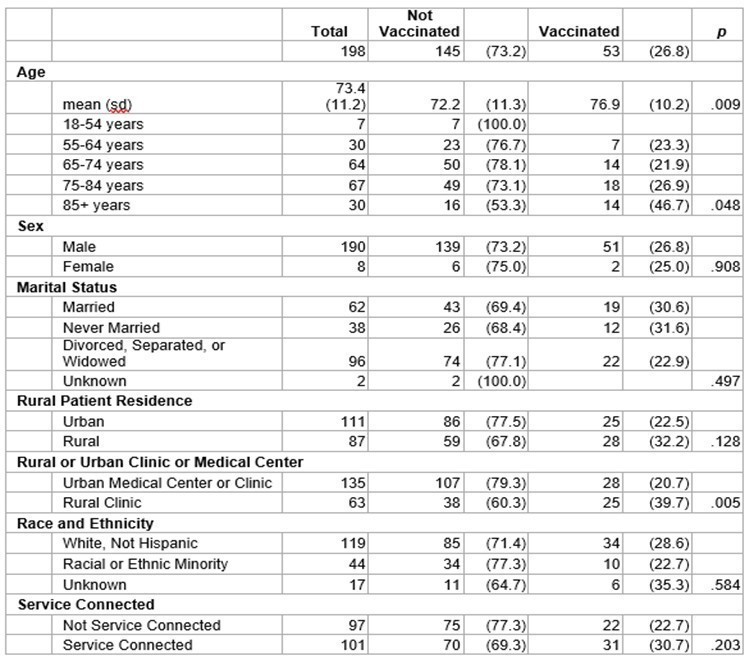


**2a. Qualitative Interviews to Understand COVID-19 Vaccine Intentions in Veterans.** Dr. Rani Elwy (Co-I), co-lead of the Bridge QUERI Rapid Response Team, led a quality improvement project to inform the National Center for Health Promotion and Disease Prevention’s vaccination efforts in VA. Semi-structured qualitative interviews (N=59) were conducted with Veterans (N=29 ) and VA staff members (N=30).

Qualitative interviews with Veterans indicated that “vaccine skepticism” was the most common form of vaccine hesitancy, **But there’s always a fear like what’s in this injection, like is it a tracker?** Lack of concern about COVID-19, or “vaccine indifference”, was cited by some Veterans, ***No, I don’t expect that I’ll get it [COVID-19]. I think if I was gonna get it I would have had it by now.***” And other Veteran stated, ***“I mean, I take care of myself when it comes to eating good and you know, taking my vitamins and things like that. So, I try to keep as healthy as possible, so if I was to contract anything my body could fight it off as best I could.”*** Distrust was another barrier to vaccine acceptance. One Veteran stated, ***“I saw it wasn’t FDA approved and I see how fast they rush this stuff.”*** Another Veteran expressed overall distrust of the system, ***“I know a lot of people who have never gotten the flu shot ever in their lives. If they did, it was because it was required of them. You know, we just don’t trust this system. It’s not that we don’t trust science.”*** Interviews highlighted the unique barriers to vaccine acceptance in the Veteran population. One Veteran stated, “…***I've been, you know, in the military and I went in the Marine corps and I was exposed to a whole bunch of chemicals and now I'm sick from them and I don't want--I don't want any more like chemicals added to my body that don't have to be added to my body***.”

**2b. Surveys of Veteran attitudes and intentions regarding COVID-19 vaccination**. Dr. Rani Elwy and the Bridge QUERI Rapid Response Team collaborated with SHEP to survey Veterans (N=1,178) through SHEP’s ongoing Veterans Insights Panel (VIP). The survey was fielded March 12-28, 2021. Of those surveyed, 29% (N=339) reported that they had not been vaccinated. Unvaccinated Veterans were asked about their intent to vaccinate and 13% reported that they would probably not receive the vaccine, 22% were unsure, and 13% said they would probably receive the vaccine. Additionally, 27% reported they would definitely not receive the vaccine and, in contrast, 25% stated they would definitely receive the vaccine.

Veterans’ self-report of their overall health varied by vaccination intention. Those who were “not sure” whether they would receive the vaccine were more likely to describe their overall health as fair/poor (43%) than those who would definitely/probably get vaccinated (30%), and 24% of those who said they would definitely/probably not get vaccinated reported fair/poor overall health. Similar trends were found regarding Veteran self-report of mental health status. Veterans who were “not sure” if they would get vaccinated were more likely to describe their mental health as fair/poor (44%) compared to those who expressed an intention to get vaccinated (37%), or remain unvaccinated (21%). Veterans cited a range of reasons for not getting vaccinated including concerns about vaccine side-effects, preference to use as few medicines as possible, vaccine distrust, healthcare system distrust, a preference to gain natural immunity (from contracting COVID-19), and a desire to wait to before making a decision about COVID-19 vaccination.

**Thus, there is a sizable proportion of unvaccinated Veterans (~35%) who remain unsure or who *might* accept COVID-19 vaccination, sometimes referred to as the “moveable middle”.** Reasons for delaying or refusing vaccine largely relate to general distrust of medicine and healthcare, and specific skepticism and distrust of the vaccine. In addition, as the pandemic continues, there is apathy, indifference, and a decreased perception of risk. This trial targets this actionable group of Veterans in testing an intervention intended to spark communication with VA providers to move patients toward vaccine acceptance.

**Risks of infection, severe disease, and death due to COVID-19 persist for the unvaccinated.** While cases, hospitalizations and death to COVID-19 are overall declining, these numbers include the approximately 60% of adults who have received at least one vaccination.^27^ When adjustments are made to include only those unvaccinated, the risk of infection is 73% higher than case rates that include both vaccinated and unvaccinated individuals.^28^ Thus, there is an urgent need for motivational interventions to increase COVID-19 vaccine uptake in the most vaccine hesitant populations **given that they remain at risk of severe disease and death due to COVID-19.** Moreover, a successful intervention must be tailored to the needs of racial/ethnic minorities, women, rural populations and high-risk individuals with mental health and substance use disorders; groups most likely to become infected, yet least likely to accept vaccination. There is a very little research of interventions to motivate vaccine acceptance. Thus, we propose to implement and test a COVID-19 vaccination acceptance intervention that is culturally and socially acceptable, focused on the behavioral factors that increase vaccine uptake, and responsive to the needs of Veterans and VA healthcare staff.

**3.0 Objectives**

The overall goal of this study is to increase COVID-19 vaccination in VISN 16 and 21 Veterans either because of vaccine hesitancy or lack of access to COVID-19 vaccination. This evidence-informed intervention will be conducted as a Hybrid Type 2 pragmatic implementation-effectiveness trial, guided by the i-PARiHS framework, and utilizing Implementation Facilitation as the overarching implementation strategy. For **Aim 1,** we will conduct a one-year cluster randomized controlled trial of a Vaccine Acceptance Intervention versus Usual Care with randomization at the level of VA Medical Center (VAMC)**.** Usual Care will consist of all national and local initiatives to promote COVID-19 vaccine acceptance in Veterans such as mobile clinics, outreach calls, public service announcements, etc. The implementation team will include VISN-level external facilitators, VAMC-level internal facilitators, and clinic-level site champions to support PACT staff, Coaches and Peers in implementing Vaccine Acceptance Intervention (VAI) communication strategies with Veterans.

In the VAI, research personnel will train and encourage PACT staff to use MI communication techniques to promote vaccine acceptance when speaking to patients about COVID-19 vaccination. In addition, we will train Whole Health coaches, Peer Specialists and other interested and qualified VA staff from VAMC intervention sites to conduct virtual and in- person motivational COVID-19 vaccination coaching outreach calls through vaccine telehubs, one in each of VISNs 16 and 21. External facilitators will also assist intervention sites in developing strategies to address site-specific barriers to COVID-19 vaccine acceptance identified by the site champions and internal facilitators. For **Aim 2**, during an 18-month period, which overlaps the trial period, each month, we will identify a diverse purposive sample of 25 Veterans distributed equally across VAMCs in VISNs 16 and 21 who have had primary care visits at Intervention and Usual Care sites (N= 450, in total), and recently received (N=360) or did not receive (N=90) the COVID-19 vaccination. We will oversample recently vaccinated Veterans to ensure a large enough sample to describe the impact of the Vaccine Acceptance Intervention on Veterans’ decision to accept COVID-19 vaccination after considering other factors. In addition, purposive sampling will prioritize Veterans who are women, ethnic/racial minorities, rural and younger individuals (< 50 years), and those with mental health conditions, including serious mental illness. In addition to surveying the larger sample of 450 Veterans, among a purposive subset of roughly 90 Veterans, we will conduct in-depth qualitative interviews to better understand the factors related to recent vaccine acceptance and persistent vaccine hesitancy.

Finally, for **Aim 3**, we will conduct process and summative interviews with study stakeholders (VA staff and providers from sites with high and low vaccination rates) to learn which implementation strategies were most and least effective.

##### Our specific aims and associated hypotheses and research questions follow:

***Specific Aim 1*** Conduct a one-year pragmatic cluster randomized controlled trial of a COVID- 19 Vaccine Acceptance Intervention to determine whether, compared to Usual Care sites, Intervention sites achieve:

- ***H1a:*** A greater incidence of Veterans receives at least ***one dose*** of COVID-19 vaccination during the study period, either as a primary vaccination or booster (***primary outcome***).
- ***H1b:*** A greater incidence of previously unvaccinated Veterans that completes COVID-19 primary vaccination (2 doses of mRNA vaccine or 1 dose of Janssen (Johnson & Johnson) vaccine).^29^

***Exploratory hypothesis H1c:*** A greater incidence of Veterans who complete seasonal flu vaccination.

***Exploratory hypothesis H1d:*** Among those eligible for a COVID booster during the trial period, a greater incidence of Veterans completes at least one booster dose.

In addition, we will utilize the VA Corporate Data Warehouse (CDW) and other VA administrative data to assess other independent predictors of vaccine acceptance (e.g., sociodemographic, clinical, and health services utilization).

***Specific Aim 2*** During an 18-month period that overlaps the trial period, conduct surveys in a sociodemographically and clinically diverse purposive sample of Veterans (N=450) with primary care visits from VISN 16 and 21 Intervention and Usual Care sites who did (N=360) and did not (N=90) receive COVID-19 vaccination. The survey will examine predictors of COVID-19 vaccine acceptance and are organized according to the World Health Organization Behavioral and Social Drivers of Vaccination (BeSD) framework.^30^ The survey includes questions from Centers for Disease Control and multiple VA surveys. In addition, in a smaller purposive subset of Veterans (N~90), we will conduct deeper-dive qualitative interviews to explore the following research questions:

 Did discussions with Veterans’ HCPs influence their decisions to accept COVID-19 vaccination?

- - *If so, how important were these discussions compared to other factors?*
  - *What was important about the HCP or these discussions regarding decision- making?*
  - *How did this vary by Veteran subgroup?*
  - *Are Veterans more likely to report engaging in discussions about COVID- 19 vaccination at Intervention compared to Usual Care sites?*

 Which factors (e.g., trust in VA HCPs, correct information about vaccines, prior experiences with VA healthcare, risk perception, perceived lack of time/access, and/ or discrimination) are most influential in Veterans’ decision-making regarding COVID- 19 vaccination?

- - *How do these factors vary by Veteran subgroup: women, younger Veterans (< age 50), Black or African American, Hispanic/Latinx and Asian Pacific Islander Veterans, rural Veterans, and those with serious mental illness?*
  - *Among Veteran subgroups, do these factors differ at VAI vs. Usual Care sites?*

 Do COVID-19 or pandemic-related impacts (e.g., COVID long-haul symptoms or other medical problems, loss of job or housing) impact COVID-19 vaccination decisions?

- - *Are some Veteran subgroups more influenced by COVID-related impacts in their decision-making re: COVID-19 vaccination?*
  - *Do these impacts differ between VAI and Usual Care sites?*

***Specific Aim 3*** Conduct mid-trial and end-of-trial qualitative interviews with VISN 16 and 21 study stakeholders, (i.e., a purposive sample of VA staff and HCPs from sites with high and low vaccination rates) to learn which aspects of the Vaccine Acceptance Intervention and which implementation strategies were helpful (or not) in improving Veteran vaccine acceptance and access. Information from mid-trial process interviews will inform intervention and implementation refinements; end-of-trial interviews will inform the development of a toolkit for future vaccine acceptance interventions.

In sum, we hope to generate evidence for an MI-informed communication intervention and implementation strategies that can be rapidly scaled beyond VISNs 16 and 21 to improve COVID-19 vaccination acceptance in Veterans across the nation. In addition, we will gain a better understanding of how to implement and disseminate interventions to address future vaccination campaigns (i.e., COVID-19 boosters) and other public health emergencies.

## **4.0 Resources and Personnel**

##### San Francisco VA Health Care System

**Karen Seal, MD, MPH (Corresponding PI, 8/8ths, SFVAHCS)** is a Professor of Medicine and Psychiatry (In Residence) at UCSF. She serves as Chief of Integrative Heath at the SFVAHCS. She is PI of several funded pragmatic implementation-effectiveness trials including an NIH- funded UG3/UH3 multi-site pragmatic trial (wHOPE) to compare two approaches to chronic pain management in Veterans (including Whole Health coaching),^31,32^ and is co-PI of a PCORI- funded study (VOICE) to test the SFVAHCS’s Integrated Pain Team approach.^33-35^ Dr. Seal has completed other trials using telehealth MI, one funded by NIH (with Drs. Borsari and Manuel) that increased uptake of complementary and integrative health strategies in Veterans with chronic pain prescribed opioids^36,32^ and two others, funded by VA which demonstrated that telehealth MI resulted in increased engagement in mental health treatment in OEF/OIF Veterans^37^ and improved mental health symptoms in rural Veterans (with Drs. Manuel, Pyne, Fortney and Mesidor).^38^ As Chief of Integrative Health, she oversees the Health Promotion and Disease Prevention (HPDP) program. In collaboration with Dr. Manuel, they trained Peers and Whole Health Coaches to conduct motivational coaching calls to several hundred high risk rural Veterans to encourage flu vaccination using NCP’s flu vaccine acceptance scripts on which the COVID-19 vaccination scripts are based. Outcome data including Veterans’ interest in COVID- 19 vaccination using a Qualtrics survey are forthcoming. Dr. Seal is also leading the SFVAHCS Vaccine Outreach and Education Team responsible for developing a multi-pronged approach to increasing COVID-19 vaccination uptake in SFVAHCS employees and Veterans. These experiences plus her skillsets in pragmatic clinical trials of behavioral interventions,^32,39^ implementation science,^31,40,41^ and secondary data analysis using the VA EHR^42-45^ support her leadership of the proposed study.

**Jennifer Manuel, PhD (PI, 8/8ths, SFVAHCS)** is an Associate Professor in the Department of Psychiatry and Behavioral Sciences at UCSF and is based at SFVAHCS where she is a Clinical Research Psychologist, Deputy Director of Psychology and Health Behavior Coordinator in the HPDP program. Relevant experience includes serving as PI and Co-I on multiple trials examining the implementation of evidence-based treatments in front-line clinical settings.^46,47^ She serves as PI on an HSR&D grant examining the quality of care provided through VHA and the community, which includes qualitative research on the impact of COVID- 19 on the delivery of care to Veterans. Dr. Manuel has over 15 years of experience training medical providers in MI and previously worked for VA Central Office’s national MI training program where she monitored MI training outcomes and fidelity among frontline VA clinicians.^48,49^ In addition, Dr. Manuel serves on SFVAHCS’s Health Equity Council and has published on adaptations of brief interventions for diverse populations.^36,50^

##### Central Arkansas VA Healthcare System:

**Jeff Pyne, MD (PI, 8/8ths, CAVHS)** is a Navy Veteran, Professor of Psychiatry at UAMS, based at CAVHS, where he serves as the Associate Director for Research at the South-Central MIRECC and Director of the HSR&D CREATE “Improving Rural Veterans' Access/Engagement in Evidence-Based Mental Healthcare”. Relevant experience includes PI, Co-PI, or site PI of multiple projects focused on rural veterans, multi-component telemental health interventions, implementation science (RVR 19-478, PCS-1406-19295, QUE 15-282, CRE 12-083, SDP 10-

044, MHI 08-098-1, MH076908-04, MNT 05-152, NPI-01-006-1, IIR 00-0781),^41,63-65^ community-

based participatory research (I01 HX-002313-01, C00863),^66-68^ think-aloud survey development (IIR 03-257-1),^69,70^ and access to care survey development (CRE 12-300).^71-76^

#####

##### Bedford VA Healthcare System

**A. Rani Elwy, PhD (Co-I, 8/8ths, Bedford VA)** is an Associate Professor at Brown University. At the Bedford VA she is Multiple-PI of the Bridge QUERI Program and co-lead of Bridge QUERI’s Rapid Response Team (RRT), commissioned to evaluate needs for the COVID-19 Vaccine Integrated Project Team in VACO, and NCP. The “QUERI RRT-3” are examining Veteran and employee vaccine hesitancy through surveys, interviews and focus groups, and using these data to develop communication and implementation strategies to increase vaccine acceptance. Dr. Elwy also leads studies of patient-provider-system level risk communication to Veterans and their families. Dr. Elwy's work on examining surgeons' disclosures of clinical adverse events, published in JAMA Surgery, received the HSR&D Best Paper Research Award in 2017. Her HSR&D funded study (SCALE, SDR 11-440) led to the development of the Large- Scale Disclosure Toolkit, incorporated into VHA Directive 1004.08, "Disclosure of Adverse Events to Patients". Dr. Elwy is a member of the VACO Clinical Episode Review Team, where she delivers the toolkit through implementation facilitation to VHA facilities in need of support in communicating with Veterans, family members, Veterans Service Organizations, and VA employees when things go wrong during the course of delivering health care.^78-80^

## **Study Procedures**

## **Study Design**

This evidence-informed intervention will be conducted as a Hybrid Type 2 pragmatic implementation-effectiveness trial, guided by the i-PARiHS framework, utilizing Implementation Facilitation as the overarching implementation strategy. For **Aim 1,** we will conduct a one-year cluster randomized parallel group trial, stratified by VISN, using covariate constrained randomization (e.g., rural/urban clinics, facility size).^81^ Specifically, we will compare a Vaccine Acceptance Intervention versus Usual Care with randomization at the level of VA Medical Center (VAMC), with the intervention directed at VAMC clinic and CBOC PACT staff**.** Usual Care will consist of all VA and other national and local initiatives to promote COVID-19 vaccine acceptance such as mobile clinics, outreach calls, public service announcements, etc. All VA and non-VA Usual Care initiatives will be documented and described in detail through quarterly environmental scans at all study sites.

The Vaccine Acceptance Intervention will be implemented using external facilitation by the research team in partnership with trained external facilitators in VISNs 16 and 21. External facilitators will partner with VAMC-level internal facilitators and clinic-level site champions to implement the Motivational Interviewing (MI)-informed Vaccine Acceptance Intervention (VAI). The implementation team will use VISN-level external facilitators, VAMC-level internal facilitators, and clinic-level site champions to support PACT staff and Coaches in implementing the Vaccine Acceptance Intervention communication strategies with Veterans.

Through the VAI, PACT staff will be trained and encouraged to use MI communication techniques to promote vaccine acceptance when speaking to patients about COVID-19 vaccination. In addition, we will train Whole Health coaches, Peer Specialists and other interested and qualified VA staff from VAMC intervention sites to conduct virtual and in- person motivational COVID-19 vaccination coaching calls using NCP’s “Moving to COVID-19 Vaccine Acceptance” scripts (see VAI training manual) through vaccine telehubs, one in each of VISNs 16 and 21. External facilitators will also assist intervention sites in developing strategies to address site-specific barriers to COVID-19 vaccine acceptance identified by the site champions and internal facilitators. For **Aim 2**, during an 18-month period, overlapping the trial period, each month, we will identify a diverse purposive sample of 25 Veterans distributed across VAMCs VISNs 16 and 21 with primary care visits at Intervention and Usual Care sites (N=450, in total), who recently received (N=360) or did not receive (N=90) the COVID-19 vaccine. Purposive sampling will prioritize Veterans who are women, ethnic/racial minority Veterans, rural and younger individuals (< 50 years), and those with mental health conditions, including serious mental illness. We will survey the larger sample of 450 Veterans, and among a purposive subset of roughly 90 Veterans, we will conduct in-depth qualitative interviews to better understand factors related to recent vaccine acceptance and persistent vaccine hesitancy. Finally, for **Aim 3**, we will conduct process and summative qualitative interviews with study stakeholders (VA staff and providers from sites with high and low vaccination rates) to learn which implementation strategies were most and least effective. (See **Figure 1. Study Design**, below).

##### **Figure 1. Study Design and Timeline**


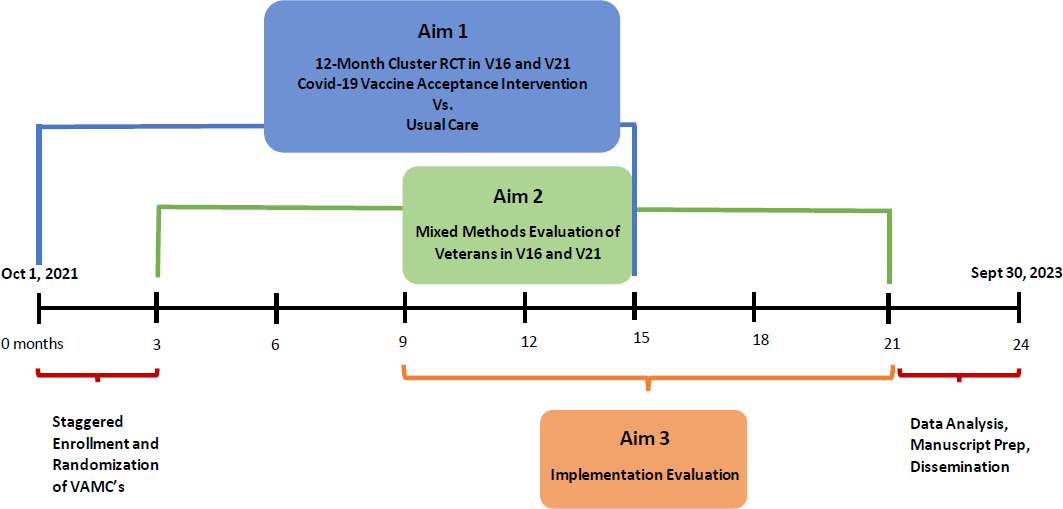


- - 1. **Risk vs. Benefit:** Risk in this study is considered minimal. Potential risks include the following for participants: (1) VA staff: (a) concern about retribution or job loss for voicing opinions about implementation of their VAMC’s vaccination program or study intervention; or

(b) time away from regular duties to participate in the study as a stakeholder, internal facilitator or clinic champion or other study collaborator providing MI training, etc.; (2) Veterans participating in study interviews: (a) loss of privacy, (b) time required to participate in the study, or (c) some questions may cause mild discomfort. Benefits to study participation may mitigate potential risks: (1) For VA staff: (a) pride or satisfaction from working on high-priority area for VA; (b) participating in research to promote COVID-19 vaccination; (2) Veterans participating in interviews: (a) questions included in surveys do not cause more discomfort than those encountered in the course of routine clinical care or everyday life; (b) Veterans participating in the cohort survey or interviews will be renumerated for their time and effort; and (c) participating in research represents an opportunity to help other Veterans and Americans.

- - 1. **Study Population:** Each of the three study aims involves different study populations:

***Aim 1:*** This cluster RCT (with randomization at the site level) will be conducted at a maximum of 16 VAMCs or systems in VISNs 16 and 21: up to 8 VAMCs in each VISN that are willing and eligible to participate in the trial. (**See Table 2 below for specific study site locations; see Appendix Tables 1-3 for more detailed information about each study site**). VISNs 16 and 21 cover broad regions of the U.S. affording considerable geographic diversity. VISN 16 includes VAMCs and systems located in Arkansas, Louisiana, Mississippi, and Texas and VISN 21 covers Northern and Central California, Nevada, and Hawaii. This trial will include primary care clinics within each VAMC (i.e., Medical Practice, Geriatrics, Women’s Clinic, Infectious Disease/HIV Clinic), as well as community-based outpatient clinics (CBOCs) and the patient- Aligned Care Team (PACT) staff within each CBOC and clinic. Aim 1 will focus on Veterans who have had a primary care visit within the past year at one of the primary care clinics or CBOCs affiliated with each of the participating VAMCs in VISNs 16 and 21. The primary aim of receiving any COVID-19 vaccination includes all veterans regardless of prior vaccination status while the secondary aim of primary vaccine series completion is restricted to veterans who remained unvaccinated at the start of the trial.

##### **Table 2: VAMCs and systems participating in the COVID Vaccine Acceptance Trial**

| **Name** | **VISN** | **Station ID** | **Type** | **Location** | **State** |
| --- | --- | --- | --- | --- | --- |
| Alexandria VA Health Care System | 16 | 502 | VAMC | 2495 Shreveport Hwy 71 N Pineville, LA 71360-4044 | LA |
| Central Arkansas Veterans Healthcare System  Eugene J. Towbin Healthcare Center | 16 | 598 | VAMC | 200 Fort Roots Drive North Little Rock, AR 72114-170 | AR |
| Central Arkansas Veterans Healthcare System John L.  McClellan Memorial Veterans Hospital | 16 | 598 | VAMC | 4300 West 7th Street Little Rock, AR 72205-5484 | AR |
| G.V. (Sonny) Montgomery VA Medical Center | 16 | 586 | VAMC | 1500 E. Woodrow Wilson Avenue Jackson, MS 3921 | 6 MS |
| Gulf Coast Veterans Health Care System | 16 | 520 | VAMC | 400 Veterans Avenue Biloxi, MS 39531 | MS |
| Michael E. DeBakey VA Medical Center | 16 | 580 | VAMC | 2002 Holcombe Blvd. Houston, TX 77030-4298 | TX |
| Overton Brooks VA Medical Center & Shreveport | 16 | 667 | VAMC | 510 E. Stoner Ave. Shreveport, LA 71101-4295 & | LA |
|  |  |  |  | 510 E Stoner Ave Shreveport, LA 71101 |  |
| Southeast Louisiana Veterans Health Care System | 16 | 629 | VAMC | 2400 Canal Street New Orleans, LA 70119 | LA |
| Veterans Health Care System of the Ozarks | 16 | 564 | VAMC | 1100 N. College Avenue Fayetteville, AR 72703 | AR |
| VISN 16: South Central VA Health Care Network | 16 | 10N16 | VISN | 715 S. Pear Orchard Road Ridgeland, MS 39157 | MS |
| Central California VA Health Care System | 21 | 570 | VAMC | 2615 E. Clinton Avenue Fresno, CA 93703 | CA |
| Livermore & Menlo Park | 21 | 640 | VAMC | 4951 Arroyo Road Livermore, CA 94550 | CA |
|  |  |  |  | 795 Willow Road Menlo Park, CA 94025 |  |
| San Francisco VA Health Care System | 21 | 662 | HCS | 4150 Clement Street San Francisco, CA 94121 | CA |
| VA Northern California Health Care System | 21 | 612 | VAMC | 10535 Hospital Way Mather, CA 95655 | CA |
| VA Pacific Islands Health Care System | 21 | 459 | VAMC | 459 Patterson Road Honolulu, HI 96819-1522 | HI |
| VA Palo Alto Health Care System | 21 | 640 | VAMC | 3801 Miranda Avenue Palo Alto, CA 94304-1290 | CA |
| VA Sierra Nevada Health Care System | 21 | 654 | VAMC | 975 Kirman Avenue Reno, NV 89502 | NV |
| VA Southern Nevada Healthcare System | 21 | 593 | VAMC | 6900 North Pecos Road N. Las Vegas, NV 89086 | NV |
| VISN 21: Sierra Pacific Network | 21 | 10N21 | VISN | 391 Taylor Blvd., Suite 200 Pleasant Hill 94523 | CA |

***Aim 2:*** Sociodemographic characteristics of vaccinated and unvaccinated Veterans in VAMCs in VISNs 16 and 21 in June 2021 are shown in the **Appendix (Table 2 and 3).** Notably, among Veterans 18-50, on average, 81% remain unvaccinated against COVID-19; 67% women vs.

63% of men are unvaccinated; White and native populations have higher proportions of unvaccinated (67% and 63%-68% respectively) compared to African American/Black veterans (57.4%). In addition, there are greater numbers of unvaccinated Veterans among rural populations (68%) vs. 61% unvaccinated urban Veterans. In addition, not surprisingly, those who remain unvaccinated have had fewer mean outpatient visits over the past year compared to Veterans who have been vaccinated against COVID-19 (5.99 +/- 8.5 vs. 8.23 +/- 10.6 mean outpatient visits); health services utilization has been associated with receipt of vaccination.^82,83^ Very importantly, these VA datasets showing the frequencies of unvaccinated VA patients are likely missing some proportion of Veterans who received the COVID-19 vaccination outside VA, but have either not reported their COVID-19 vaccination status to VA, or VA has not captured vaccine status through data-sharing with other vaccine providers such as state and county health departments, contracted businesses (e.g., Walgreens), or other health care providers

(e.g., Health Maintenance Organizations for dually insured Veterans and private offices). We received permission from the VA CIRB (in the Phase I pre-implementation application) to access Medicare data to capture some of this missing COVID-19 vaccine data. In addition, during the pre-implementation phase, we are actively seeking additional COVID-19 vaccination data sources to improve our team’s ability to accurately determine Veterans’ COVID-19 vaccine status. Moreover, because there are likely already vaccinated Veterans showing as unvaccinated in VA databases, and there is no literature to indicate what biases may impact vaccine reporting, the sociodemographics by vaccination status will also likely change as we more accurately define our denominator of unvaccinated Veterans.

Despite these limitations, given what we already know of the sociodemographic composition of Veterans in VA healthcare, we can anticipate the need for oversampling in order to interview a diverse population of Veterans. For instance, compared to the general U.S. population, women are underrepresented in Veteran samples. Thus, to achieve better representation of women, we will oversample women by inviting *all* preliminarily eligible women and only a proportion of preliminarily eligible men (target at least 20% women veterans). We will monitor enrollment of ethnic minority populations, relatively younger (< age 50 years), and rural Veterans and will use similar procedures to over-sample and prioritize these groups. Minimum enrollment targets for the 450 total Veterans for Aim 2 are as follows: Veterans < age 50 years (30%), Black or African American (20%), Hispanic/Latinx (15%), Asian, Pacific Islander, and Native populations (10%), and rural Veterans (40%). Some of these subgroup designations will overlap in individuals. We will monitor sociodemographic characteristics of Veterans enrolled each month in Aim 2 and adjust recruitment accordingly to achieve these targets.

***Aim 3****:* VISN 16 and 21 study stakeholders (including VA PACT clinicians and VA administrative staff (e.g., MD, APN, RN, LVN, Pharmacist, Psychologist, Dietician, Social Work, Medical Support Assistants) from primary care clinics with high and low vaccination rates will participate in qualitative interviews to learn which intervention components and implementation strategies were most and least effective through qualitative interviews. All participants will be VA employees.

- - 1. **Inclusion of Vulnerable Populations:** We do not anticipate excluding vulnerable populations with the exception of Veteran patients who, at the time of enrollment (Aim 2), are suicidal or homicidal (indicated by behavioral flags), or who demonstrate moderate to severe cognitive impairment, limiting their ability to participate in surveys and interviews. This study will not include VA staff or Veteran patients < 18 years of age or who are incarcerated at the time of enrollment because of need for special consent and access, respectively. VA employees are considered vulnerable because of the potential retaliation by supervisors and colleagues for participating or not participating in research. Therefore, VA employees’ decisions about study participation will not be shared with other VA employees or supervisors. All VA employees and Veteran patients will review information about the study in completing Informed Consent (or Study Information Sheets) and will be given the opportunity to ask questions of the research staff. In addition, all study data will remain private and confidential within the limits of confidentiality for VA research studies. To protect privacy and confidentiality, unique identifiers will be used to identify study participants and no personal identifiers will be associated with study data.

## **Recruitment Methods**

***Aim 1:*** There are 8 separate VAMCs/systems in VISN 16 and 8 separate VAMCs/systems in VISN 21 for a total of up to 16 potentially eligible VAMCs/systems. Each of these has affiliated CBOCs and primary care clinics. During the pre-implementation phase, the study team will seek

endorsement from VISNs 16 and 21 leadership to include their VAMCs and affiliated clinics and CBOCs in the Vaccine Acceptance Intervention Trial (see Informed Consent procedures/Enrollment below for more detail).

###### **Sample Determination for Aim 1**

We plan to include VAMCs, and by extension, their affiliated clinics and CBOCs. There are 136 clinics and CBOCs in VISN 16 and 21. There are 900,622 total Veterans in VISNs 16 and 21; 497,944 in V16 and 402,678 in V21. As of June 1, 2021, of those in V16, 322,596 (65%) remain unvaccinated (have not received at least one dose of COVID-19 vaccine) and in V21, 248,766 (62%) have not initiated vaccination. VISN 16 includes 8 separate VAMCs of which

G.V. (Sonny) Montgomery Department of Veterans Affairs Medical Center, Alexandria VA Medical Center, and Biloxi VA Medical Center have the lowest percentage of Veterans vaccinated against COVID-19 vaccination (**Appendix, Table 2**). VISN 21 includes 8 separate VAMCS/systems of which San Francisco VA Medical Center and North Las Vegas VA Medical Center have the lowest numbers of Veterans vaccinated against COVID-19 in the U.S. (**Appendix, Table 3**). By October 1, 2021 when the trial launches, given current vaccination rates and trends, forecast models predict that 307,000 (62%) will remain unvaccinated in V16 and 237,600 (59%) will remain unvaccinated in V21 (**Appendix, Figures 1 and 2**).

***Aim 2:*** During the 18-month trial period, using VA administrative data, each month, we will identify a sociodemographically and clinically diverse purposive sample of 450 Veterans distributed equally across VAMCs in VISNs 16 and 21 with primary care visits at Intervention and Usual Care sites since the start of the trial at their facility, who recently received (N=360) or did not receive (N=90) COVID-19 vaccination. Purposive sampling will prioritize Veterans who are women, ethnic/racial minorities, rural and younger individuals (< 50 years), and those with mental health conditions, including serious mental illness. We will survey the larger sample of 450 Veterans and, among a purposive subset of roughly 90 Veterans, we will conduct in- depth qualitative interviews to better understand factors related to recent vaccine acceptance and persistent vaccine hesitancy. Each month, we will run reports to monitor the sociodemographic and clinical composition of our enrolled sample to achieve our recruitment targets: Veterans < age 50 years (30%), Black or African American (20%), Hispanic/Latinx (15%), Asian, Pacific Islander and Native populations (10%), rural Veterans (40%), and Veterans with serious mental health conditions (15%). If it seems that we are not learning new information from the unvaccinated group over time, then we will shift those interviews to recently vaccinated Veterans resulting in more vaccinated Veteran interviews.

***Sample for Aim 2:*** The numbers needed at each stage of recruitment to enroll a total sample of 450 Veterans over 18 months is shown below, enrolling 25 Veterans per month, roughly 20 of whom are vaccinated and 5 of whom are unvaccinated (**Table 3**). Justification for over-sampling recently vaccinated Veterans is to ensure a large enough sample to describe the impact of the Vaccine Acceptance Intervention on Veterans’ decision to accept COVID-19 vaccination after considering other factors. The estimates for enrollment are conservative and derived from our experience recruiting for both survey studies and clinical trials in the past.

Estimating a 20% enrollment rate, with a target of N=450, if 80% of those who are phone screened are eligible to participate, and we are able to reach 35% of Veterans by phone to screen them, we would need to send out roughly 8,000 study information/opt-out postcards over 18 months (or roughly 450/month). Given that we anticipate as many as 108,000 to 160,000 unvaccinated Veterans at the start of the trial across VISNs 16 and 21, if we conservatively estimate that 5% will become vaccinated each month (or 5,400-8,000), it is feasible to recruit 25 Veterans each month for a total of 450 over 18 months and ensure that our purposive sampling targets are achieved (**see Table 3 below**). Monitoring of eligibility, contact and enrollment rates

will be performed for the study overall and by VISN weekly. The number of patients contacted each week will be adjusted to maintain overall target enrollment rates. The purposeful demographic and clinical selection could also change over time as we identify compelling sociodemographic or clinical subgroups (e.g., the youngest Veterans < 30 years, highly rural Veterans, or Veterans with substance use disorders or posttraumatic stress disorder) in the course of conducting surveys and interviews.

##### **Table 3. Plan for recruitment of an adequate sample size (Aim 2)**

|  | **18**  **Months** | **Monthly** |
| --- | --- | --- |
| Total number of study information/opt-out postcards needed | 8037 | 447 |
| Number of patients expected to be phone screened (.35) | 2813 | 156 |
| Of those screened, total number expected to be eligible (.80) | 2250 | 125 |
| Of those eligible, total number expected to enroll (.20) | 450 | 25 |

In consultation with our qualitative research team, Drs. Elwy and Purcell, we have established a target sample size of N~90 Veterans (maintaining the purposive sampling scheme outlined above) for the in-depth qualitative interviews because we anticipate being able to reach thematic saturation for the research questions posed in Aim 2 with this sample size. This subset of ~90 Veterans from the total sample of 450 Veterans, will also be identified based on purposive sociodemographic and clinical targets.

***Aim 3:*** Aim 3 involves mid-trial process interviews and end-of-trial summative interviews with VA staff and HCPs at Intervention sites to understand what worked and did not work about the intervention and implementation. If there are Usual Care sites with high vaccination rates, we will also interview staff and HCPs at those sites.

***Number of Subjects for Aim 3:*** We are planning to conduct mid- and end-of-study interviews with up to three high and three low vaccination rate clinics per VISN. The number of interviews per site will be 3 to 4 and this will result in approximately 48 mid-trial qualitative interviews and approximately 48 end-of-trial qualitative interviews. The purposive sampling of clinics will be based on the change in vaccination rate from baseline to each endpoint. In consultation with Site Champions, Internal and External Facilitators, we will choose individuals to interview who are most familiar with the intervention and/or clinic operations. We will also look for outlier sites to interview, e.g., Intervention sites with a dramatic improvement in vaccination rate from mid- to end-of-trial and Usual Care sites with high rates of change in vaccination rates.

## **Informed Consent Procedures/Enrollment**

**5.3a. Aim 1 Vaccine Acceptance Trial.** This is a cluster randomized controlled trial design and thus randomization is at the level of VAMC within VISNs 16 and. During the pre- implementation phase (Exempt protocol #E21-06), the study team will meet with leadership of VISNs 16 and 21

and their respective VAMCs to describe the study aims, methods and requirements of each study arm as well as the potential risks/burden and benefits of participation. Specifically, at these meetings, the study team will explain that there is a 50/50 chance that a VAMC will be randomized to be an Intervention or Usual Care site and what is required of each condition. A VAMC assigned to Usual Care will have no specific trial intervention requirements beyond their usual level of participation in national and local initiatives to improve COVID-19 vaccine acceptance. At both Intervention and Usual Care sites, the study team will perform quarterly “environmental scans”. The environmental scan survey will include questions about site specific barriers to COVID-19 vaccination, current programs/initiatives in the clinic or local community that are improving or have had no impact on vaccination rates, and the perceived importance that the VAMC/CBOC and clinic staff is placing on vaccination (**Environmental Scan Survey, in preparation**). At Usual Care sites, a point of contact will be chosen from each clinic and CBOC to complete the environmental scan, and at Intervention sites, the Site Champion (**see below**) will perform the quarterly scan.

A VAMC assigned to the Intervention condition will have several requirements. First, VAMC leadership will need to identify and provide release time (about 10% FTE) for a staff member who is already leading the COVID-19 vaccination effort at their site to serve as an Internal Facilitator for their VAMC. The VAMC Internal Facilitator will partner with the trial External Facilitators (research team staff designated as External Facilitators in VISNs 16 and 21) to adapt and implement the study intervention to best meet the needs and preferences of their VAMC site. In addition, each CBOC and clinic affiliated with a VAMC in the Intervention arm will need to identify a Site Champion to provide clinic-specific information to the Internal Facilitator to facilitate implementation of the Vaccine Acceptance Intervention at their clinic or CBOC. In addition, for clinics and CBOCs assigned to the Vaccine Acceptance Intervention, VAMC, clinic and CBOC leadership will need to agree to release PACT staff (who voluntarily agree) for an initial two-hour Motivational Interviewing (MI) training, and at least one 60- minute post-training consultation session over the one-year trial period. There will be additional consultation sessions offered to intervention clinics and CBOC staff, but these will be optional.

All these trial requirements will be explained at the time of the initial meetings with VISN and VAMC leadership in VISNs 16 and 21. At these meetings, in addition to the above information, the study team will answer all questions related to trial participation. The research team will also use these meetings to solicit specific information about VAMC-affiliated clinics and CBOCs to ensure eligibility to participate (primarily based on interest, adequate staffing, adequate release time for staff to participate, and having agreements in place with the VAMC to participate in research). Of note, VAMCs/systems will be able to participate as a study site even if not all VAMC-affiliated primary care clinics and CBOCs are available to participate. Further, all VA staff at participating study sites will be invited but not required to participate in the trial because research is optional and never required. There will be no negative impact for individual VA staff members who opt not to participate in activities related to the trial at an intervention clinic. As a follow-up to the meetings, we will ask the Chief of Staff (or Deputy) or the Medical Center Director (or Deputy) at each VAMC to respond to an email indicating that they agree to have their VAMC participate in the Vaccine Acceptance Study Intervention acknowledging all conditions of participation as explained in the meeting and summarized in the follow-up email.

VAMCs will also be invited to have a follow-up meeting with the study team if they have questions or concerns they wish to address prior to considering participation in the study.

Veteran vaccine status and other sociodemographic, clinical, and health service utilization data required to test Aim 1 hypotheses will be obtained through VA administrative databases. We will seek a HIPAA waiver and a waiver of Informed Consent to obtain these data. We will not seek

to obtain data from VA staff who attend meetings or trainings associated with Aim 1 unless they are specifically recruited for qualitative interviews (**see Aim 3 below**).

Randomization

This is a cluster randomized parallel group trial design in which the unit of randomization will be at the level of VAMC facility. Cluster randomization is often used to avoid contamination between those receiving the intervention and those who are not. In this case, the Vaccine Acceptance Intervention will occur at Intervention sites and not at Usual Care sites, but we will monitor for evidence of contamination, and if not able to fully guard against it (VA is a national system), we will document examples of potential contamination in the environmental scans described previously.

Randomization will be stratified by VISN (either 16 or 21) and covariate constrained by baseline primary series vaccination rates.^81^ Given the likely relatively small number of VAMCs that will be randomized (N < 16), we are selecting only one stratification variable (VISN or geographic location) and one or more covariates to constrain on. We have chosen to stratify on VISN given the central importance of location on vaccination outcomes and so as not to have all VAMCs in one VISN randomly assigned to the same study arm. We will also constrain on baseline primary series vaccination rates according to whether sites have lower or higher rates at the start of the study. The number of sites that participate will guide the feasibility of constraining on additional factors, such as rurality, because current data (see **Appendix,** Table 1) suggests that rurality is an important determinant of whether individual patients accept COVID-19 vaccination.

Blinding

The study PIs and co-investigators, other research staff (except for the study statistician, see below), qualitative interviewers and participating VA staff will be unblinded because they are involved in delivering the intervention or, in the case of the qualitative researchers, will be tasked with interviewing study stakeholders about the intervention or implementation strategies. In contrast, the study evaluators, study statistician, and Veterans at VAMC sites in VISN 16 and 21 will be blinded to study arm assignment (Vaccine Acceptance Intervention vs. Usual Care). Thus, evaluators will conduct the study survey in 450 Veterans, and while they will be aware of COVID-19 vaccination status, they will not be apprised of study arm. In addition, unless Veterans are unintentionally informed, Veterans will not know to which arm their VAMC has been randomized. Blinding of the study statistician conducting the outcomes analyses, survey evaluators, and the Veteran participants is important to protect the internal validity of study findings. Despite this being a cluster RCT, outcomes will be ascertained at the patient level.

##### 5.3.b. Aim 2 Veteran Surveys and Interviews

Additional Recruitment Methods: The VA Corporate Data Warehouse (CDW) will be used (via a HIPAA waiver for recruitment) to identify over 8,000 eligible patients over the course of 18 months for inclusion in the cross-sectional survey and qualitative interview across all participating VAMC clinics in VISNs 16 and 21. The following are the broad eligibility criteria used to search VA administrative databases for potentially eligible participants:

- - - ≥1 visit(s) at a participating VISN 16 or 21 inpatient or outpatient service after the start of the trial at their site.
    - Are members of one of two groups with respect to COVID-19 vaccination status:

(1) You have either not started COVID-19 vaccination, (2) you received your most current dose of the primary series of a COVID-19 vaccine series after clinicians at your site received the training intervention and within the past 150 days.

- No evidence of dementia using ICD-10 diagnostic codes.

Veteran eligibility data from the VA CDW will be entered into an SQL database by the study team’s data manger to which each site’s study coordinator will have access. The SFVAHCS will serve as the study’s data center. The SFVAHCS has experience in serving as a data center for other NIH- funded (the wHOPE study, Seal, PI) and VA HSR&D-funded trials (COACH, Seal PI; Pyne site PI). The SFVAHCS study coordinator and data manager will train other study staff involved in recruitment on how to use the database for recruitment and enrollment.

Potentially eligible Veterans (identified through VA administrative databases using a HIPAA waiver for recruitment) will be mailed recruitment materials: patient letters inviting them for a cross-sectional survey and possible qualitative interview, study information sheets, and opt-out postcards addressed to the SFVAHCS data center. We will wait 10 days for opt-out postcards to be returned or for interested patients to call the study staff. If subjects do not opt out or call the study staff first, study staff will contact them via phone; study staff from the Little Rock VA (CAVHS) and staff at the SFVAHCS will contact Veterans in VISN 16 and VISN 21. No cold calls will take place. The recruiter will make up to 5 attempts to reach potential participants and leave up to 3 discrete voicemails. On reaching potential participants, the study staff will attempt to determine interest in participating in the survey, verify COVID-19 vaccination status and assess eligibility using a phone eligibility screener **(see Appendix, Draft Phone Eligibility Script/Screener).** This screener will assess the following criteria:

o No consistent ability to be contacted by phone

Potential participants who remain eligible after phone screening and interested in participating will be asked if they have email access at home to receive electronic Informed Consent (see below). If so, they will be scheduled for a telephone appointment with study staff at SFVAHCS or at CAVHS for enrollment in the survey study. Eligible patients unsure about participation will be encouraged to discuss study participation with their friends and family and will be provided the option of a future follow-up recruitment call.

Study Enrollment: At the appointed date/time, eligible participants will be recontacted to be enrolled and complete the 60-minute telephone survey. At the beginning of the call, the study coordinator will email the potential participant a link to the electronic Informed Consent (IC) form to be completed via VA-approved DocuSign. DocuSign will require two factor authentication in which the potential participant enters their full name and a working email address to proceed to the IC form. In the case where a participant does not have access to email, the study coordinator will mail out a paper copy of the IC document prior to contacting the potential participant. Regardless of whether the participant receives the IC over email while on the phone or previously via mail, study staff will read through the IC document describing the purpose of the study, the study procedures and the risks and benefits of participating. After each major section of the script, the interviewer will pause and ask whether the participant understood what was read and whether they have any questions. Study staff will use clear, declarative statements when answering these questions. At the conclusion of reviewing sections of the IC, study staff will ask the subject questions to see if they understood basic aspects of the trial as well as relevant human subjects’ issues. At the script’s conclusion, if the individual is interested in participating, they will be asked to electronically sign the informed consent document or physically sign the copy that was mailed. Study staff will then be able to begin the survey over the phone immediately after obtaining the signed IC document for those using e-consent. For subjects without email/internet, the study staff will have the subject mail back their signed IC document. Once received, a telephone survey will be scheduled.

All study-related contact with potential subjects will be recorded in our SQL database. This includes the Veterans’ consent decision and, if they agree to participate, the date and version of the consent document. Potential study participants will be given as much time as they need to consider study participation, limited only by the length of the study period; those wishing to consider study participation further will be invited to call study staff back if and when they are ready to participate. In these circumstances a later date will be scheduled for completing the survey. We expect the IC process and quantitative survey will take approximately 60 minutes to complete. If the survey becomes a burden at any point during the study, participants will be given the option of completing the interview at another time.

Of note, the IC will request potential participants’ consent to possibly re-contact them after the survey to invite them to complete a qualitative interview, if they are needed for the qualitative interview (based on fulfilling purposive sampling targets) and after verifying current vaccine status (recently vaccinated against COVID-19 or persistently declining).

Once the study coordinator verifies the electronic signature on the IC form (via DocuSign) or receives a copy of the signed IC form via mail, participants are considered enrolled in the study and may complete the interviewer-administered survey (**see below for details of the survey**).

Payments to Study Subjects: We estimate that the IC and survey will take 60 minutes to complete and the qualitative interview could take up to another 60 minutes to complete. To encourage participation and to compensate Veterans for their time and effort, participants will receive compensation after completion of the survey and, if invited, after completing the qualitative interview. Veteran participants will be compensated $50 for completion of the survey and an additional $50 when the qualitative interview is complete. The participant will be paid by the VA and the VA requires that subjects receive payment via electronic funds transfer. This requires that the participant fill out their bank information (checking/savings) account number as well as social security number along with a signature on direct deposit forms to return to the VA for processing.

5.3.c. Aim 3: Implementation-Focused Interviews with VISN 16 and 21 Staff

Recruitment Methods: We are planning to conduct mid- and end-of-trial interviews with up to three high and three low vaccination rate clinics per VISN. Mid-trial change in vaccination rate will use the following calculation: 6-month vaccination rate minus baseline vaccination rate.

Because of the staggered roll-out study design, mid-trial vaccination rate changes will be calculated at 6-, 7-, and 8-months after the RCT start date. Intervention clinics will be ranked by vaccination rate change from highest to lowest and VA staff and HCPs (e.g., MD, APN, RN, LVN, Pharmacist, Psychologist, Dietician, Social Work, MSA) from these sites will be recruited for qualitative interviews. We expect to interview approximately 3-4 individuals at each site. We will interview individuals who are most familiar with the intervention and/or clinic operations. Site champions and internal and external facilitators will select these individuals to interview from the intervention sites and will provide their names to study staff, who are also VA staff members.

For the usual care sites, the point of contact for the environmental scans will provide names to study staff. The study team will email clinic staff and HCPs inviting them to participate in a 1- hour phone interview (consent 15 minutes; interview 45 minutes). If the clinic staff or HCPs agree to participate then we will set up an interview time before or after duty hours, on a break, or on leave. Informed consent for staff and HCPs will be verbal and a waiver of documentation of written informed consent will be requested from the Central IRB. Interviews will be audio recorded using a VA-compliant Audacity software.

Study Enrollment: At the appointed date/time, clinic staff and HCPs will be contacted by phone. Study staff will review the information sheet describing the purpose of the interview and research staff will obtain verbal consent to conduct and audio record the interview.

Payments to Study Subjects: We will not be reimbursing HCP and staff participants for their completion of the survey or qualitative interviews.

Inclusion/Exclusion Criteria (All Study Aims)

Each study Aim has a unique set of inclusion and exclusion criteria summarized below.

5.4. a. Aim 1: Cluster RCT of Vaccine Acceptance Intervention (Randomization at the level of VAMC)

Inclusion criteria:

o Primary care clinic visit in VISN 16 or 21

Exclusion criteria:

o Per VISN or VAMC leadership, the clinic or CBOC has extreme staffing shortages such that it would not be feasible or in the best interests of patient care to allow clinic or CBOC staff release time to participate in trainings or other meetings related to the trial.

5.4.b. Aim 2: Evaluation of recently vaccinated (N=360) and unvaccinated (N=90) Veterans across VISNs 16 and 21 at Intervention and Usual Care sites.

Inclusion criteria:

≥1 visit(s) at a participating VISN 16 or 21 inpatient or outpatient clinic after the start of the trial at their site, and

At the time of recruitment, COVID-19 vaccination status is verified as one of the following: (1) has not initiated COVID-19 vaccination; (2) has received the most current dose of the primary series of a COVID-19 vaccine after clinicians at their site received the training intervention and within the past 150 days.

Exclusion criteria:

No consistent ability to be contacted by phone

Evidence of dementia using ICD-10 diagnostic codes.

5.4.c. Aim 3: Implementation-focused Interviews with VISN 16 and 21 Staff and HCPs Inclusion criteria: Staff and HCPs work at one of the clinics involved in the study

Exclusion criteria: Staff or HCPs declines invitation to participate in the interview.

Additional Study Methods

Vaccine Acceptance Trial Overview (Aim 1). We will conduct a one-year cluster randomized parallel group trial stratified by VISN using covariate constrained randomization (e.g., baseline vaccination rates, rural/urban clinics, facility size).81 Specifically, we will compare a Usual Care (UC) versus Vaccine Acceptance Intervention (VAI) with randomization at the level of VA Medical Center (VAMC).81 In collaboration with our VA operations partners, NCP, OPCC-CT and the Office of Health Equity, VISNs 16 and 21, and the CDC Vaccine Confidence Team, we will use these conceptual frameworks-Vaccine Hesitancy and Motivation, Risk Communication, and i-PARiHS, supported by MI-informed communication, to iteratively refine, tailor, implement, facilitate and test an intervention to improve COVID-19 vaccination acceptance in Veterans across culturally and geographically diverse regions (Northern and Central California, Nevada, Hawaii, Arkansas, Louisiana, Mississippi and Texas).

5.5.a.1. Usual Care (UC) Condition: Usual Care will consist of all national and local initiatives to promote COVID-19 vaccine acceptance in Veterans such as mobile clinics, outreach calls, public service announcements targeted at Veterans, lotteries, etc. Usual Care initiatives will be documented and described in detail through quarterly environmental scans at all study sites.

##### Figure 2. Vaccine Acceptance Intervention


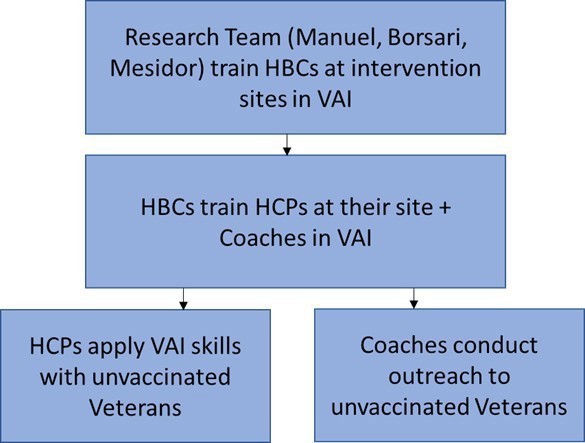


**5.5.a.2. Vaccine Acceptance Intervention (VAI):**

The VAI condition will use the evidence-informed conceptual frameworks,^19,85-88,90^ **Vaccine Hesitancy and Motivation, Risk Communication**, **Motivational Interviewing (MI)** strategies, and the **Integrated Promoting Action on Research Implementation in Health Services (i- PARiHS)** to improve COVID-19 vaccination rates among Veterans. As described in **Figure 2**, the VAI intervention utilizes a multi-pronged approach to increase Veteran vaccine acceptance. First, the research team will train Health Behavior Coordinators (HBCs) at intervention sites in VAI. HBCs will then train PACT teams at their site and Whole Health Coaches, Peer Specialists and other VA staff in VAI strategies to use with Veterans. Whole Health Coaches, Peer Specialists and other VA Staff will conduct outreach calls, using VAI strategies, with Veterans.


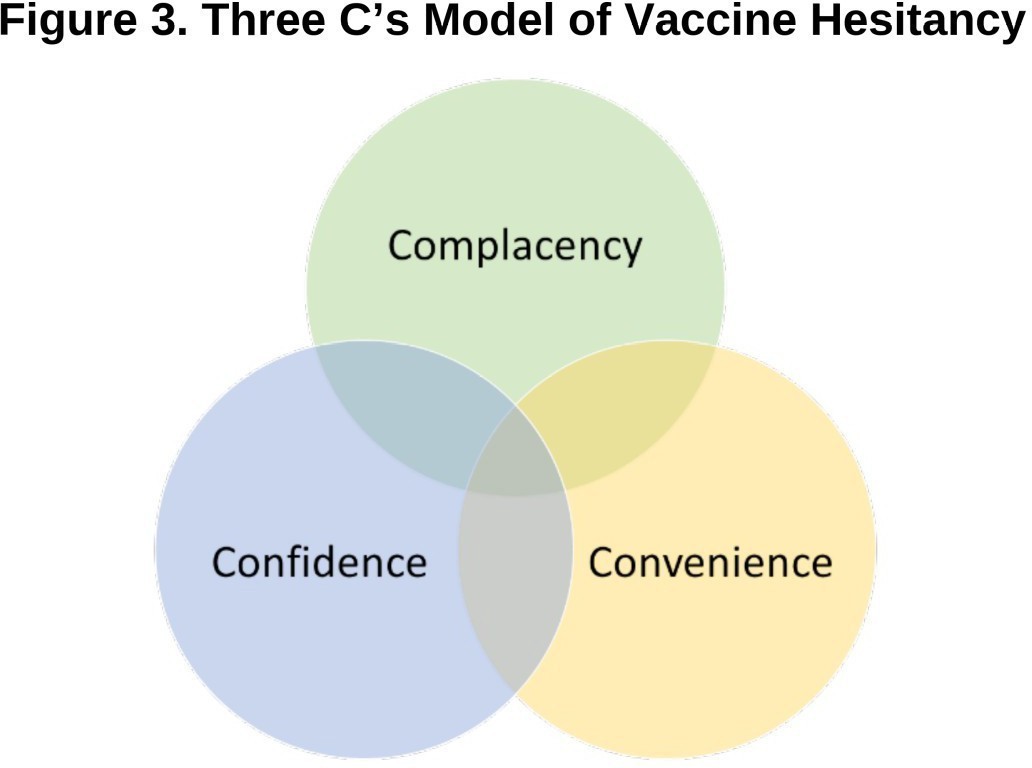


**5.5.a.2.a. Vaccine Hesitancy.** The “Vaccine Hesitancy”89 model (see Figure 3) categorizes vaccine hesitancy into 3 C’s: Complacency, not perceiving risk or need for vaccine; Convenience, lack of access or cost concerns; and Confidence, lack of trust in the effectiveness and safety of vaccines, the healthcare system, and/or the government. Confidence may be especially salient for Black Veterans and other racial/ethnic minority populations who have experienced medical exploitation and social and economic marginalization.90 The VAI is designed to shift personal vaccine hesitancy towards intrinsic acceptance for receiving the COVID-19 vaccination (see Figure 4). Vaccine hesitancy is an actionable construct because it focuses on vaccine behavior and is influenced by potentially modifiable contextual factors.^85,91^Vaccine motivation is conceived as an individual behavior influenced by what people think and feel, (i.e., confidence in vaccine benefits and safety, perceived risk to self and others, seeing negative information) and social processes (influence from others, norms, autonomy, trust in providers).^30^ Vaccine hesitancy and motivation are also dynamically influenced by ever- changing public health and vaccine policies, HCPs’ recommendations, new communications and media, all of which exist within socio-cultural, historical and political contexts, and can vary considerably for different Veteran subgroups.^85^ Notably, vaccine hesitancy may be more pronounced with COVID-19 vaccine than other vaccines due to the speed with which it was developed and authorized for emergency use, the pause on the Janssen Johnson & Johnson vaccine for a safety review,^92^ a pervasive internet and social media disinformation campaign, general distrust of the government and healthcare institutions, all compounded by a long history of racial injustice and inequities.

##### Figure 4. Vaccine Motivation Model^90^


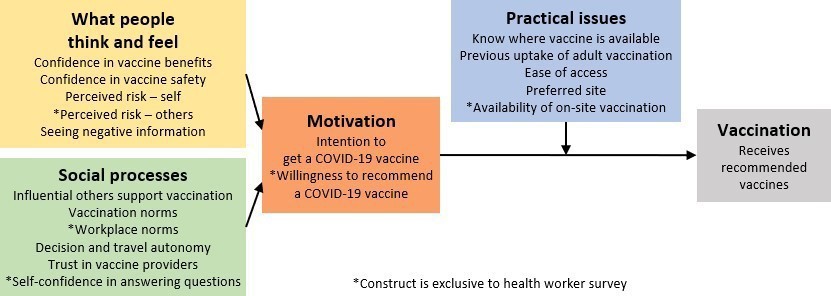


**5.5.a.2.b. Risk Communication**. In considering how to facilitate a shift from vaccine hesitancy to acceptance, a **risk communication** paradigm is a useful heuristic when there is (1) complex health or risk-related information being communicated; (2) a high level of concern; (3) expert disagreement or high uncertainty; and/or (4) low trust in those responsible for protecting against risk, which describes the context when the COVID-19 vaccine was introduced.^19^ An Institute of Medicine report described Risk Communication as a dynamic process responsive to input from several sources, changing concerns of affected populations, modification in scientific risk evidence, and newly identified needs for communication.^93^ Thus, developing a COVID-19 vaccination acceptance intervention using Risk Communication theory in the context of a pragmatic trial and implementation framework allows for input and feedback from trial stakeholders and adaptation of implementation strategies based on evolving information, and site-specific needs and preferences.^40^ Further, Risk Communication in this intervention will be guided by ethical public health practice recommendations from the CDC, the National Institutes of Health (NIH) Community Engagement Alliance (CEAL), VA Office of Health Equity, and the VA National Center for Health Promotion and Disease Prevention (NCP) and includes:^94^

- **Community engagement** - e.g., Veterans, clinics, CBOCs, VAMCs, VISNs, and VACO partners
- **Transparency** through open, honest and comprehensive education about the research and development of vaccines, monitoring of vaccine safety and efficacy, including areas of uncertainty
- Maintaining a **non-judgmental attitude** regarding skepticism and negative attitudes toward vaccines
- Selection of **trusted messengers**, e.g., HCPs and messengers from the same gender, race/ethnicity
- **Acknowledgement of indelible impacts of unethical research** and racism within healthcare that exploited and/or mistreated Black individuals and individuals from other racial/ethnic minority groups contributing to distrust of COVID-19 vaccine and the healthcare system in general^95^
- **Identifying core values for health of individuals and communities** in the face of disproportionate negative impacts of the pandemic on women with children, racial/ethnic minority groups, those with mental health disorders, the poor, and other vulnerable populations
- **Leveraging of peer influence** to promote vaccination, e.g., Veteran opinion leaders and social media and Veteran peer intervention Coaches
- **Countering misinformation** about COVID-19 vaccines in a collaborative and affirming manner

**5.5.a.2.c. Motivational Interviewing (MI).** MI is an ideal communication strategy for the VAI. MI is an empathic, non-judgmental evidence-based communication style intended to promote behavioral change consistent with the patient’s intrinsic values and goals.^87^ MI has a strong evidence-base across a variety of target behaviors in medical settings (e.g., smoking cessation, treatment engagement, dietary changes)^96^,^96,97^ and has been identified as a promising intervention to address vaccine hesitancy.^86,98-100^ There are several reasons why MI holds promise as an effective COVID-19 vaccine acceptance intervention:

1. MI is a **collaborative** approach, which is well-suited for patients who may feel they need to defend their vaccination status or intentions
2. **Open questions**, a key strategy in MI, can be used by HCPs to evoke and understand a patient’s views about vaccination.
3. **Autonomy-supportive statements** (e.g., “only you can decide whether to receive the vaccine”) are useful ways of diffusing patient resistance towards vaccination.
4. For patients who are ambivalent about vaccination, MI providers can **elicit the patients’ personal values** (e.g., to be healthy, concerns about missing work, caring for children or elderly relatives) and how receiving or not receiving the vaccine is consistent with these values.
5. Collaborative information-sharing through **Eliciting** the patient’s permission to **Provide** information and then **Eliciting** the patient’s reaction to the information (Elicit-Provide-Elicit) can be used to share COVID-19 vaccine related information that is personally relevant to the patient.
6. MI has a strong evidence-base with **culturally diverse populations**,^96^ including rural populations,^101^ and has been further adapted for use with racial and ethnic minority populations.^102-105^

##### Vaccine Acceptance Intervention (VAI) Training Methods

- - 1. **Training Health Behavior Coordinators in Vaccine Acceptance.** One of this study’s operational partners, the VA National Center for Health Promotion and Disease Prevention (NCP) supports MI training of PACT staff throughout VA through a “train-the-trainer model.” First, NCP provides in-depth training for all Health Behavior Coordinators (HBCs) that includes content on MI and other patient-centered communication skills, as well as strategies to lead an engaging MI training with PACT staff. Once HBCs have participated in the training, they are then tasked with training PACT staff at their local facility in MI and other patient-centered communication skills. HBCs have trained thousands of PACT providers across the VA health care system in the use of MI and other communication techniques.

During the first 3 months of the study, Dr. Manuel (Study mPI and SFVAHCS Health Behavior Coordinator) will collaborate with Dr. Mesidor (co-I; CAVHS Health Behavior Coordinator) and Dr. Borsari (co-I; SFVAHCS Health Behavior Coordinator) to train HBCs based at VAMCs randomized to the VAI condition in VISNs 16 and 21. HBCs will be trained in the Vaccine Acceptance Intervention, which will rely heavily on MI techniques and will also include discussions of vaccine hesitancy and risk communication. The training will closely align with NCP’s two-hour MI training for PACT staff so the structure will be familiar to HBCs but will differ from NCP’s MI training in that all content will be focused on COVID-19 vaccine acceptance. The estimated time to train HBCs will be approximately 4 hours. The training will review MI training strategies, information regarding COVID-19 vaccines, risk communication and vaccine hesitancy.

Following the training, HBCs at intervention sites will be invited to attend a twice-monthly consultation call, to discuss recent advances regarding MI and COVID-vaccine acceptance, to review training strategies and problem-solve any training or intervention issues that may arise.

- - 1. **VAI Training for PACT Staff and Coaches.** PACT providers (e.g., MDs, NPs, RNs, LVNs, MSAs and other PACT allied health professionals) will be invited to attend a two- hour training in MI, delivered by local HBCs as described above and/or by research team HBCs (i.e., Drs. Manuel, Borsari, and Mesidor). Trainings will be offered to maximize PACT team participation by offering a flexible schedule consisting of either a single two-hour training or two separate one-hour trainings. Trainings will consist of a mix of both didactic instruction and opportunities for practice, including demonstrations and role-plays. Trainings will be offered virtually and in-person via Microsoft Teams. Our partners, NCP and the CDC Vaccinate with Confidence team, have provided relevant materials that will be used to inform innovative provider training, consultation, and VAI delivery. The VAI training manual is included in the appendix.

The VAI training will include MI strategies (e.g., open-ended questions, affirmations, providing empathy), along with key aspects of vaccine hesitancy (e.g, addressing barrier to vaccination) along with risk communication strategies. Our training scripts (see appendix) informed by MI and Whole Health and risk communication strategies follows this sequence:^106^

1. Validate (provider listens with empathy, seeks to validate patients’ experience and/or beliefs), (2) Educate (provider asks permission to share information with patient without overstating benefits or minimizing harms), (3) Activate (negotiates SMART goals for vaccination; encourages questions, asks permission to share educational resources and arranges follow-up).
   - 1. **Vaccination Scripts**. PACT teams and VA staff will be introduced to MI-styled communication scripts designed specifically to increase vaccine acceptance among vaccine hesitant Veterans. These scripts were adapted by NCP, in collaboration with the CDC, to assist VA HCPs in motivating Veterans to accept flu and now COVID-19 vaccination (“Moving to COVID-19 Acceptance”). Scripts will be made into medical chart (CPRS) note templates to facilitate use with patients. Additionally, scripts will be printed on pocket-cards that will include brief communication strategies in a format that allows HCPs to access during appointments with Veterans.
     2. **Post-Training Consultation.** All intervention site PACT and VA staff will be invited to attend a weekly one-hour VISN-wide consultation session (consistent with the Office of Mental Health and Suicide Prevention’s national Motivational Interviewing training format where Drs. Manuel and Borsari serve as MI Consultants). The sessions will be led by site or research team HBCs and will be held at a time that is convenient for clinic staff. Sessions will build upon the skills taught in the initial 2-hour training. Additionally, PACT staff will be invited to share successes and challenges regarding the use of MI vaccine discussions with patients. These consultation sessions could be used as early steps to developing a learning collaborative including PACT staff and the VAI implementation team.

**5.6.e. Coach outreach to Veterans.** Whole Health Coaches and Peer Specialists will conduct outreach calls to VAI site Veterans. We will work with VISN and site leads to prioritize outreach calls but will consider prioritizing calls to Veterans with upcoming primary care appointments. Coaching sessions will utilize NCP’s “Moving to COVID- 19 Vaccine Acceptance” scripts and will be guided by the VAI framework. VAI PACT staff will also be able to place a consult to Coaches and Peer Specialists or add them as co-signers on a clinical note if they have a Veteran who would benefit from further discussion about COVID-19 vaccination.

##### Implementation Framework and Training:

- - 1. **Implementation Framework**. i-PARiHS^88^ is the implementation framework (Innovation, Recipient, Context, Facilitation) that will be used to adapt and implement the intervention.^88,107^ This framework proposes that implementation facilitation (IF) is the active ingredient, where facilitators activate and support intervention implementation by assessing and responding to the recipients of the intervention within their unique contexts.^108^ IF is a multifaceted strategy involving a process of interactive problem-solving and support that occurs in the context of a recognized need for improvement and supportive interpersonal relationships.^109^ A number of VA^107,110^ and non-VA^111,112^ studies have shown IF to be an evidence-based strategy for improving implementation of complex evidence-based interventions; this evidence is robust across diverse clinical settings, including under-resourced facilities.^113^

The i-PARiHS innovation construct refers to the process of aligning intervention evidence and characteristics with the local practice, priorities, and values. As noted above, the evidence for MI interventions includes a number of health conditions including vaccine hesitancy. The MI intervention derives from our experience developing and testing MI-informed interventions and NCP’s experience training VA staff and providers in MI methods and techniques. The recipient construct involves the people who are involved in (primary care staff and HCPs) or expected to be affected by the intervention (Veterans) and includes understanding their motivation, goals, skills, resources, leadership, and organization. The context construct includes understanding inner (local clinic) and outer (health system and community) settings related to implementation. Facilitation is the active ingredient that brings together an understanding of these constructs to enable recipients to adopt, tailor, and apply the innovation to their particular context. To inform future implementation of vaccine acceptance interventions, it will be critical to develop a deep understanding of these implementation constructs and incorporate this understanding into future vaccine acceptance intervention implementation plans. Other discrete implementation strategies that can be used within the context of IF and are relevant to this project include stakeholder discussion/engagement, use of clinician reminders about Veterans eligible for COVID vaccinations with upcoming clinic visits, train-the-trainer where local HBCs are trained to provide the motivational interviewing training to primary care HCPs and clinic staff, action planning, audit and feedback, and creating a learning collaborative for Site Champions and Internal Facilitators.^109^ The focus of Aim 3 mid-trial interviews will be on adapting the Vaccine Acceptance Intervention and implementation strategies for this study. Aim 3 end-of-trial interviews will focus on intervention and facilitation strategies to be used in a future roll-outs of vaccine acceptance interventions.

- - 1. **Implementation Facilitation Training.** One Internal Facilitator will be identified for each VAMC participating in the intervention arm of the study. Internal Facilitators or research staff who have not completed IF training or who are interested in a refresher will participate in the Implementation Facilitation training. IF is a multifaceted, implementation strategy involving a process of interactive problem-solving and support that occurs in a context of a recognized need for improvement and supportive interpersonal relationships. IF typically bundles an integrated set of activities to support uptake of effective practices, including but not limited to engaging stakeholders, identifying champions, action planning, staff training, academic detailing, problem- solving, providing technical support, audit/feedback and marketing. An increasing volume of literature supports IF as an evidence-based strategy for implementing evidence-based practices (EBPs) and other clinical innovations.

Implementation Facilitation training is supported by Department of Veterans Affairs (VA) Quality Enhancement Research Initiative (QUERI) grants QIS 18-200 and QUE 20-026. While it is targeted to implementation practitioners (clinical and operational staff and/or managers who are conducting implementation efforts), it is also often attended by VA and non-VA researchers and clinical/operational leaders charged with implementing key VA initiatives. Faculty for trainings include Dr. Kathy Dollar, Mr. Jeffrey Smith, Dr. JoAnn Kirchner, Dr. Eva Woodward, and Dr.

Jessica Martin. Together these faculty have decades of experience and substantial expertise in applying and evaluating IF strategies, providing a wealth of experiential knowledge that is shared with trainees during training. At this time, the training is only provided virtually; sessions are split into 4-hour blocks over 4 consecutive days. Internal Facilitators for this study will attend the December 7-10, 2021 training.

## **Study Evaluations for Aims 1, 2, and 3**

- - 1. **Aim 1 Evaluation Plan**. Data for Aim 1 will come from the VA Corporate Data Warehouse (CDW) and other VA databases, such as the VA COVID-19 Shared Data Resource. Outcome data will include receipt of a COVID-19 vaccination (one or more doses of Pfizer, Moderna, Johnson and Johnson, or other WHO-authorized vaccine; completed primary series with two doses of Pfizer, Moderna, one dose of Johnson and Johnson, or the specified number of doses for other WHO-authorized vaccines) and receipt of the seasonal flu vaccine (exploratory hypothesis) during the one-year trial. Covariates will include baseline demographics (e.g., age, race/ethnicity, gender, service connection), clinical and mental health diagnoses, and medications and healthcare utilization in the year prior to the start of the trial (e.g., VA and non-VA primary care visits, inpatient hospitalizations). The primary data sources for VA-administered vaccines are the ORDCOVID_Vaccine tables, which are enhancements of the CDW Immunization table and is updated weekly. Immunizations performed outside VHA for which the administration fees are reimbursed by VHA will be ascertained from CDW Community Care data in the Payment Integrity Tool (PIT) files and the Integrated Veteran Care Consolidated Data Set, which replaced PIT. Immunizations for which administration fees are reimbursed by Medicare will be ascertained from the Centers for Medicare and Medicaid Services (CMS) Institutional Outpatient, Carrier, and Medicare Advantage files using the quarterly and annual updates as they become available through the VA Information Resource Center (VIREC).

Aggregate data for county vaccination rates for adults older and younger than 65, by week, are available from the CDC COVID Data Tracker dataset “COVID-19 Vaccinations in the United States, by County”. (See **Data Analysis section** below for plan to ascertain vaccination status for Veterans who receive COVID-19 vaccination outside of VA in which vaccination data is not accessible to VA researchers.)

- - 1. **Aim 2 Evaluation Plan.** Data for Aim 2 will come from Veteran quantitative surveys and qualitative interviews. Data collection methods for Aim 2 will be telephone interview administered. We will use data from CDW to send opt-out letters to purposeful samples of newly vaccinated and unvaccinated Veterans. When telephone contact is made with potential subjects, we will introduce the study and conduct the initial telephone eligibility screen (**See Appendix Draft Phone Eligibility Script/Screener.**) We will ask the minimum number of required screening questions to gauge potential interest and study eligibility. Telephone eligibility screening questions will confirm interest in and ability to participate in a research study; vaccination status and participation in another COVID-19 research study. Study staff will determine if the potential participant is at a VAI or UC site. If the individual is interested in participating and study eligibility criteria are met, we will conduct informed consent (IC) using Docusign or mailed consents. During the IC process, we will ask the Veteran if they are willing to also participate in a separate qualitative interview.

The Veteran self-report survey developed for this study was informed by the Behavioral and Social Drivers of Vaccination (BeSD) World Health Organization model.^30^ The W.H.O model was used to identify the predictors of COVID-19 vaccine acceptance in the development of the study survey (**see draft version of the Veteran survey**). The BeSD model includes four domains: What people Think and Feel, Social Processes, Motivation, and Practical Issues. The constructs within each domain are: Thinking and Feeling: COVID experience, perceived risk, vaccine confidence, mitigation behaviors, and COVID beliefs; Practical Factors: COVID impacts, competing demands, and health problems; Social Processes: trust, social norms, discrimination, and information sources; Motivation: intention, location, motivators, and barriers. Other domains included in the draft version of the Veteran survey include seasonal flu and other vaccines (exploratory hypothesis), VA primary care experience, health status, and demographics.

The source for each question is annotated in the draft survey (see **Appendix, Draft Survey**). The following summarizes the sources for different parts of the Veteran survey. The survey items based on the BeSD model are from the CDC U.S. Vaccine Confidence Survey Question Bank, CDC COVID-19 Community Survey Question Bank, and VA QUERI Rapid Response Team COVID-19 SHEP Survey. The COVID impacts questions are from the NIH/VA/DoD Pain Management Collaboratory COVID Impacts survey. The competing demands questions are from the VA ACORN Survey. COVID-19 vaccine barriers question are from the NIH CEAL and SHEP surveys. The racial discrimination questions are from the Williams Discrimination Scale that was adapted for Veterans by LRM Hausmann et al. The trusted sources of information questions are from the Johns Hopkins COVID-19 Community Response Survey. The seasonal flu and other vaccine questions are from the San Francisco VAHCS Flu Vaccine Project and the Columbia University Collaborative Cohort of Cohorts for COVID-19 Research. The substance use questions include the 3-item AUDIT-C (Alcohol Use Disorders Identification Test) used by VHA as an alcohol use screener and PhenX Toolkit measures are used for cigarette smoking, vaping, and marijuana smoking status. The primary care experience questions are from the VA QUERI Rapid Response Team COVID-19 SHEP Survey.

Qualitative interviews will be conducted on a subset of Veterans who completed the quantitative survey according to the purposive sampling scheme described above. The qualitative interview will be audio-recorded. The audio consent will be included in the informed consent document Veterans will sign prior to completing the quantitative survey. If participants choose not to be audio-recorded, they may still participate in the quantitative survey. The consent document will have a check box in the qualitative interview section for “agree to be audio-recorded” or “do not agree to be audio-recorded.” The qualitative interview will focus on a more detailed discussion about factors contributing to their decision to get or not to get the COVID-19 vaccine (see Appendix Draft Aim 2 Qualitative Interview Guides). We will also examine Veterans’ interactions with VA HCPs and staff about COVID-19 vaccination to understand if and how the VAI impacted their vaccination decision. Other topics include previous/current barriers to getting the vaccine and ideas about how to get more Veterans vaccinated. We expect the qualitative interview will take 45-60 minutes to complete.

- - 1. **Aim 3 Evaluation Plan.** The Aim 3 qualitative interviews with HCPs and staff will occur approximately 6- and 12-months after the trial start date for each site. The Aim 3 qualitative interviews will take place by phone. The interview guide questions will be organized around the i-PARiHS constructs of Innovation/Intervention, Recipient, Context, and Facilitation. Mid- and end-of-trial qualitative data will be collected by research assistants with qualitative data experience supervised by Drs. Purcell and Elwy. The general outline for the interview guides will include an open-ended grand tour prompt (e.g., “Tell me about your experience with the Vaccine Acceptance Intervention”). Follow-up probes will address barriers and facilitators and the i-PARiHS constructs: Innovation (e.g., what worked well/not so well), Recipient (e.g., how could we get more primary care staff and providers to participate in the Vaccine Acceptance Intervention training), Context (e.g., other community, VA, VAMC, and clinic efforts to increase vaccine acceptance), and Facilitation (e.g., how could the facilitation team efforts be more effective) (see Appendix, Draft VA Staff Interview Guides). We expect the HCP and staff verbal consent process and qualitative interviews will take approximately 1 hour (consent 15 minutes; interview 45 minutes) to complete.

## **Data Analysis**

The data analysis team consists of Denise Esserman, PhD (Associate Professor of Biostatistics, Yale University and VA Connecticut Health Care System, WOC), who is experienced in conducting data analyses for multi-site randomized clinical trials in and outside of the VA healthcare system. She currently serves as the lead biostatistician on our NIH-funded wHOPE trial and prepares the open DSMB reports for this multi-site VA trial of pain management approaches for Veterans with chronic pain. Dr. Esserman will work closely with Adam Kaplan, PhD, biostatistician, and Mr. Bertenthal, our data analyst based at the SFVAHCS, who has well over a decade’s experience conducting analyses using VA EHR data and specially has experience in accessing non-VA community care data and the COVID Shared Data Resource. Both Dr. Esserman, Dr. Kaplan, and Mr. Bertenthal will be supported by our experienced data manager, Mr. Allan Chan, who built and now oversees the database application for the wHOPE trial and has a similar data application planned for this trial. Dr. Esserman and Dr. Kaplan will remain blinded throughout the course of the trial phase. Dr. Esserman will oversee the preparation of the open DSMB reports but will not prepare any closed DSMB reports or see any data by treatment arm. Mr. Bertenthal and Mr. Chan will prepare the closed DSMB reports. In addition, we will develop a detailed statistical analysis plan a priori to guard against bias should Dr. Esserman inadvertently become unblinded. The details of our data analysis plan by study aim are as follows:

***Specific Aim 1*** Conduct a one-year pragmatic cluster randomized controlled trial of a COVID- 19 Vaccine Acceptance Intervention (VAI) to determine whether, compared to Usual Care sites, Intervention sites achieve:

- ***H1a:*** A greater proportion of Veterans that receives ***one or more doses***

of COVID-19 vaccination, whether as a primary series or booster (***primary outcome***)

- ***H1b:*** A greater proportion of previously unvaccinated Veterans that completes COVID- 19 vaccination primary series.

***Exploratory hypothesis H1c:*** A greater proportion of Veterans that receives at least one COVID booster; a greater proportion of Veterans completes seasonal flu vaccination.

In addition, we will utilize the VA Corporate Data Warehouse (CDW) and other VA administrative data (e.g., VA Shared COVID-19 Data Resource), to assess other independent predictors of vaccine acceptance, including sociodemographic factors, clinical diagnoses, and health services utilization.

**Sample Size Determination:** Of the 16 VAHCS in VHA region 16 and 21, 10 VAHCS agreed to participate in the study, with an average of 9 to 10 clinics per VAHCS, and an average of at least 1,000 Veterans per clinic. These numbers were based on conservative projections of the number of Veterans at the time of randomization and account for missing vaccination (i.e., misclassification) status for Veterans who may have been vaccinated outside of the VA. We hypothesize that the larger proportion of Veterans will get vaccinated (both first and fully) within 12 months in the VAI arm compared to usual care. Calculations were conducted with PASS 19 (Kaysville, Utah) using sample size for mixed models for two sample proportions with a 3-level hierarchical design assuming an intra-cluster correlation (ICC) of 0.07 for individuals within a clinic and an ICC of 0.02 for clinics within a VAHCS.^114^ With a type I error rate of 0.05 (two-sided) we will have at least 90% power to detect between a 9.6% and 14.6% difference in vaccination rates assuming a rate in the usual care group ranging from 5% to 20% and a total sample size of 90,000 to 100,000. **Table 4** shows details of different scenarios.

##### **Table 4: Detectable difference for comparing 2 proportions in a hierarchical model assuming 5 VAHCS per arm with on average 9-10 clinics per VAHCS and, on average, 1000 individuals per CBOC/clinic, 90% power, type I error of 5% (two-sided) and level ICC of 0.07 and level 2 ICC of 0.02**

| **Total Sample Size** | **Clinics VAMC*** | **Rate in Usual Care** | **Detectable difference** |
| --- | --- | --- | --- |
| 90,000 | 9 | 5% | 9.7% |
| 100,000 | 10 | 5% | 9.6% |
| 90,000 | 9 | 10% | 12.0% |
| 100,000 | 10 | 10% | 11.8% |
| 90,000 | 9 | 15% | 13.5% |
| 100,000 | 10 | 15% | 13.3% |
| 90,000 | 9 | 20% | 14.6% |
| 100,000 | 10 | 20% | 14.4% |

*Only presented 9 and 10 clinics per VAMC for baseline rate of 5%- a negligible difference in the detectable difference between 9 and 10.

These calculations are for both the primary outcome (any dose) and secondary outcome (primary vaccination series completion). Since there is only one secondary outcome, no multiple testing procedures will be applied, and a type I error rate of 5% will also be used for this outcome.

**Analytic Plan:** All analyses will be performed using SAS v9.4 (Cary, NC) or the latest version of R (<https://cran.r-project.org/>). Statistical tests and confidence intervals will be two-sided and carried out at the 5% level of statistical significance. All analyses will follow the intention to treat principle, that is, any previously Veteran at the start of the trial who receives VA inpatient or outpatient clinic services at a participating VAMC will be considered in the denominator of the vaccination rate regardless of whether they received the intervention. Since randomization will be stratified by VISN and at the level of VAMC, but analysis will be conducted at the individual level, we will implement covariate constrained randomization for key variables (e.g., primary series vaccination rates, rural/urban distribution and facility size) and assess the adequacy of the randomization by comparing baseline characteristics between the two treatment groups.^115^ No statistical tests will be used to assess these comparisons. Variables will be summarized using descriptive statistics (means, proportions, etc.) and plots (e.g., box and whisker). Variables that appear to be different between the VAI and Usual Care sites will be adjusted for in sensitivity analyses. For analysis of the primary (one or more doses from a primary series or booster) and secondary outcome (primary series completion), we will use a multi-level model that takes into account the hierarchical clustering^116^ and adjusts for variables included in the covariate constrained randomization (e.g., rural/urban distribution and facility size) to estimate the difference in proportion of vaccinated Veterans at 12 months between VAI and Usual Care. We will adapt methods presented by Sinclair et al.^117^ to estimate the spill-over effect of the intervention (i.e., impact on the receipt of seasonal flu vaccination).

The uncertainty in vaccine records ascertainment is pertinent to discuss prior to describing how we will analyze the primary, secondary, and exploratory outcomes with standard and sensitivity analyses. There will be Veterans who receive their COVID-19 vaccinations outside of the VA system or from venues not currently captured in VA databases (i.e., State fairs, shopping malls etc.). This could inflate our denominator of unvaccinated Veterans at the start of the trial (i.e., false negatives – Veterans are actually vaccinated). It could also decrease our numerator during the trial because we could miss Veterans who received COVID-19 vaccines during the study follow-up period at non-VA sites not captured in VA data. There are two main avenues to supplement missing vaccine information in VA records; however, both have important limitations.

First, we will supplement VA vaccination records with Medicare claims data. While the federal government paid for the vaccine product, providers were permitted to bill for the cost of administering the vaccines. Based on analyses from the VA Information Resource Center (VIReC) in 2021, linking Medicare data substantially improved ascertainment. There are a few limitations. First, due to a lag, claims data will not be available for the complete study period. For Veterans enrolled in traditional Medicare fee-for-service, which account for approximately 70% of Medicare-enrolled Veterans, we anticipate data being available through 2022.^129^ For Veterans enrolled in Medicare Advantage (i.e., managed care programs), data will only be available through 2021. Second, during the early phases of vaccine rollout in 2021 when mass vaccination clinics had been common, Centers for Medicare and Medicaid Services estimated that claims were never submitted for as many as half of Medicare-age people.

Second, VHA has been developing a health information exchange with state Immunization Information Systems (IIS) in partnership with CDC to query and report Veteran immunization records. This exchange, known as the IZ Gateway, began querying state registries for 9 of the 10 study sites during July and August of 2023. Unfortunately, VHA does not plan to query state registries retrospectively for all Veterans. Instead, VHA will query individual Veterans’ records when they receive VA health services. Accordingly, we anticipate differential vaccine ascertainment in which Veterans who receive care from the time the Gateway was activated in their home state until the final data lock for this study will have near-complete vaccine ascertainment; in contrast, Veterans who do not receive care during this period will have less-complete ascertainment. Vaccine records populated through the Gateway can be identified which will help us to evaluate potential biases resulting from the timing of the Gateway rollout. For the main analyses, records populated through the IZ Gateway are being removed from the analytic dataset to avoid differential bias.

Due to limitations of the IZ Gateway and Medicare data, we will utilize one or more of several methods to ascertain the missing vaccination records and its impact on our estimates. These include comparison of our rates to those published by state and federal authorities for our VISNs or comparison of our rates to those reported by national and local VA dashboards. Alternatively, to estimate population levels of underreporting, we may need to utilize survey sampling methodology and probability weighting to randomly survey Veterans with no documentation of COVID vaccination. In sum, primary analyses for Aim 1 will utilize only VA electronic health data to ascertain Veterans’ vaccination status. We will also conduct sensitivity analyses that incorporate all available data sources (e.g., electronic health records; Medicare; state-level records; IZ Gateway).

**Sensitivity Analyses**

**Expanding Analyses to Include Vaccination Records from Medicare/Medicaid and IZ Gateway**

We plan to incorporate the vaccination records from Medicare/Medicaid and the IZ gateway and run sensitivity analyses for the primary outcome. These supportive analyses will provide variation in the estimated intervention effect related to incorporating external sources of vaccination records. On account of the IZ Gateway querying state IIS records on a prospective basis, these sensitivity analyses will focus on Veterans who have received care since the IZ Gateway was activated in their home states, which for the study sites happened during July or August of 2023. The specified statistical model is identical to that laid out in the analytic plan.

**Missing Outcomes/Misclassification of Vaccination Status**

We will conduct sensitivity analyses with a Bayesian form of multi-level logistic regression that uses the same core hierarchical model.^130^ Bayesian methods estimate parameter values (e.g., odds ratio of vaccine uptake between intervention arms) by summarizing sequentially drawn values from probability distributions with parameters updated jointly by the observed data (vaccination counts, clinic, and VAMC characteristics, etc.) and updated values of other model parameters. For our needs, the Bayesian logistic regression will incorporate a multiple imputation step that corrects the observed vaccination rates in two different ways. We can correct the total number of eligible Veterans, i.e., the denominator at each facility due to misclassifying them as unvaccinated prior to study start. Second, we can correct the count of observed vaccinations over the course of the study at each study site, i.e., the numerator, due to imperfect vaccination records ascertainment.

For the primary aim, we assume no uncertainty in Veteran eligibility since we will be using all Veterans with at least one primary care visit. This entails not subtracting a modeled probabilistic number of Veterans from the denominator of the rate of vaccination per facility, whereas the multiple imputation adds a random quantity to the count of instances of any vaccine dose (i.e., the numerator) to represent the unknown vaccinations over the course of the study that, if ignored, would be labeled as unvaccinated in the eligible sample.

Each clinic’s corrected quantity will be equal to a probabilistically modeled rate of misclassified vaccinations multiplied by the count of those Veterans labeled as unvaccinated according to the available data. The distribution for each timepoint’s rate of misclassified vaccinations will be assumed to follow a truncated Beta distribution with a lower bound estimated from available Medicare data from 2021-2022, upper bound, mode (most likely value), lower and upper percentile values determined by investigators. The Bayesian framework then updates the model’s parameters with these corrected vaccination counts instead of the observed vaccination counts. We will assess changes in the parameter estimates across a range of assumptions for the two misclassification rates and summarize the results with posterior probabilities of a non-null intervention effect, 95% credible intervals, and posterior means and medians.

**Analysis of Secondary Outcome**

We will use the same hierarchical, multi-level method for binary outcomes to analyze the secondary outcome of primary series completion in Veterans reported to not have initiated or completed the COVID-19 primary series at randomization. The model structure and adjustments are identical to that for the primary outcome. No multiple testing will be considered since there is only one secondary outcome. We will report the difference in rates between the VAI and usual care arms and the 95% confidence interval.

**Sensitivity Analyses**

**Expanding Analyses to Vaccination Records from Medicare/Medicaid and IZ Gateway**

We plan to incorporate the vaccination records from Medicare/Medicaid and the IZ gateway and run sensitivity analyses for the secondary outcome. The motivation and analysis model are identical to that laid out above. We will include Veteran records provided by these additional data sources who were not reported to have completed or initiated the primary series at randomization.

**Missing Outcomes/Misclassification of Vaccination Status**

We will use the Bayesian multiple imputation model described in the main analysis for the secondary outcome for the same motivations described therein. Due to the uncertainty in the definition of the eligibility, that is, knowing with 100% certainty that a Veteran did not complete the vaccine primary series prior to study start, the total count of eligible Veterans at each clinic (i.e., the denominator) will be reduced by a random quantity to represent the count of Veterans who were misclassified as having not completed the primary series at or before baseline. Thereafter, we will correct the count of observed primary series completions by adding a random value to represent the unknown primary series completions over the course of the study that, if ignored, would be labeled as incomplete primary series in the eligible sample. Analytical summaries will be identical to those laid out in the main analysis.

**Analysis of Exploratory Outcomes**

No control for multiplicity will be performed for exploratory outcomes. We will use the same methods proposed to analyze the primary and secondary outcomes to analyze receipt of a booster vaccination and flu vaccination. We will adapt methods presented by Sinclair et al. (2012) to estimate the spill-over effect of the intervention by analyzing receipt of seasonal flu vaccination. CDC guidelines on the minimum length of time between COVID-19 primary series completion and booster eligibility decreased during the study period. We will use the most recent guideline in effect during our study period, which was a minimum of 60 days from primary series completion to receipt of the first booster. In instances where a third mRNA vaccine dose was administered prior to 60-days following the second mRNA dose, that is classified as an additional primary series dose per CDC guidelines for Veterans with immunosuppression, and the date of that vaccination is defined as the date of primary series completion. Due to difficulties ascertaining which Veterans have immunosuppression, and the relatively loose guidelines for which conditions qualify, third doses have been defined empirically based on vaccination dates.

Because of evolving guidance on COVID vaccinations (products, dosing, etc.) in the context of fluctuating background COVID-19 prevalence at different VAHCS sites combined with seasonal flu vaccination in the Fall and Winter months, we expect temporal and site trends. Therefore, we plan to conduct the following exploratory analyses: (1) adjust for time-dependent variables; and (2) perform analyses stratified by time period and site.

***Specific Aim 2*** During an 18-month period that overlaps the trial period, we will conduct surveys in a sociodemographically and clinically diverse purposive sample of 450 Veterans equally distributed across VISNs 16 and 21 from Intervention and Usual Care sites with inpatient or outpatient clinic visits (past year) who did (N=360) and did not (N=90) receive COVID-19 vaccination. In addition, in a smaller purposive subset of Veterans (N~90), we will conduct deeper-dive qualitative interviews. These mixed methods will be used to determine correlates and emerging themes related to COVID-19 vaccine acceptance and persistent refusal.

**Sample Size Determination:** The numbers needed at each stage of recruitment to enroll a total sample of 450 Veterans over 18 months is shown in **Table 4** above. Each month we will enroll 25 Veterans; roughly 20 of whom are vaccinated and 5 of whom are unvaccinated. We will over- sample recently vaccinated Veterans to ensure an adequate sample size to be able to describe the impact of the Vaccine Acceptance Intervention on COVID-19 vaccine acceptance after considering other factors. This is primarily a descriptive aim as there is no prior data to guide sample size/power calculations. Our primary hypothesis testing is for Aim 1. For Aim 2, the main research question is whether discussions with VA HCPs and other VA staff impact Veterans’ decisions to accept COVID-19 vaccination (and secondarily flu vaccine). For similar reasons, planned sub-group analyses will be primarily descriptive. Given that we anticipate as many as 108,000 to 160,000 unvaccinated Veterans at the start of the trial across VISNs 16 and 21, if we conservatively estimate that 5% will become vaccinated each month (or 5,400-8,000), it is feasible to recruit 25 Veterans each month for a total of 450 over 18 months and ensure that our purposive sampling targets are achieved (**see Table 4 above**).

In consultation with our qualitative research team, Drs. Elwy and Purcell, co-Is, we have established a target sample size of N~90 Veterans (maintaining the purposive sampling scheme outlined above) for the in-depth qualitative interviews (following the quantitative surveys) because we anticipate being able to reach thematic saturation for the research questions posed in Aim 2 with this sample size. This subset of ~90 Veterans from the total sample of 450 Veterans, will also be identified based on purposive sociodemographic and clinical targets after the cross-sectional survey concludes.

##### **Aim 2 Analytic Plan:**

*Survey Data:* Similar to Aim 1 (above), we will use descriptive statistics (mean and standard deviations; proportions and frequencies) to describe the population. We will use generalized linear mixed models that account for clustering to model the outcomes of interest and estimate differences with 95% confidence intervals in Veteran characteristics that may be associated with the decision to receive vaccine. We will present parameter estimates (mean differences; differences in proportions; odds ratios).

*Qualitative Data from Interviews with Veterans:* A purposive clinically and sociodemographically diverse sample of roughly 90 Veterans sampled equally from VAMC Intervention and Control sites in VISNs 16 and 21, will be interviewed by telephone after they have completed the quantitative survey. As above, we will over-sample recently vaccinated Veterans. Semi- structured interview questions will address experiences with elements of the Vaccine Acceptance Intervention (i.e., discussions with HCPs about vaccination etc.) and Usual Care and what (if any) aspects of the Intervention (and Usual Care) influenced their personal decision to accept or persistently decline COVID-19 (and/or flu) vaccination. In addition, we will probe several domains contained in the survey to assess other factors influencing decisions to accept vaccination (or not). We will continue to conduct interviews with Veterans until thematic saturation within demographic and clinical subgroups is reached. Based on recommendations for qualitative sampling and accounting for subgroups of interest, we expect to conduct a total of 90 participant interviews.

Interviews will be audio-recorded using a VA-compliant Audacity software. Data from all audio- recorded interviews will be analyzed by the qualitative research team using rapid analysis methods developed for health services research.^118^**^,^**^119^ After each interview, research assistants with qualitative analysis experience will analyze recordings using rapid analysis procedures (RAP) under Drs. Purcell and Elwy oversight.^120^ RAP is a type of qualitative analysis that utilizes a coding template and is common in team-based health services research.^121^ This technique was designed to be time- and resource-efficient, balancing rigor with pragmatism and yielding results that are comparable to traditional qualitative methods.^122-124^ Rather than producing and analyzing transcripts, analysts listen to the audio-recording of each interview and prepare a written summary using a templated matrix organized by topical areas drawn from the interview guide.^120,125^ At least two trained analysts will independently listen to interviews and prepare a written summary using a templated matrix organized by topical areas. The qualitative team (analysts and Drs. Purcell and Elwy) will then meet to review and compare completed templates, identify and organize recurring themes across interviews, and refine a description of each theme. The result of this collaborative process, typically completed through review of 2-4 interviews, is a master, summary template that integrates content across individual interviews. This process of independently analyzing the same interview and meeting to discuss results to establish analytic rigor will be repeated until the analysts achieve agreement. Then, analysts will independently summarize content. Unanticipated, emergent content and observations about the interviews will be incorporated in an “Other” domain. After data from each individual interview has been summarized (i.e., templated), a summary template will be developed for vaccinated and unvaccinated Veterans. Qualitative analysts will meet with Drs. Purcell and Elwy weekly and additionally as needed. Identified themes will form the basis of qualitative summary reports. Similar analysis techniques have been used by study personnel during the implementation of other interventions.^126-128^

***Specific Aim 3*** Conduct mid-study and end-of-study qualitative interviews with VISN 16 and 21 study stakeholders, (i.e., a purposive sample of VA staff and HCPs from sites with high and low vaccination rates) to learn which aspects of the Vaccine Acceptance Intervention and implementation strategies were helpful (or not) in improving Veteran vaccine acceptance and access. Information from mid-trial process interviews will inform intervention and implementation refinements; end-of-trial interviews will inform the development of a toolkit for future vaccine acceptance intervention and implementation.

**Sample Size Determination:** We are planning to conduct mid- and end-of-trial interviews with up to three high and three low vaccination rate clinics per VISN. The number of interviews per site will be 3-4 and this will result in approximately 48 mid-trial qualitative interviews and approximately 48 end-of-trial qualitative interviews. The purposive sampling of clinics will be based on the change in vaccination rate from baseline to each endpoint.

##### **Aim 3 Analytic Plan:**

Aim 3 interview guides are organized using the i-PARIHS framework. Data collection and analysis will be similar to that described for Aim 2. The initial template for Aim 3 qualitative analysis will be based on higher-order i-PARIHS domains: facilitation, innovation, recipient, and context. The mid-study templates will be summarized at the clinic level and presented via conference call to the study team and operational partners. Qualitative results will also be presented to the facilitation team at each clinic to inform discussions about mid-study refinements. The end-of-study templates will be summarized across all sites and presented to the study team, operational partners, and facilitation teams at the VAMC level to enhance our understanding of the findings and inform future vaccine implementation and dissemination efforts.

## **Withdrawal of Subjects**

Subjects will be withdrawn if it is determined that they are actively suicidal or otherwise seriously unstable medically or psychiatrically (requiring hospitalization). This determination will be made by the mPIs, Dr. Seal (general internist), Dr. Pyne (psychiatrist) and Dr. Manuel (psychologist). In this case, withdrawal may occur without participants’ consent. We will follow the procedure outlined in the “Reporting” section of this protocol and appropriate mental health or physical health referrals will be made. Also, if at any time during the study, a participant wishes to withdraw, they may do so via phone call or written correspondence to a study staff member. We will use our database to track participants and will record if a subject withdraws, so that no future contact will be made. Participants will be made aware that there will be no negative consequences should they elect to withdraw. In particular, Veteran participants will be advised that they can continue to receive all VA health care benefits and services and VA staff will be advised that their employment will not be impacted by their decision to withdrawal.

## **Safety Assessments**

## **[Specification of Safety Parameters](#_bookmark14)**

Endorsement of mental health-related crises (e.g., suicidal or homicidal ideation) or life- threatening physical health problems during telephone assessments, and interviews

## **[Meth](#_bookmark16)****[ods and Timing for Assessing, Recording, and Analyzing](#_bookmark16)** [**Safety Parameters**](#_bookmark16)

The proposed study confers a **minimal** level of risk, posing no more risk than expected in daily life for Veterans. Veterans will be referred for mental health care, if appropriate. Participants will remain in their usual primary care, mental health and specialty care clinical treatment

relationships throughout their participation in the study. If study staff identify a concerning clinical finding, they will communicate with the participant’s other treating clinicians, as is the standard in VHA care. Based on this level of risk, the PIs will monitor and record the safety issues in accordance with IRB guidelines.

## **6.2 Data Safety Monitoring**

We will be using the HSR&D DSMB and will adhere to their reporting requirements. The Data and Safety Monitoring Board (DSMB) will review aggregate and individual participant data related to safety, data integrity, and overall conduct of the trial and will provide recommendations to continue, modify, or terminate the trial. Termination or modification may be recommended on the basis of serious safety concerns, non-compliance with human safety regulations, or serious protocol violations. The DSMB will not review interim analyses of effectiveness. This study has no pre-specified endpoints that would trigger immediate suspension of research. If termination or suspension is triggered by unexpected events (e.g., an SAE, serious noncompliance, major information security violations, loss of funding), the PIs will email notification to local site facility directors and to local site approving bodies (including local site IRB and R&D committees), along with Central IRB, within 5 business days. Additionally, the PIs will provide local sites with detailed information about procedures to ensure continuation of appropriate clinical care for research participants.

## **7.0 Reporting**

**Adverse Events.** An **adverse event (AE)** is generally defined as any unfavorable and unintended diagnosis, symptom, sign (including an abnormal laboratory finding), syndrome or disease that either occurs during the study, having been absent at baseline, or if present at baseline, appears to worsen. Adverse events are to be recorded regardless of their relationship to the study intervention. AEs that do not meet criteria for reporting within five days in accordance with VHA Handbook 1058.01 will be reported to the IRB of Record at Continuing Review. A **serious adverse event (SAE)** is generally defined as any untoward medical occurrence that results in death, is life-threatening, requires inpatient hospitalization or prolongation of existing hospitalization, results in persistent or significant disability/incapacity, or is a congenital anomaly.

PIs will be notified by the research personnel immediately of any possible event that poses a risk to a subject. In addition, at least one of the PIs (Seal, Manuel or Pyne) or all will also be notified. The PI(s) will promptly respond to the problem and mitigate any negative consequences for the participant(s) involved. The PI and/or study coordinator will report the event to the IRB of Record within the timeline as follows:

1. **Unanticipated Problems Involving Risks to Subjects or Others.** Members of the VA research community are required to ensure that unanticipated problems involving risks to participants or others in research are reported promptly to the IRB within five business days.
2. **Serious Unanticipated Problems Involving Risks to Subjects or Others.** Within five business days of becoming aware of any serious unanticipated problem involving risks to participants or others in VA research, members of the VA research community are required to ensure that the problem has been reported in writing to the IRB.
3. **Local Unanticipated SAEs**. Within five business days of becoming aware of any local (i.e., occurring in the reporting individual’s own facility) unanticipated SAE in VA research, members of the VA research community are required to ensure that the SAE has been reported in writing to the IRB.

If an AE/SAE is reported by a participant directly to the local study coordinator at their participating site, the local study coordinator will complete an AE form on the study database within 24 hours of notification. If this event also meets the criteria for an SAE, the local study coordinator will indicate this on the AE form in the study database within 24 hours. For SAEs, the local study coordinator will download a pdf of the completed SAE form and send this as an attachment via encrypted email to the Project Coordinator and PIs (Drs. Seal, Manuel, Pyne). The PIs will confer via email or phone to determine the severity and relatedness of the event to the study intervention within 48 hours of notification. The lead site will follow VA Central IRB reporting guidelines as appropriate. If we are unsure whether a document requires submission or reporting, we will contact the VA Central IRB @ the toll-free number 877-254-3130 or [VACentralIRB@va.gov](mailto:VACentralIRB@va.gov). This study will not have a Data Monitoring Committee.

- 1. **Privacy and Confidentiality**
  2. **Veteran Participants**. Our proposed research plan involves VA-enrolled Veterans receiving care in a VISN 16 or 21 primary care clinic. We will utilize VA CDW data and other VA administrative databases to examine COVID-19 and influenza vaccine acceptance and potential predictors of Veteran vaccine acceptance including sociodemographic characteristics, mental health and medical diagnoses, and other health services utilization variables among Veterans in enrolled clinics in VISN 16 and 21. We will conduct brief surveys and qualitative interviews with a purposive sample of Veterans from enrolled sites who both received and did not receive the COVID-19 vaccine. Protected health information, as described above will be collected and stored using processes described below. Study results will be reported only in aggregate. No participant names will be used in any reports or publications.
     1. **VA administrative data**. VA CDW and other VA administrative data will be accessed through the VA Informatics and Computing Infrastructure (VINCI), an initiative to improve researchers’ access to VA data and to facilitate the analysis of those data while ensuring Veterans’ privacy and data security. Domains in CDW that will be accessed include Veteran sociodemographics, ICN (Integration Control Number), health services utilization, associated ICD-10 diagnoses, COVID-19 vaccine acceptance, current and prior year influenza vaccine acceptance.
     2. **Veteran Qualitative Interviews**. Qualitative interviews will be conducted by study personnel by phone. Research personnel will work in a private location. Verbal consent will be obtained over the phone and documented in study records prior to starting the interview audio- recording and the participant will not be named after that. If a participant’s name is inadvertently recorded, it will be electronically stripped from the recording. Audio recordings will be retained in accordance with the VHA Records Control Schedule.
  3. **Stakeholder Participants**. Mid-trial and end-of-trial qualitative interviews will be conducted with VISN 16 and 21 staff with the goal of learning which aspects of the intervention and implementation were helpful, or not helpful, in improving Veteran COVID-19 vaccine acceptance. Stakeholders will be asked to consent verbally to participation. All qualitative interviews will be conducted by phone.

##### Data Security.

- - 1. **Links to Subject Identifiers**. Participant names and contact information (e.g., phone number) will be stored in a recruitment database that will be maintained throughout the duration of the study. After enrolling in the study, each survey or interview participant (including both Veterans and healthcare providers) will be assigned a unique identification number. Participant ID numbers and names will be linked in a password protected file stored on secure servers at SFVAHCS and CAVAHCS. The PIs and Project Coordinators will oversee confidentiality of this list. Research staff will have access to this database only when necessary.
    2. **Data Storage**. All study data will be stored in password protected files on secure servers, within VA-firewall protected server data drives. All data will be accessed and analyzed from encrypted PIV and password-protected computers. Current policy is that data must be archived indefinitely as described in VA Medical Center Memorandum 11-89, which is based on VA policy and the National Archives and Records Administration Records Control Schedule; however, if new policies are in effect at the end of this project, then data will be handled in accordance with those new policies.

Storage of hard copy documents:

SFVAHCS: 4150 Clement Street San Francisco, CA 94121 Building 6, Room 205,

CAVHS: 5th floor, room 5010, at 3 Financial Centre Parkway, 900 South Shackleford Rd, Little Rock AR

Storage of electronic documents:

SFVAHCS: R:\Seal\COVID vaccine trial

#### CAVHS: \\R02lithsmdc101.v16.med.va.gov\services$\HSR

S:\HSR\Pyne_J_DTC19VInter_1629257

VINCI- VA Informatics and Computing Infrastructure server

The data will be collected via RedCap, a secure VA-approved web-based tool for building surveys. A secure VA compliant database will also be used to track recruitment and enrollment. To protect against loss of confidentiality, researchers will maintain control over all research records including computer files of audio recordings of interviews. They will be coded using a numeric code and will be kept in an encrypted file behind the VA firewall and on a password protected computer in the investigator's locked office. In manuscripts, reports, publications, and other documents, the names of individual research participants will be identified only by pseudonyms, if at all. Researchers will suppress or alter distinguishing features so participants so that participants ' identities are anonymous.

- - 1. **Data Access**. Only study personnel (PIs, research staff, and others authorized by PIs) will have access to confidential study information (e.g., participant names, other identifiers). The PIs assume ultimate responsibility for data security. All study data will be stored in accordance with the Department of Veterans Affairs record control schedule (RCS 10-1). Study personnel will be fully trained in relevant ethical procedures, including participant confidentiality protections. They will undergo NIH and VA education and training in research methods and data protection procedures. The PIs will also train project staff on the latest governmental requirements on the protection of participant confidentiality and privacy.
  1. **Communication Plan**

Research personnel and study PIs (Dr. Seal, Manuel and Pyne) will communicate via encrypted email, Microsoft Teams, or through a secure VA project SharePoint site.

The SFVAHCS lead site will maintain files of all IRB documentation for VISNs 16 and 21, including consent forms and supporting documents. The research staff at SFVAHCS and VACAHCS will have a minimum of weekly telephone check-ins. At these meetings, the following will be addressed:

- - - Ensuring all required local site approvals are obtained.
    - Ensuring that the Director of any facility where research is being conducted is apprised of the study before any research efforts begin at that facility.
    - Keeping all engaged sites informed of changes to the protocol, informed consent, and HIPAA authorization.
    - Informing each site of any Serious Adverse Events, Unanticipated Problems, or interim results that may impact conduct of the study.
    - Ensuring the study is conducted according to the IRB-approved protocol.
    - Notifying local facility investigators when the study reaches the point that it no longer requires engagement of the local facility.

All points above are considered time-sensitive and will be communicated among study staff at the sites immediately. All sensitive materials will be shared among study staff at all sites via our VA server, using a VA-approved Sharepoint site, Microsoft Teams channel, or via encrypted email. Other study-related issues will also be addressed at the bi-weekly meetings (recruitment, challenges, issues and needed any changes) with the lead site and PIs to discuss and troubleshoot concerns. The PIs and co-investigators will also have quarterly telephone check- ins to discuss the progress/challenges of the study.

All study staff will be trained in the importance of maintaining confidentiality and privacy and will undergo mandatory VA education and training in research methods and data protection procedures. The PIs will also train project staff on the latest governmental requirements on the protection of participant confidentiality and privacy. In addition, we will train research study staff on all study procedures including the informed consent process.

**10.0 References**

1. Hamel L, Artiga, S., Safarpour, A., Stokes, M., & Brodie, M., . KFF COVID-19 Vaccine Monitor: COVID-19 Vaccine Access, Information, and Experiences Among Hispanic Adults in the U.S. https://[www.kff.org/coronavirus-covid-19/poll-](http://www.kff.org/coronavirus-covid-19/poll-finding/kff-covid-19-) [finding/kff](http://www.kff.org/coronavirus-covid-19/poll-finding/kff-covid-19-)- [covid-19-](http://www.kff.org/coronavirus-covid-19/poll-finding/kff-covid-19-) vaccine-monitor-access-information-experiences-hispanic- adults/. Published 2021. Accessed June 20 2021.
2. Centers for Disease Control and Prevention. COVID-19 Vaccinations in the United States. <https://covid.cdc.gov/covid-data-tracker/#vaccinations>. Published 2021. Accessed.
3. MacDonald NE. Vaccine hesitancy: Definition, scope and determinants. *Vaccine.*

2015;33(34):4161-4164.

1. Education Is Now a Bigger Factor Than Race in Desire for COVID-19 Vaccine [press release]. University of Southern California, Leonard D. Schaeffer Center for Healthy Policy and Economics. , March 2, 2021 2021.
2. Warren N, Kisely S, Siskind D. Maximizing the Uptake of a COVID-19 Vaccine in People With Severe Mental Illness: A Public Health Priority. *JAMA Psychiatry.* 2020.
3. Wang QQ, Kaelber DC, Xu R, Volkow ND. COVID-19 risk and outcomes in patients with substance use disorders: analyses from electronic health records in the United States. *Mol Psychiatry.* 2020:1-10.
4. Schmid P, Rauber D, Betsch C, Lidolt G, Denker ML. Barriers of Influenza Vaccination Intention and Behavior - A Systematic Review of Influenza Vaccine Hesitancy, 2005 - 2016. *PLoS One.* 2017;12(1):e0170550.
5. Hamel L KA, Munana C, Brodie. KFF COVID-19 Vaccine Monitor: December 2020. . Kaiser Family Foundation. . [https://www.kff.org/coronavirus-covid-19/report/kff-covid-](https://www.kff.org/coronavirus-covid-19/report/kff-covid-19-vaccine-monitor-december-2020/) [19- vaccine-monitor-december-2020/](https://www.kff.org/coronavirus-covid-19/report/kff-covid-19-vaccine-monitor-december-2020/). Published 2020. Accessed 1/15/2020.
6. Szilagyi PG, Thomas K, Shah MD, et al. National Trends in the US Public's Likelihood of Getting a COVID-19 Vaccine-April 1 to December 8, 2020. *JAMA.* 2020.
7. Corbie-Smith G, Thomas SB, St George DM. Distrust, race, and research.

*Arch Intern Med.* 2002;162(21):2458-2463.

1. Gramlich J, Funk, C. Black Americans face higher COVID-19 risks, are more hesitant to trust medical scientists, get vaccinated. Pew Research Center. [https://www.pewresearch.org/fact-tank/2020/06/04/black-americans-face-higher-covid-](https://www.pewresearch.org/fact-tank/2020/06/04/black-americans-face-higher-covid-19-risks-are-more-hesitant-to-trust-medical-scientists-get-vaccinated/) [19-risks-are-more-hesitant-to-trust-medical-scientists-get-vaccinated/](https://www.pewresearch.org/fact-tank/2020/06/04/black-americans-face-higher-covid-19-risks-are-more-hesitant-to-trust-medical-scientists-get-vaccinated/). Published 2020. Accessed 1/19, 2021.
2. Bajaj SS, Stanford FC. Beyond Tuskegee - Vaccine Distrust and Everyday Racism.

*N Engl J Med.* 2021.

1. Brandt HM, Vanderpool RC, Pilar M, Zubizarreta M, Stradtman LR. A narrative review of HPV vaccination interventions in rural U.S. communities. *Prev Med.* 2020:106407.
2. Hall LL, Xu L, Mahmud SM, Puckrein GA, Thommes EW, Chit A. A Map of Racial and Ethnic Disparities in Influenza Vaccine Uptake in the Medicare Fee-for-Service Program. *Adv Ther.* 2020;37(5):2224-2235.
3. Garcia MC RL, Bastian B, Faul, M, Dowling NF, Thomas CC, Schieb L, Hong Y, Yoon PW, Iademarco MF. . Potentially Excess Deaths from the Five Leading Causes of Death in Metropolitan and Nonmetropolitan Counties — United States, 2010–2017. *MMWR Surveill Summ 2019.*68(No. SS-10):1-11.
4. Gandhi RT, Lynch JB, Del Rio C. Mild or Moderate Covid-19. *N Engl J Med.*

2020;383(18):1757-1766.

1. James CV MR, Wilson-Frederick SM, Hall JE, Penman-Aguilar A, Bouye K. Racial/Ethnic Health Disparities Among Rural Adults — United States, 2012– 2015. *MMWR Surveill Summ.* 2017;66(23):1-9.
2. Wiley KE, Massey PD, Cooper SC, Wood N, Quinn HE, Leask J. Pregnant women's intention to take up a post-partum pertussis vaccine, and their willingness to take up the vaccine while pregnant: a cross sectional survey. *Vaccine.* 2013;31(37):3972-3978.
3. Santos SL, Helmer D, Teichman R. Risk communication in deployment-related exposure concerns. *J Occup Environ Med.* 2012;54(6):752-759.
4. Veldhoen M, Simas JP. Endemic SARS-CoV-2 will maintain post-pandemic immunity.

*Nature Reviews Immunology.* 2021;21(3):131-132.

1. Baden LR, El Sahly HM, Essink B, et al. Efficacy and Safety of the mRNA-1273 SARS- CoV-2 Vaccine. *New England Journal of Medicine.* 2020;384(5):403-416.
2. Polack FP, Thomas SJ, Kitchin N, et al. Safety and Efficacy of the BNT162b2 mRNA Covid-19 Vaccine. *New England Journal of Medicine.* 2020;383(27):2603-2615.
3. Sadoff J, Gray G, Vandebosch A, et al. Safety and Efficacy of Single-Dose Ad26.COV2.S Vaccine against Covid-19. *New England Journal of Medicine.* 2021;384(23):2187-2201.
4. Howard J. Covid-19 vaccine boosters may be necessary. Here's what you need to know. [https://www.cnn.com/2021/05/22/health/covid-19-vaccine-boosters-explainer-wellness/](https://www.cnn.com/2021/05/22/health/covid-19-vaccine-boosters-explainer-wellness/index.html) [index.html](https://www.cnn.com/2021/05/22/health/covid-19-vaccine-boosters-explainer-wellness/index.html). Published 2021 Accessed June 10, 2021.
5. Kim JH, Marks F, Clemens JD. Looking beyond COVID-19 vaccine phase 3 trials.

*Nature Medicine.* 2021;27(2):205-211.

1. Centers for Disease Control and Prevention. Interim Clinical Considerations for Use of COVID-19 Vaccines Currently Authorized in the United States. [https://www.cdc.gov/vaccines/covid-19/clinical-considerations/covid-19-vaccines-](https://www.cdc.gov/vaccines/covid-19/clinical-considerations/covid-19-vaccines-us.html?CDC_AA_refVal=https%3A%2F%2Fwww.cdc.gov%2Fvaccines%2Fcovid-19%2Finfo-by-product%2Fclinical-considerations.html&Coadministration) [us.html?CDC_AA_refVal=https%3A%2F%2Fwww.cdc.gov%2Fvaccines%2Fcovid-](https://www.cdc.gov/vaccines/covid-19/clinical-considerations/covid-19-vaccines-us.html?CDC_AA_refVal=https%3A%2F%2Fwww.cdc.gov%2Fvaccines%2Fcovid-19%2Finfo-by-product%2Fclinical-considerations.html&Coadministration) [19%2Finfo-by-product%2Fclinical-considerations.html#Coadministration](https://www.cdc.gov/vaccines/covid-19/clinical-considerations/covid-19-vaccines-us.html?CDC_AA_refVal=https%3A%2F%2Fwww.cdc.gov%2Fvaccines%2Fcovid-19%2Finfo-by-product%2Fclinical-considerations.html&Coadministration). Published 2021. Accessed.
2. Centers for Disease Control and Prevention. CDC COVID Data Tracker. <https://covid.cdc.gov/covid-data-tracker/#cases_totalcases>. Published 2021. Accessed.
3. Keating DaS, L. . The Unseen COVID-19 Risk for Unvaccinated People. .

*The Washington Post. .* May 28, 2021. , 2021;Coronavirus. .

1. Centers for Disease Control and Prevention. *Vaccines & Immunizations. Interim clinical considerations for use of mRNA COVID-19 vaccines currently authorized in the United States.* Last updated February 10, 2021. 2021.
2. Brewer NT, Chapman GB, Rothman AJ, Leask J, Kempe A. Increasing Vaccination: Putting Psychological Science Into Action. *Psychological Science in the Public Interest.* 2017;18(3):149-207.
3. Purcell N, Becker WC, Zamora KA, et al. Tailored to Fit: How an Implementation Framework Can Support Pragmatic Pain Care Trial Adaptation for Diverse Veterans Affairs Clinical Settings. *Med Care.* 2020;58 Suppl 2 9S(Suppl 2 9 Suppl):S80-s87.
4. Seal K, Becker WC, Murphy JL, Purcell N, Denneson LM, Morasco BJ et al. Whole Health Options and Pain Education (wHOPE): A Pragmatic Trial Comparing Whole Health Team versus Primary Care Group Education to Promote Non- Pharmacological Strategies to Improve Pain, Functioning and Quality of Life in Veterans--Rationale, Methods and Implementation. *Pain Medicine.* 2020;21:S91-S99.
5. Gibson CJ, Grasso J, Li Y, et al. An Integrated Pain Team Model: Impact on Pain- Related Outcomes and Opioid Misuse in Patients with Chronic Pain. *Pain Med.* 2020;21(9):1977-1984.
6. Purcell N, Zamora K, Tighe J, Li Y, Douraghi M, Seal K. The Integrated Pain Team: A Mixed-Methods Evaluation of the Impact of an Embedded Interdisciplinary Pain Care Intervention on Primary Care Team Satisfaction, Confidence, and Perceptions of Care Effectiveness. *Pain Med.* 2018;19(9):1748-1763.
7. Seal KH, Rife T, Li Y, Gibson C, Tighe J. Opioid Reduction and Risk Mitigation in VA Primary Care: Outcomes from the Integrated Pain Team Initiative. *J Gen Intern Med.* 2020;35(4):1238-1244.
8. Borsari B, Li Y, Tighe J, et al. A Pilot Trial of Collaborative Care with Motivational Interviewing to Reduce Opioid Risk and Improve Chronic Pain Management. *Addiction.* 2021.
9. Seal KH, Abadjian L, McCamish N, Shi Y, Tarasovsky G, Weingardt K. A randomized controlled trial of telephone motivational interviewing to enhance mental health treatment

engagement in Iraq and Afghanistan veterans. *Gen Hosp Psychiatry.* 2012;34(5):450- 459.

1. Seal K. Telephone Veteran Peer Coaching for Mental Health Treatment Engagement among Rural Veterans: Not What We Expected, But a Win for VA. HSRD/QUERI Conference; October 29-31, 2019, 2019; October 29-31, 2019.
2. Seal KH, Borsari B, Tighe J, et al. Optimizing pain treatment interventions (OPTI): A pilot randomized controlled trial of collaborative care to improve chronic pain management and opioid safety-Rationale, methods, and lessons learned. *Contemp Clin Trials.* 2019;77:76-85.
3. Koenig CJ, Abraham T, Zamora KA, et al. Pre-Implementation Strategies to Adapt and Implement a Veteran Peer Coaching Intervention to Improve Mental Health Treatment Engagement Among Rural Veterans. *J Rural Health.* 2016;32(4):418-428.
4. Zamora KA AT, Koenig CJ, Hill CC, Pyne JM, Seal KH. . Using an adapted case study approach to understand rural veteran experiences in patient engagement and patient- centered care research. . *Qualitative Research in Medicine and Healthcare.* 2020:54-64.
5. Seal KH, Shi Y, Cohen G, et al. Association of mental health disorders with prescription opioids and high-risk opioid use in US veterans of Iraq and Afghanistan. *Jama.* 2012;307(9):940-947.
6. Seal KH, Maguen S, Bertenthal D, et al. Observational Evidence for Buprenorphine's Impact on Posttraumatic Stress Symptoms in Veterans With Chronic Pain and Opioid Use Disorder. *J Clin Psychiatry.* 2016;77(9):1182-1188.
7. Seal KH, Bertenthal D, Barnes DE, Byers AL, Strigo I, Yaffe K. Association of Traumatic Brain Injury With Chronic Pain in Iraq and Afghanistan Veterans: Effect of Comorbid Mental Health Conditions. *Arch Phys Med Rehabil.* 2017;98(8):1636-1645.
8. Seal KH, Bertenthal D, Barnes DE, et al. Traumatic Brain Injury and Receipt of Prescription Opioid Therapy for Chronic Pain in Iraq and Afghanistan Veterans: Do Clinical Practice Guidelines Matter? *J Pain.* 2018;19(8):931-941.
9. Osilla KC, Becker K, Ecola L, et al. Study design to evaluate a group-based therapy for support persons of adults on buprenorphine/naloxone. *Addict Sci Clin Pract.* 2020;15(1):25.
10. Manuel JK, Lum PJ, Hengl NS, Sorensen JL. Smoking cessation interventions with female smokers living with HIV/AIDS: a randomized pilot study of motivational interviewing. *AIDS Care.* 2013;25(7):820-827.
11. Manuel JK, Houck JM, Moyers TB. The impact of significant others in motivational enhancement therapy: findings from project MATCH. *Behav Cogn Psychother.* 2012;40(3):297-312.
12. Moyers TB, Rowell LN, Manuel JK, Ernst D, Houck JM. The Motivational Interviewing Treatment Integrity Code (MITI 4): Rationale, Preliminary Reliability and Validity. *J Subst Abuse Treat.* 2016;65:36-42.
13. Satre DD, Manuel JK, Larios S, Steiger S, Satterfield J. Cultural Adaptation of Screening, Brief Intervention and Referral to Treatment Using Motivational Interviewing. *J Addict Med.* 2015;9(5):352-357.
14. Trick WE, Das K, Gerard MN, et al. Clinical trial of standing-orders strategies to increase the inpatient influenza vaccination rate. *Infect Control Hosp Epidemiol.* 2009;30(1):86- 88.
15. Nicosia F, Gibson, C. J., Purcell, N., Zamora, K., Tighe, J., Seal, K. H. . Women Veterans’ Experiences with Biopsychosocial Pain Care: A Qualitative Evaluation of One VA’s Integrated Pain Team. . *Pain Medicine* (in press).
16. Gibson CJ, Li Y, Bertenthal D, Huang AJ, Seal KH. Menopause symptoms and chronic pain in a national sample of midlife women veterans. *Menopause.* 2019;26(7):708-713.
17. Gibson CJ, Li Y, Jasuja GK, Self KJ, Seal KH, Byers AL. Menopausal Hormone Therapy and Suicide in a National Sample of Midlife and Older Women Veterans. *Med Care.* 2021;59:S70-s76.
18. Kelly JD, Barrie MB, Mesman AW, et al. Anatomy of a Hotspot: Chain and Seroepidemiology of Ebola Virus Transmission, Sukudu, Sierra Leone, 2015-16. *J Infect Dis.* 2018;217(8):1214-1221.
19. Kelly JD, Reid MJ, Lahiff M, Tsai AC, Weiser SD. Community-Level HIV Stigma as a Driver for HIV Transmission Risk Behaviors and Sexually Transmitted Diseases in Sierra Leone: A Population-Based Study. *J Acquir Immune Defic Syndr.* 2017;75(4):399-407.
20. Kelly JD, Weiser SD, Wilson B, et al. Ebola virus disease-related stigma among survivors declined in Liberia over an 18-month, post-outbreak period: An observational cohort study. *PLoS Negl Trop Dis.* 2019;13(2):e0007185.
21. Shelhamer MC, Wesson PD, Solari IL, et al. Prone Positioning in Moderate to Severe Acute Respiratory Distress Syndrome Due to COVID-19: A Cohort Study and Analysis of Physiology. *J Intensive Care Med.* 2021;36(2):241-252.
22. Purcell N, Zamora K, Gibson C, et al. Patient Experiences With Integrated Pain Care: A Qualitative Evaluation of One VA's Biopsychosocial Approach to Chronic Pain Treatment and Opioid Safety. *Glob Adv Health Med.* 2019;8:2164956119838845.
23. Johnson M PN. *Equity Toolkit for Patient Experience Departments.* 2017.
24. Purcell N JM. *Equity Toolkit for Core Oversight Departments.* School of Medicine, University of California, San Francisco2017.
25. D'Souza G, Springer G, Gustafson D, et al. COVID-19 symptoms and SARS-CoV-2 infection among people living with HIV in the US: the MACS/WIHS combined cohort study. *HIV Res Clin Pract.* 2020;21(5):130-139.
26. Howland M, Tennant M, Bowen DJ, et al. Psychiatrist and Psychologist Experiences with Telehealth and Remote Collaborative Care in Primary Care: A Qualitative Study. *J Rural Health.* 2020.
27. Fortney JC, Heagerty PJ, Bauer AM, et al. Study to promote innovation in rural integrated telepsychiatry (SPIRIT): Rationale and design of a randomized comparative effectiveness trial of managing complex psychiatric disorders in rural primary care clinics. *Contemp Clin Trials.* 2020;90:105873.
28. Fortney JC, Veith RC, Bauer AM, et al. Developing Telemental Health Partnerships Between State Medical Schools and Federally Qualified Health Centers: Navigating the Regulatory Landscape and Policy Recommendations. *J Rural Health.* 2019;35(3):287- 297.
29. Pyne JM, Rabalais A, Sullivan S. Mental Health Clinician and Community Clergy Collaboration to Address Moral Injury in Veterans and the Role of the Veterans Affairs Chaplain. *J Health Care Chaplain.* 2019;25(1):1-19.
30. Sullivan G, Hunt J, Haynes TF, et al. Building partnerships with rural Arkansas faith communities to promote veterans' mental health: lessons learned. *Prog Community Health Partnersh.* 2014;8(1):11-19.
31. Sullivan S, Pyne JM, Cheney AM, Hunt J, Haynes TF, Sullivan G. The pew versus the couch: relationship between mental health and faith communities and lessons learned from a VA/clergy partnership project. *J Relig Health.* 2014;53(4):1267-1282.
32. Pyne JM, Fischer EP, Mittal D, Owen R. A Patient-Centered Antipsychotic Medication Adherence Intervention: Results From a Randomized Controlled Trial. *J Nerv Ment Dis.* 2018;206(2):142-148.
33. Pyne JM, McSweeney J, Kane HS, Harvey S, Bragg L, Fischer E. Agreement between patients with schizophrenia and providers on factors of antipsychotic medication adherence. *Psychiatr Serv.* 2006;57(8):1170-1178.
34. Pyne JM, Kelly PA, Fischer EP, et al. Initial concurrent and convergent validity of the Perceived Access Inventory (PAI) for mental health services. *Psychol Serv.* 2020.
35. Bovin MJ, Miller CJ, Koenig CJ, et al. Veterans' experiences initiating VA-based mental health care. *Psychol Serv.* 2019;16(4):612-620.
36. Pyne JM, Kelly PA, Fischer EP, et al. Development of the Perceived Access Inventory: A patient-centered measure of access to mental health care. *Psychol Serv.* 2020;17(1):13- 24.
37. Connolly SL, Miller CJ, Koenig CJ, et al. Veterans' Attitudes Toward Smartphone App Use for Mental Health Care: Qualitative Study of Rurality and Age Differences. *JMIR Mhealth Uhealth.* 2018;6(8):e10748.
38. Cheney AM, Koenig CJ, Miller CJ, et al. Veteran-centered barriers to VA mental healthcare services use. *BMC Health Serv Res.* 2018;18(1):591.
39. Miller CJ, Burgess JF, Jr., Fischer EP, et al. Practical application of opt-out recruitment methods in two health services research studies. *BMC Med Res Methodol.* 2017;17(1):57.
40. Wright HM, Griffin BJ, Shoji K, et al. Pandemic-related mental health risk among front line personnel. *J Psychiatr Res.* 2020.
41. Bauer MS, Weaver K, Kim B, et al. The Collaborative Chronic Care Model for Mental Health Conditions: From Evidence Synthesis to Policy Impact to Scale-up and Spread. *Med Care.* 2019;57 Suppl 10 Suppl 3(10 Suppl 3):S221-s227.
42. Elwy AR, Bokhour BG, Maguire EM, et al. Improving healthcare systems' disclosures of large-scale adverse events: a Department of Veterans Affairs leadership, policymaker, research and stakeholder partnership. *J Gen Intern Med.* 2014;29 Suppl 4(Suppl 4):895- 903.
43. Elwy AR, Itani KM, Bokhour BG, et al. Surgeons' Disclosures of Clinical Adverse Events.

*JAMA Surg.* 2016;151(11):1015-1021.

1. Greene EJ. A SAS Macro for Covariate-Constrained Randomization of General Cluster- Randomized and Unstratified Designs. *J Stat Softw.* 2017;77(Cs1).
2. Takayama M, Wetmore CM, Mokdad AH. Characteristics associated with the uptake of influenza vaccination among adults in the United States. *Prev Med.* 2012;54(5):358-362.
3. US Department of Health and Human Services/Centers for Disease Control and Prevention/National Center for Immunization and Respiratory Diseases. *Expanding COVID-19 Vaccine Distribution to Primary Care Providers to Address Disparities in Immunization.* April 14, 2021 2021.
4. Callahan CM, Unverzagt FW, Hui SL, Perkins AJ, Hendrie HC. Six-item screener to identify cognitive impairment among potential subjects for clinical research. *Med Care.* 2002;40(9):771-781.
5. Dubé E, Vivion M, MacDonald NE. Vaccine hesitancy, vaccine refusal and the anti- vaccine movement: influence, impact and implications. *Expert Rev Vaccines.* 2015;14(1):99-117.
6. Gagneur A, Gosselin V, Dubé È. Motivational interviewing: A promising tool to address vaccine hesitancy. *Vaccine.* 2018;36(44):6553-6555.
7. Miller WR, & Rollnick, S. *Motivational Interviewing: Preparing People for Change.* 3rd edition ed. New York: Guilford Press; 2013.
8. Harvey G. KA. PARIHS revisited: from heuristic to integrated framework for the successful implementation of knowledge into practice. *Implement Sci.* 2016;11:33.
9. World Health Organization. *Addressing Vaccine Hesitancy. Secondary Addressing Vaccine Hesitancy. .* 2017.
10. Best AL FF, Kadono M, Warren RC. Institutional distrust among African Americans and building trustworthiness in the COVID-19 response: Implications for ethical public health practice. *Journal of Health Care for the Poor and Underserved* 2020
11. Cooper LZ, Larson HJ, Katz SL. Protecting public trust in immunization. *Pediatrics.*

2008;122(1):149-153.

1. FDA and CDC Lift Recommended Pause on Johnson & Johnson (Janssen) COVID-19 Vaccine Use Following Thorough Safety Review [press release]. Last updated April 23, 2021 2021.
2. Institute of Medicine. *Protecting Those Who Serve; Strategies to Protect the Health of Deployed U.S. Forces.* Washington, DC2000.
3. Centers for Disease Prevention and Control. *Crisis + Emergency Risk Communication.*

2018.

1. Robeznieks A. How to overcome COVID-19 vaccine hesitancy among Black patients.

American Medical Association

[https://www.ama-assn.org/delivering-care/public-health/how-overcome-covid-19-](https://www.ama-assn.org/delivering-care/public-health/how-overcome-covid-19-vaccine-hesitancy-among-black-patients) [vaccine-hesitancy-among-black-patients](https://www.ama-assn.org/delivering-care/public-health/how-overcome-covid-19-vaccine-hesitancy-among-black-patients). Published 2020. Accessed.

1. Hettema J, Steele J, Miller WR. Motivational interviewing. *Annu Rev Clin Psychol.*

2005;1:91-111.

1. Frost H, Campbell P, Maxwell M, et al. Effectiveness of Motivational Interviewing on adult behaviour change in health and social care settings: A systematic review of reviews. *PloS one.* 2018;13(10):e0204890-e0204890.
2. Opel DJ, Robinson JD, Spielvogle H, et al. 'Presumptively Initiating Vaccines and Optimizing Talk with Motivational Interviewing' (PIVOT with MI) trial: a protocol for a cluster randomised controlled trial of a clinician vaccine communication intervention. *BMJ Open.* 2020;10(8):e039299.
3. Reno JE TJ, Pyrzanowski J, et al. Examining strategies for improving healthcare providers' communication about adolescent HPV vaccination: evaluation of secondary outcomes in a randomized controlled trial. *Hum Vaccin Immunother* 2019;15(7):1592- 1598. .
4. McClure CC, Cataldi JR, O'Leary ST. Vaccine Hesitancy: Where We Are and Where We Are Going. *Clin Ther.* 2017;39(8):1550-1562.
5. Seal KH, Pyne JM, Manuel JK, et al. Telephone veteran peer coaching for mental health treatment engagement among rural veterans: The importance of secondary outcomes and qualitative data in a randomized controlled trial. *J Rural Health.* 2021.
6. Hanson JD, Oziel K, Sarche M, MacLehose RF, Rosenman R, Buchwald D. A culturally tailored intervention to reduce risk of alcohol-exposed pregnancies in American Indian communities: Rationale, design, and methods. *Contemporary Clinical Trials.* 2021;104:106351.
7. Rimal P, Khadka S, Bogati B, et al. Cross-cultural adaptation of motivational interviewing for use in rural Nepal. *BMC Psychol.* 2021;9(1):52.
8. Venner KL, Serier K, Sarafin R, et al. Culturally tailored evidence-based substance use disorder treatments are efficacious with an American Indian Southwest tribe: an open- label pilot-feasibility randomized controlled trial. *Addiction.* 2021;116(4):949-960.
9. Lee CS, Rosales R, Colby SM, Martin R, Cox K, Rohsenow DJ. Addressing social stressors in a brief motivational interview improve mental health symptoms for Latinx heavy drinkers. *J Clin Psychol.* 2020;76(10):1832-1850.
10. National Center for Health Promotion and Disease Prevention. *COVID-19 Vaccine Planning - How Healthy Living Teams Can Help.* Nov. 11, 2020 2020.
11. Kirchner JE, Ritchie MJ, Pitcock JA, Parker LE, Curran GM, Fortney JC. Outcomes of a partnered facilitation strategy to implement primary care-mental health. *J Gen Intern Med.* 2014;29 Suppl 4(Suppl 4):904-912.
12. Harvey G KA. Implementing evidence-based practive in healthcare: a facilitation guide. 2015.
13. Powell BJ, Waltz TJ, Chinman MJ, et al. A refined compilation of implementation strategies: results from the Expert Recommendations for Implementing Change (ERIC) project. *Implement Sci.* 2015;10:21.
14. Kilbourne AM, Almirall D, Eisenberg D, et al. Protocol: Adaptive Implementation of Effective Programs Trial (ADEPT): cluster randomized SMART trial comparing a standard versus enhanced implementation strategy to improve outcomes of a mood disorders program. *Implement Sci.* 2014;9:132.
15. Nutting PA, Miller WL, Crabtree BF, Jaen CR, Stewart EE, Stange KC. Initial lessons from the first national demonstration project on practice transformation to a patient- centered medical home. *Ann Fam Med.* 2009;7(3):254-260.
16. Wang A, Pollack T, Kadziel LA, et al. Impact of Practice Facilitation in Primary Care on Chronic Disease Care Processes and Outcomes: a Systematic Review. *J Gen Intern Med.* 2018;33(11):1968-1977.
17. Ritchie MJ, Parker LE, Edlund CN, Kirchner JE. Using implementation facilitation to foster clinical practice quality and adherence to evidence in challenged settings: a qualitative study. *BMC Health Serv Res.* 2017;17(1):294.
18. Elley CR, Kerse N, Chondros P, Robinson E. Intraclass correlation coefficients from three cluster randomised controlled trials in primary and residential health care. *Aust N Z J Public Health.* 2005;29(5):461-467.
19. Moulton LH. Covariate-based constrained randomization of group-randomized trials.

*Clin Trials.* 2004;1(3):297-305.

1. Guo G, Zhao H. Multilevel Modeling for Binary Data. *Annual Review of Sociology.*

2000;26(1):441-462.

1. Sinclair B, McConnell M, Green DP. Detecting Spillover Effects: Design and Analysis of Multilevel Experiments. *American Journal of Political Science.* 2012;56(4):1055-1069.
2. Hamilton A. Qualitative methods in rapid turn-around health services research. Health Services Research & Development Cyberseminar Web site. [http://www.hsrd.research.va.gov/for_researchers/cyber_seminars/archives/](http://www.hsrd.research.va.gov/for_researchers/cyber_seminars/archives/video_archive.cfm?SessionID=780) [video_archive.cfm?SessionID=780](http://www.hsrd.research.va.gov/for_researchers/cyber_seminars/archives/video_archive.cfm?SessionID=780). Published 2013. Accessed.
3. Palinkas LA MS, Hamilton AB,. Innovations in mixed methods evaluations.

*Annu Rev Public Health.* 2019 01:423-442.

1. Hamilton A. Qualitative methods in rapid turn-around health services research. . VA HSR&D Cyberseminar; 11 Dec. 2013, 2013.
2. Brooks J, McCluskey S, Turley E, King N. The Utility of Template Analysis in Qualitative Psychology Research. *Qual Res Psychol.* 2015;12(2):202-222.
3. Palinkas LA, Horwitz SM, Green CA, Wisdom JP, Duan N, Hoagwood K. Purposeful Sampling for Qualitative Data Collection and Analysis in Mixed Method Implementation Research. *Adm Policy Ment Health.* 2015;42(5):533-544.
4. Taylor B, Henshall C, Kenyon S, Litchfield I, Greenfield S. Can rapid approaches to qualitative analysis deliver timely, valid findings to clinical leaders? A mixed methods study comparing rapid and thematic analysis. *BMJ Open.* 2018;8(10):e019993.
5. Vindrola-Padros C, Johnson GA. Rapid Techniques in Qualitative Research: A Critical Review of the Literature. *Qual Health Res.* 2020;30(10):1596-1604.
6. Hamilton AB. Rapid qualitative analysis: Updates/developments [Internet]. . VA Health Services Research & Development Cyberseminar; 2020 Sep 29, 2020.
7. Drummond KL, Painter JT, Curran GM, et al. HIV patient and provider feedback on a telehealth collaborative care for depression intervention. *AIDS Care.* 2017.
8. Koenig CJ, Abraham T, Zamora KA, et al. Pre-implementation strategies to adapt and implement a Veteran peer coaching intervention to improve mental health treatment engagement among rural Veterans. *Journal of Rural Health.* 2016;32(4):418-428.
9. Curran GM, Pyne J, Fortney JC, et al. Development and implementation of collaborative care for depression in HIV clinics. *AIDS Care.* 2011;23(12):1626-1636.
10. Centers for Medicare and Medicaid Services. Assessing the Completeness of Medicare Claims Data for Measuring COVID-19 Vaccine Administration.; 2021. Accessed September 19, 2023
11. Polson, N. G., Scott, J. G., & Windle, J. (2013). Bayesian inference for logistic models using Pólya–Gamma latent variables. Journal of the American statistical Association, 108(504), 1339-1349.

**eFigure1. Provider and Staff Training Slides**

Slide 1

Slide 2

Slide 3

Slide 4

Slide 5

Slide 6

Slide 7

Slide 8

Slide 9

Slide 10

Slide 11

Slide 12

Slide 13

Slide 14

Slide 15

Slide 16

Slide 17

Slide 18

Slide 19

Slide 20

Slide 21

Slide 22

Slide 23

Slide 24

**eFigure2. Vaccine Communication Pocket Card**


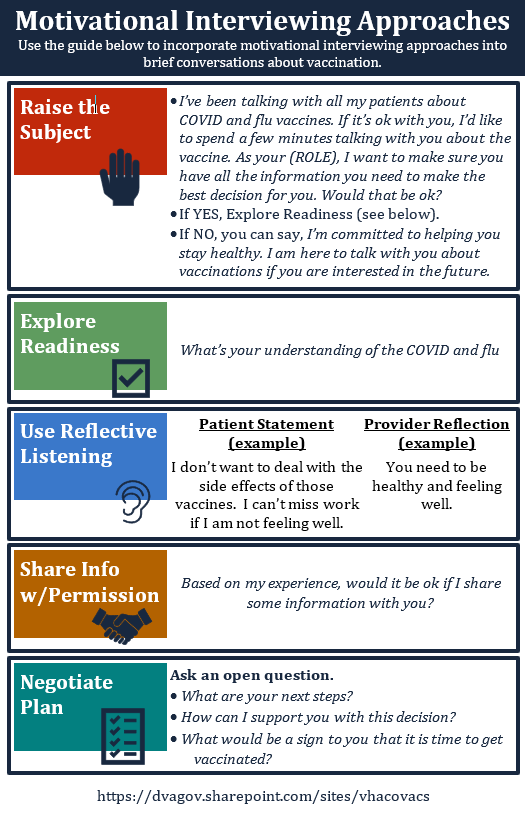


**Training Video**

**Link:** https://bcove.video/45HcHX1

**eTable 1.** **Experience of enrolled Vaccine Communication Educational Intervention**

**(VCI) vs. Usual Care (UC) VA facilities with COVID-19 vaccine access, availability,**

**and educational activities assessed at 0, 6, 12 months**

| **Environmental Scan Item** | **VCI Sites**  **N=5** | **UC Sites**  **N=5** |
| --- | --- | --- |
| There have been supply and/or access problems with the COVID-19 vaccine (> 2 time points) | 1 (20%) | 2 (40%) |
| COVID-19 vaccine has been available at the main medical center (> 2 time points) | 5 (100%) | 5 (100%) |
| COVID-19 vaccine has been available at all affiliated community-based outpatient clinics (> 2 time points) | 3 (60%) | 5 (100%) |
| There have been scheduled and drop-in COVID-19 vaccinations for patients at the main medical center (> 2 time points) | 4 (80%) | 4 (80%) |
| There have been scheduled and drop in COVID-19 vaccinations for patients at affiliated community clinics (> 2 time points) | 3 (60%) | 3 (60%) |
| COVID-19 vaccination has been accessible at the main medical center (> 2 time points)  All weekdays, regular clinic hours^1^ | 5 (100%) | 5 (100%) |
| COVID-19 vaccination has been accessible at the affiliated community clinics (> 2 time points)  All weekdays, regular clinic hours  Variable, but less than daily^3^  Less than weekly | 2 (40%)  1 (20%)  2 (40%) | 1 (20%)  4 (80%)  0 |
| There have been regular educational sessions/trainings about COVID-19 vaccination for staff (> 2 time points)^3^ | 3 (60%) | 3 (60%) |
| ^1^ One VCI facility offered extended hours or weekend vaccination in addition to weekdays during usual clinic hours.  ^2^ Multiple community clinics were associated with each main medical center facility. Vaccine accessibility varied significantly across community clinics. The majority typically offered vaccination less than daily (often on a weekly or less than weekly basis).  ^3^ These educational/training sessions were sponsored by the facilities themselves and were separate from the VCI offered as part of the trial. | | |

**eTable 2. Raw proportion of any COVID vaccinations in the year prior compared to during the**

**one-year study period in the Vaccine Communication Intervention and Usual Care Arms.**

| **Vaccination Outcome** | **Time Period** | **Overall** | **Motivational Interviewing** | **Usual Care** | **Two Proportion Difference,**  **95% CI, and P-Value** |
| --- | --- | --- | --- | --- | --- |
| **Receipt of Any Dose** | Pre-Study | 264875/338718  78.199% | 118387/148291  79.834% | 146488/190427  76.926% | 2.908%  (2.629, 3.187)  < 0.001 |
|  | Study Period | 123028/338718  36.322% | 57621/148291  38.857% | 65407/190427  34.348% | 4.509%  (4.181, 4.837)  < 0.001 |

**Supplemental Text: Sensitivity Analyses for Data Sources and Inclusion Criteria**

**Background to A Priori Sensitivity Analyses**

The uncertainty in vaccine records ascertainment was the impetus for conducting sensitivity analyses. Some veterans received COVID-19 vaccinations outside the VA system or from locations not captured in VA databases (i.e., state fairs, shopping malls etc.). This may have inflated our denominator of unvaccinated veterans at the start of the trial (i.e., false negatives, veterans are vaccinated despite appearing unvaccinated in the electronic health record). It could also decrease our numerator during the trial because we could miss veterans who received COVID-19 vaccines at non-VA sites not captured in VA data during the study follow-up period. There are two main ways to supplement missing vaccine information in VA records; however, both have important limitations. **For the primary analyses, all outcomes’ data sources are VA vaccination records augmented with Medicare claims data**.

**Sensitivity Analysis 1: Relaxing Eligibility Rule**

The target population for the intervention was veterans receiving care within the VA system. Therefore, the primary analyses constrained the sample to veterans having at least one primary care visit during the study period. **One sensitivity analysis removes this eligibility constraint to include the entire sample of veterans (with or without primary care visits during the study period).**

**Sensitivity Analysis 2: Separating VAMC Data from Medicare claims data**

While the federal government covered the cost of the vaccine product, community providers were permitted to bill for the cost of administering the COVID-19 vaccine. Based on analyses from the VA Information Resource Center (VIReC) in 2021, linking Medicare data substantially improved vaccination ascertainment with a few limitations. Due to a lag in reporting and receiving data, claims data were not available for the entire study period. For veterans enrolled in traditional Medicare fee-for-service, which accounts for approximately 70% of Medicare-enrolled veterans, data was available through 2022 [Centers for Medicare and Medicaid Services (2021)]. For veterans enrolled in Medicare Advantage (i.e., managed care programs), data was only available through 2021. Also, during the early phases of vaccine rollout in 2021, when mass vaccination clinics were common, Centers for Medicare and Medicaid Services estimated that claims were never submitted for as many as half of Medicare-age people. **One sensitivity analysis removes Medicare claims data from the VAMC data.**

**Sensitivity Analysis 3: Supplementing VAMC and Medicare claims data with IZ Gateway data**

VHA has been developing a health information exchange with state Immunization Information Systems (IIS) in partnership with CDC to query and report veteran immunization records. This exchange, known as the IZ Gateway, began querying state registries for 9 of the 10 study sites during July and August of 2023. Unfortunately, VHA does not plan to query state registries retrospectively for all veterans. Instead, VHA will query individual veterans’ records when they receive VA health services. Accordingly, we anticipate differential vaccine ascertainment in which veterans who receive care from the time the Gateway was activated in their home state until the final data lock for this study will have near-complete vaccine ascertainment; in contrast, veterans who do not receive care during this period will have less-complete ascertainment. We excluded from primary analyses due to their availability only after trial completion (July 2023) and potential for ascertainment bias among veterans with higher VA healthcare utilization. Removing IZ Gateway records resulted in an underestimation of true vaccination rates in the main analyses.

**One sensitivity analysis uses all three vaccine records sources: VAMC, Medicare claims, and IZ Gateway data.**

**Additional Sensitivity Analysis for Primary Outcome Only: Small Facilities Reassigned to Parent VAMCs**

Statistical model fitting could be an issue for *generalized linear mixed models* (GLMM) when some of the VA facilities (second level effects) have small sizes (e.g., < 100 veterans) compared to their larger counterparts. GLMMs run into poor model fitting when the number of levels in the data increase. For the proposed model, there are three levels: veteran, clinic, and parent facility. The blinded statistician analyzing the data (AK) used the R software package *lme4* with the function *glmer* and subsequently used the *allFit* function, which uses six optimization routines to fit the same model to the vaccination record data. In these fits, some optimization routines had local minimums, yielding less than optimal fits. However, all optimization routines yielded similar fixed effect estimates to the ten-thousandth place and random effect estimates (variances) to the thousandth place. To accommodate poor fitting behavior, **one sensitivity analysis reassigned the sample size and counts of any COVID-19 vaccine dose of small facilities (N < 100) to their parent facilities**.

*General Model for Sensitivity Analyses*

All of the above sensitivity analysis models follow the same fixed effects and three-level structure as the model performed for the primary analyses. We display sensitivity results for the primary and secondary outcomes in the format of adjusted (a)OR (95% confidence interval [95% CI]) and the p-values in a separate column in **eTables 3** and **5**. In addition, we show the omnibus ANOVA tests for the overall effect of a predictor on the outcome in **eTables 4** and **6**; these p-values were reported in the main manuscript. We append the results of the primary analysis for each outcome for comparison.

The supplementary **eTables 3** and **4** display these sensitivity analyses for the primary outcome, any dose. The **eTables** **5** and **6** display these sensitivity analyses for the secondary outcome, completion of primary series.

**Supplemental Text: Sensitivity Analyses Incorporating Misclassification of Vaccination Records**

We employ a Bayesian Misclassification Correction method for sensitivity analyses developed by AK. This method may be carried out on any level above the first level in the present data. We used this method on the clinic level (level 2). This implies that the inputted outcome data are vaccination status and total counts per clinic rolled up to the clinic level. In addition, individual participant-level data was rolled up to clinic level summaries. For example, instead of a participant's biological sex being a covariate adjustment, their associated clinic's proportion of the biological sexes is used. These categorical covariates may have many levels, and for each covariate, all but one of the level's proportions should be included in the analysis to avoid multicollinearity. The fitted model is identical to the primary analysis model except that the data have been rolled up to clinic level summaries; that is, all covariate adjustments are the same.

As mentioned above, there may be misclassification in the denominator and the numerator that estimate the rate of vaccination for a given clinic. We can anticipate which direction misclassification is most likely to happen, that is, the observed numerator is underestimated and the denominator is overestimated, yielding an underestimated rate of vaccination (see Equation 1 below). We call this behavior *negative misclassification*. The primary reason this occurs is that available data indicates a participant is unvaccinated but external data (not in VA data systems) reports they are vaccinated either prior to or during the study period. If we denote the true count of vaccination at a clinic *s* as $Y_{s}^{*}$ and its observed value as $Y_{s}$; the true and observed sample sizes for this clinic are $N_{s}^{*}$ and $N_{s}$, respectively. By assumption of *negative misclassification,* we have that:

$$\frac{Y_{s}^{*}}{N_{s}^{*}}>\frac{Y_{s}}{N_{s}} \left( Equation 1 \right).$$

If this misclassification is different between the intervention arms, the true estimated odds ratio could be opposite of what it is estimated to be with the observed data (e.g., the VCI actually reduced the chances of obtaining any dose compared to Usual Care, but with the observed data estimated to be equivalent or vice versa). Sensitivity analyses that account for *rates of misclassification* can provide ranges of odds ratios in favor of VCI over UC given elicited plausible ranges for these rates.

The proposed method is a combination of Bayesian logistic regression and a study-team elicited probabilistic correction in the numerator and denominator for each site. We employ the Bayesian logistic regression introduced by Polson and Scott (2013) as our base model that mirrors the standard logistic regression model, but with the addition of auxiliary parameters facilitating fully Gibbs sampling steps for the other parameters. Our addition is the data-step corrections that change the nature of the outcomes and sample size from fixed (unchanged during estimation) to *random* quantities which are discussed below.

For study clinic *s* we anticipate that the observed outcome count $Y_{s}$ is less than its true value of $Y_{s}^{*}$ assuming that some unvaccinated participants were vaccinated during the study. Also, it is plausible that the observed sample size $N_{s}$ is larger than the true value of $N_{s}^{*}$ because some participants may have met study eligibility criteria in the observed records, but unknown to the study team, they had actually met these criteria prior to the study start (e.g., an eligibility rule of being unvaccinated prior to study start). There are three ways in which we correct the observed data:

1. $N_{s}^{*}=N_{s}-\left\lceil\rho_{s,2}\left( N_{s}-Y_{s} \right) \right\rceil-\lceil\rho_{s,3}Y_{s}\rceil$
2. $E_{s}=Y_{s}-\lceil\rho_{s,3}Y_{s}\rceil$
3. $Y_{s}^{*}=E_{s}+\lceil\rho_{s,1}\left( N_{s}^{*}-E_{s} \right)\rceil$

Step (1) removes a proportion $\rho_{s,2}$ of observed unvaccinated and a proportion $\rho_{s,3}$ from the observed vaccinated during the study for being ineligible. Step (2) evaluates the corrected count of successes after ineligibility is accounted. Then Step (3) adds in a proportion $\rho_{s,1}$ of the eligible failures into the eligible successes because the observed records had them misclassified. These steps are incorporated into the Bayesian logistic regression step. The following steps describes how we assign $\boldsymbol{\rho}_{s}=\left( \rho_{s,1}, \rho_{s,2}, \rho_{s,3} \right)$ for a generic site *s*. In fact, the prior elicitation for one of the $\rho_{s}$ should be sufficient.

Prior distribution elicitation is a joint effort between the study team (including subject matter experts) and the statistician. The study team provides a most likely value (i.e., mode) for a misclassification rate $\rho_{s}$ and lower and upper bounds that would be deemed minimally plausible. To add shape to the prior distribution for $\rho_{s}$, the study team also provides a most likely range of values. The statistician interprets this as a pair of percentiles; in our usage we used the 5^th^ and 95^th^ percentiles. Our base distribution is a truncated Beta distribution with parameters $\gamma$ and $\lambda$. We optimize this distribution to provide estimates for these two parameters to construct the desired shape centered around the mode the study team provided. The second parameter $\lambda$ can be rewritten in terms of the mode of a truncated Beta distribution and the first parameter $\gamma$. Then, with the lower and upper limits and two percentiles, we optimize a truncated Beta distribution to estimate the remaining parameter $\gamma$. The optimization routine minimizes the pair of differences between the cumulative densities – an integral of the distribution from the upper percentile to the upper limit, and another integral from the lower limit to the lower percentile- against the pair of probabilities related to the percentiles (e.g., 95% - 5%). Together $\gamma$ and $\lambda$provide a specified prior distribution for $\rho_{s}$ that is sampled from in the Gibbs sampling steps. Altogether, the steps are (1) Bayesian logistic regression for estimating parameters, (2) draw a value for $\boldsymbol{\rho}_{s}$ from their elicited prior distributions, and (3) correct the observed data with $\boldsymbol{\rho}_{s}$ as stated in the correction steps above.

*Application of Bayesian Misclassification Method to Primary and Secondary Outcomes*

As described in the section *Sensitivity Analysis 3: Supplementing VAMC and Medicare claims data with IZ Gateway data*, there was an available updated source of vaccination data for some but not all veterans, based on VA medical care usage by the veteran. We took the VA with Medicare and IZ Gateway data as our gold standard to estimate rates of vaccine misclassification in the VA with Medicare claims data. *Sensitivity Analysis 3* already used data that was corrected but may have missed additional misclassified veterans due to their lack of health care use and subsequently not having their records updated with their correct vaccination status. Using all three data sources provides us with a lower bound on the rate of misclassification for the two outcomes. For the primary outcome, receipt of any dose, we are primarily concerned with misclassified vaccine labels during the study period (denote this misclassification as $\rho_{s,1}$for clinic $s)$. Eligibility misclassification is of no concern because the constraint for the primary analysis did not relate to prior vaccination status. For the secondary outcome of completing the primary series during the study period, eligibility misclassification is of greater concern since the eligibility for this outcome was to have not yet received any prior COVID-19 vaccinations before the study start (denote the eligibility misclassification rate as $\rho_{s,2}$ for clinic $s$). For completion of primary series, we assume that the rate of eligibility misclassification between participants vaccinated and unvaccinated during the study period are the same, that is $\rho_{s,2}=\rho_{s,3}$ in the above methods description.

Among the unvaccinated veterans according to the observed data, the rate of those labeled as vaccinated and queried (updated by IZ Gateway) is denoted by $q_{s,1}$. A second data point among those labeled as vaccinated in the primary data, is their rate of being unqueried and remaining labeled as unvaccinated, we denote this proportion as $q_{s,2}$. This pool of veterans we are unsure about whether their label is at all valid. However, we have prior evidence (and from the analyses conducted above) that participants who use the health care system infrequently or not at all are less likely to get vaccinated, therefore among this unqueried group chances of misclassifying their unvaccinated status are unlikely. We estimate a mode value for $\rho_{s}$, labeled $\hat{\rho}_{s}$, using this formula:

$$P\left( To Be Corrected as Vaccinated \right| Unvaccinated in Trial Data)=q_{s,1}+r\cdot q_{s,2} (Equation 2)$$

where $r$ is the fraction of the unqueried individuals in $q_{s,2}$ that would be reclassified as vaccinated. As an increasing function in $r$ the lower limit for $\hat{\rho}_{s}$ is $q_{s,1}$ and an upper bound is $q_{s,1}+q_{s,2}$, however setting $r=100\%$ is extremely unlikely (not all unqueried individuals should be reclassified as vaccinated). The sensitivity analysis for the primary outcome uses $r\in\{q_{s,1}, 25\%, 50\%\}$ represented by “Low,” “Medium,” and “High” labels. The sensitivity analysis for the secondary outcome uses $r\in\{25\%, 50\%, 60\%\}$. We computed these quantities for health care systems that contain the clinics. For example, all clinics under a health care system with the “Low” misclassification label would be assigned the associated “Low” misclassification rate of $q_{s,1}\cdot\left( 1+q_{s,2} \right)$. Importantly, for the one health care system not receiving the IZ Gateway data and another system with unusually low use of the Gateway, we specified their $\left( q_{s,1}, q_{s,2} \right)$ to be the averages of the other systems' values of these quantities.

In the following sensitivity analyses we specify the four parameters that construct the prior distribution for $\rho_{s}$. For each site, the 5^th^ and 95^th^ percentiles are equal to  $\hat{\rho}_{s}\pm0.01$ and upper and lower bounds equal to $q_{s,1}$ and $q_{s,1}+q_{s,2}$, respectively, where  $\hat{\rho}_{s}$ is the estimated mode given a value of $r$. These specifications imply that most of the plausible values for $\rho_{s}$ are tightly within $\pm1\%$ of  $\hat{\rho}_{s}$. The **eTable 7** provides the values for $q_{s,1}, q_{s,2}$ and $\hat{\rho}_{s}$ for the numerator correction of any dose. The **eTable 8** provides the values $q_{s,1}, q_{s,2}$ and $\hat{\rho}_{s}$ for the denominator correction (eligibility) for the completion of primary series.

The sensitivity analysis investigates the changes in odds ratio estimates favoring VCI over UC for increasing the chances of the two outcomes as we pair different misclassification labels between the two arms, assigning the misclassification rate estimates as modes, and perform the Bayesian Misclassification method. With three labels per arm there are a total of 9 different scenarios (e.g., VCI assigned have “Low” misclassification rates while UC has “Medium” misclassification rates).

The proposed Bayesian Misclassification method collected 30,000 posterior samples from the Gibbs sampler algorithm created by AK after rejecting 10,000 ‘burn-in’ samples, and no thinning was carried out. We report posterior averages of regression slope terms (e.g., intervention odds ratio, and adjustment effects), 95% credible intervals (95% CrI), and posterior probabilities of the odds ratio of MI over UC being greater than 1 (“Prob(OR > 1)”) and the associated non-Bayesian generalized linear mixed model (GLMM) estimate of the odds ratio (for comparison; “Prob(OR > GLMM OR)”). We use the label GLMM and GLMER interchangeably below.

*Misclassification Sensitivity Results*

*Primary Outcome: Any Dose**

The estimates were similar across all specified misclassification levels in terms of 95% credibility (**eTable 9**). These uncertainty intervals all contain the null value of 1 indicating that there is not strong evidence favoring one arm over the other. One noteworthy situation, though, is when VCI’s assumed misclassification rate is “High” while UC’s is “Low”. The odds ratio estimate in this situation is 1.162 (0.753, 1.674) with an associated posterior probability of greater than 1 being 77.1%. While this is not Bayesian significant, greater than 95% posterior probability, this signal remains strong. When assuming “Low” misclassification in VCI and “High” in UC the odd ratio estimate is 0.810 (0.490, 1.273) with an associated posterior probability of favoring MI of 18.3%. Therefore, under the assumed range of misclassification rates the effect of VCI compared to UC on vaccination uptake remains not significantly different.

*Secondary Outcome: Completion of Primary Series**

The estimates are similar across all specified misclassification levels. These uncertainty intervals all contain the null value of 1, however, all estimates are in favor of UC with most of their posterior mass below the null value of 1 (**eTable 11**). These estimates are further in agreement with the standard GLMM model that assumes no misclassification in the observed data occurred. The misclassification odds ratio estimates centered around 0.75, and all posterior probabilities favoring VCI over UC remain around 15%, indicating there is weak evidence suggesting VCI over UC. Accounting for misclassification in eligibility did not sway the estimated intervention effect to a significant degree.

*For clarity, we initially fit the full model that adjusts for the proportion of high health care utilizers per facility for the misclassification sensitivity analysis. Following this fit, we estimated that the odds ratio was around 0.7 to 0.8, VCI compared to UC. This is in contrast to the primary analyses and foregoing sensitivity analyses that evidenced no association between the intervention arm and primary series completion. To investigate, we removed several covariates one at a time from the GLMM model fit to the facility level counts and found that upon removing the adjustment of proportion of high health care utilizers, the null estimate as reported in **eTable 10** was recovered. We graphed the proportion of high utilization by facility against intervention arm labels using a box plot and observed that there was a clear divide in the distribution of this covariate. VCI facilities, overall, had lower proportions of high utilization whereas UC had higher proportions of high utilization. This unexpected multicollinearity induced a much lower than reported odds ratio of the intervention effect because the intervention effect had to compensate for the effect of the high utilization proportion. **The results reported above are the full model without adjusting for the proportion of high utilization.**

We estimated if there was any interpretative impact due to this multi-collinearity. Our hypothesis was if the predicted rates of primary series completion differed between the two models – with or without the adjustment for high utilization – on the group level then further investigation would be warranted. Using the two models separately, we predicted the rates of primary series completion on the facility level and reweighted them by facility size. Then these weighted rates were averaged between arms. The two models produced the same weighted average rates for the two arms and they matched that of the predicted rates for each arm in the individual-level analyses (results similar to **eTable 10**). In essence, while the unexpected lower OR may suggest VCI was worse for primary series completion, the predicted rates in each arm would suggest that VCI was slightly better (predicted rates of 1.21% in UC vs. 1.26% in VCI). It is the mirroring of same information between the utilization adjustment and the label of VCI vs UC that yielded, on the surface, worse results for VCI.

*Background to Post-Hoc Sensitivity Analyses*

After preliminary analyses revealed that the intervention effect was null, we carried out a sensitivity analysis similar to variable selection in observational studies where variables are removed from the adjusted model. In this case, we noticed from the *Bayesian Misclassification Sensitivity Analyses* that the utilization variable was highly collinear with the intervention effect (explained above). In these analyses we removed the categorical healthcare utilization variable from the primary analysis model. This analysis was based on data used in the primary analysis, i.e., no records added or removed based on data source (e.g., Medicare, IZ Gateway, or primary care visits during study period). We report the odds ratios, 95% CIs, and p-values for each of the effects below.

*Results:* The intervention effect was slightly attenuated across all outcomes and remained null. Other patient and clinic level features explained much more of the variability in the outcomes, still, after removal of healthcare utilization (**eTable 11**).

CITATIONS

1. Centers for Medicare and Medicaid Services. *Assessing the Completeness of Medicare Claims Data for Measuring COVID-19 Vaccine Administration*.; 2021. Accessed September 19, 2023
2. Polson, N. G., Scott, J. G., & Windle, J. (2013). Bayesian inference for logistic models using Pólya–Gamma latent variables. *Journal of the American statistical Association*, *108*(504), 1339-1349.

**eTable 3: Model Estimates from any dose sensitivity analyses (primary outcome)**

|  | **Primary Analysis** | | **No PC Restriction** | | **Small Facilities Reassigned** | | **Medicare**  **Removed** | | **IZ Gateway**  **Added** | |
| --- | --- | --- | --- | --- | --- | --- | --- | --- | --- | --- |
|  | **Odds Ratio**  **(95% CI)** | ***p*** | **Odds Ratio**  **(95% CI)** | ***p*** | **Odds Ratio**  **(95% CI)** | ***p*** | **Odds Ratio**  **(95% CI)** | ***p*** | **Odds Ratio**  **(95% CI)** | ***p*** |
| **Reference Levels** | | | | | | | | | | |
| Intercept Value | 0.06 (0.04, 0.07) | <0.001 | 0.04 (0.03, 0.05) | <0.001 | 0.06 (0.04, 0.07) | <0.001 | 0.05 (0.04, 0.06) | <0.001 | 0.07 (0.06, 0.09) | <0.001 |
| **Intervention Arm (Ref: Intervention A)** | | | | | | | | | | |
| Intervention B | 1.15 (0.89, 1.50) | 0.281 | 1.15 (0.93, 1.43) | 0.204 | 1.17 (0.89, 1.56) | 0.262 | 1.14 (0.85, 1.54) | 0.383 | 1.19 (0.91, 1.56) | 0.208 |
| **A-Priori Adjustments** | | | | | | | | | | |
| Normalized Baseline Vaccination Rate | 1.01 (0.98, 1.04) | 0.514 | 1.00 (0.98, 1.03) | 0.948 | 1.01 (0.98, 1.04) | 0.445 | 1.01 (0.98, 1.04) | 0.422 | 1.01 (0.98, 1.04) | 0.381 |
| VISN 21 (Ref: VISN 16) | 1.91 (1.42, 2.56) | <0.001 | 1.69 (1.32, 2.17) | <0.001 | 1.90 (1.40, 2.57) | <0.001 | 2.14 (1.55, 2.97) | <0.001 | 1.71 (1.27, 2.30) | <0.001 |
| **Age (Ref: <= 49)** | | | | | | | | | | |
| (49, 74] | 4.01 (3.92, 4.12) | <0.001 | 4.39 (4.30, 4.48) | <0.001 | 4.02 (3.92, 4.12) | <0.001 | 3.75 (3.66, 3.85) | <0.001 | 4.02 (3.92, 4.11) | <0.001 |
| (74, 120] | 6.39 (6.22, 6.57) | <0.001 | 6.85 (6.70, 7.01) | <0.001 | 6.39 (6.22, 6.57) | <0.001 | 5.23 (5.09, 5.38) | <0.001 | 6.58 (6.41, 6.76) | <0.001 |
| **Race (Ref: White)** | | | | | | | | | | |
| Black/AA | 1.62 (1.58, 1.65) | <0.001 | 1.53 (1.50, 1.56) | <0.001 | 1.62 (1.58, 1.65) | <0.001 | 1.69 (1.66, 1.73) | <0.001 | 1.65 (1.61, 1.68) | <0.001 |
| American Indian/Alaska Native | 0.95 (0.87, 1.03) | 0.216 | 0.92 (0.85, 0.98) | 0.016 | 0.95 (0.87, 1.03) | 0.220 | 0.95 (0.87, 1.04) | 0.252 | 0.96 (0.88, 1.04) | 0.301 |
| Asian | 1.81 (1.74, 1.89) | <0.001 | 1.82 (1.76, 1.89) | <0.001 | 1.81 (1.74, 1.89) | <0.001 | 1.76 (1.69, 1.84) | <0.001 | 1.93 (1.85, 2.01) | <0.001 |
| Native Hawaiian/Other Pacific Islander | 1.16 (1.10, 1.22) | <0.001 | 1.15 (1.10, 1.21) | <0.001 | 1.16 (1.10, 1.22) | <0.001 | 1.18 (1.12, 1.24) | <0.001 | 1.17 (1.11, 1.24) | <0.001 |
| Multiracial | 1.25 (1.18, 1.33) | <0.001 | 1.22 (1.16, 1.28) | <0.001 | 1.25 (1.18, 1.33) | <0.001 | 1.28 (1.21, 1.36) | <0.001 | 1.26 (1.19, 1.34) | <0.001 |
| Unknown Race | 1.07 (1.03, 1.11) | <0.001 | 1.03 (1.10, 1.06) | 0.022 | 1.07 (1.03, 1.11) | <0.001 | 1.07 (1.04, 1.11) | <0.001 | 1.07 (1.04, 1.11) | <0.001 |
| **Ethnicity (Ref: Not Hispanic or Latino)** | | | | | | | | | | |
| Hispanic or Latino | 1.18 (1.14, 1.22) | <0.001 | 1.16 (1.13, 1.19) | <0.001 | 1.18 (1.14, 1.22) | <0.001 | 1.21 (1.17, 1.25) | <0.001 | 1.20 (1.17, 1.24) | <0.001 |
| Declined to Answer | 0.92 (0.86, 0.98) | 0.016 | 0.94 (0.89, 1.00) | 0.047 | 0.92 (0.86, 0.98) | 0.017 | 0.91 (0.85, 0.98) | 0.012 | 0.90 (0.84, 0.97) | 0.004 |
| Unknown Ethnicity | 1.05 (1.01, 1.09) | 0.017 | 0.98 (0.95, 1.00) | 0.099 | 1.05 (1.01, 1.09) | 0.019 | 1.04 (1.00, 1.09) | 0.037 | 1.09 (1.05, 1.13) | <0.001 |
| **Sex (Ref: Female)** | | | | | | | | | | |
| Male | 0.99 (0.96, 1.02) | 0.534 | 0.94 (0.92, 0.96) | <0.001 | 0.99 (0.96, 1.02) | 0.535 | 0.97 (0.95, 1.00) | 0.045 | 0.99 (0.96, 1.01) | 0.319 |
| Unknown Sex | 0.50 (0.47, 0.55) | <0.001 | 0.40 (0.38, 0.42) | <0.001 | 0.50 (0.47, 0.54) | <0.001 | 0.49 (0.46, 0.54) | <0.001 | 0.52 (0.48, 0.56) | <0.001 |
| **HC Utilization in Year Prior to Study Period (Primary Care Visits) (Ref: [0,1])** | | | | | | | | | | |
| (1 Visit, 2 Visits] | 1.34 (1.32, 1.37) | <0.001 | 1.65 (1.62, 1.67) | <0.001 | 1.34 (1.32, 1.37) | <0.001 | 1.41 (1.38, 1.44) | <0.001 | 1.30 (1.28, 1.33) | <0.001 |
| (2 Visits, 118 Visits] | 1.74 (1.71, 1.77) | <0.001 | 2.25 (2.22, 2.29) | <0.001 | 1.74 (1.71, 1.77) | <0.001 | 1.90 (1.86, 1.93) | <0.001 | 1.62 (1.60, 1.65) | <0.001 |

**eTable 4: ANOVA Table from any dose sensitivity analyses (primary outcome, likelihood ratio test for effects: chi-square, degrees of freedom, and p-value)**

|  | **Primary Analysis** | **No PC Restriction** | **Small Facilities Reassigned** | **Medicare Removed** | **IZ Gateway Added** |
| --- | --- | --- | --- | --- | --- |
| Intervention | 1.164, DF=1 (0.281) | 1.259, DF=1 (0.262) | 1.612, DF=1 (0.204) | 1.583, DF=1 (0.208) | 0.762, DF=1 (0.383) |
| Baseline Vaccination Rate | 0.425, DF=1 (0.514) | 0.582, DF=1 (0.445) | 0.004, DF=1 (0.948) | 0.766, DF=1 (0.381) | 0.645, DF=1 (0.422) |
| VISN Code | 18.252, DF=1 (<0.001) | 17.099, DF=1 (<0.001) | 17.172, DF=1 (<0.001) | 12.362, DF=1 (<0.001) | 20.955, DF=1 (<0.001) |
| Age Categories | 17696.766, DF=2 (<0.001) | 17706.67, DF=2 (<0.001) | 28433.48, DF=2 (<0.001) | 19466.347, DF=2 (<0.001) | 14203.465, DF=2 (<0.001) |
| Race | 2484.509, DF=6 (<0.001) | 2476.664, DF=6 (<0.001) | 3052.972, DF=6 (<0.001) | 2808.148, DF=6 (<0.001) | 2743.209, DF=6 (<0.001) |
| Ethnicity | 126.207, DF=3 (<0.001) | 126.502, DF=3 (<0.001) | 147.145, DF=3 (<0.001) | 168.695, DF=3 (<0.001) | 160.554, DF=3 (<0.001) |
| Sex | 322.881, DF=2 (<0.001) | 325.708, DF=2 (<0.001) | 1589.213, DF=2 (<0.001) | 329.1, DF=2 (<0.001) | 305.911, DF=2 (<0.001) |
| Healthcare Use | 3706.253, DF=2 (<0.001) | 3709.094, DF=2 (<0.001) | 11403.433, DF=2 (<0.001) | 2931.379, DF=2 (<0.001) | 4809.199, DF=2 (<0.001) |

**eTable 5: Model estimates from completion of primary series sensitivity analyses (secondary outcome)**

|  | **Primary Analysis** | | **No PC Restriction** | | **Medicare Removed** | | **IZ Gateway Added** | |
| --- | --- | --- | --- | --- | --- | --- | --- | --- |
|  | **Odds Ratio**  **(95% CI)** | ***p*** | **Odds Ratio**  **(95% CI)** | ***p*** | **Odds Ratio**  **(95% CI)** | ***p*** | **Odds Ratio**  **(95% CI)** | ***p*** |
| **Reference Levels** | | | | | | | | |
| Intercept Value | 0.01 (0.01, 0.01) | <0.001 | 0.01 (0.01, 0.01) | <0.001 | 0.01 (0.01, 0.01) | <0.001 | 0.01 (0.01, 0.02) | <0.001 |
| **Intervention Arm (Ref: Intervention A)** | | | | | | | | |
| Intervention B | 0.94 (0.67, 1.31) | 0.706 | 0.93 (0.72, 1.21) | 0.602 | 0.90 (0.65, 1.25) | 0.533 | 0.96 (0.63, 1.46) | 0.842 |
| **A-Priori Adjustments** | | | | | | | | |
| Normalized Baseline Vaccination Rate | 1.06 (0.88, 1.27) | 0.542 | 1.00 (0.87, 1.16) | 0.995 | 1.09 (0.92, 1.31) | 0.317 | 0.99 (0.79, 1.24) | 0.926 |
| Urban (Ref: All Else) | 1.79 (1.22, 2.61) | 0.003 | 1.50 (1.12, 2.02) | 0.007 | 1.91 (1.33, 2.74) | <0.001 | 1.64 (1.03, 2.63) | 0.038 |
| **Age (Ref: <= 49)** | | | | | | | | |
| (49, 74] | 0.71 (0.61, 0.82) | <0.001 | 0.86 (0.76, 0.97) | 0.011 | 0.67 (0.58, 0.78) | <0.001 | 0.65 (0.57, 0.75) | <0.001 |
| (74, 120] | 0.76 (0.60, 0.96) | 0.020 | 1.11 (0.93, 1.32) | 0.255 | 0.50 (0.39, 0.64) | <0.001 | 0.56 (0.44, 0.71) | <0.001 |
| **Race (Ref: White)** | | | | | | | | |
| Black/AA | 1.80 (1.50, 2.15) | <0.001 | 1.77 (1.53, 2.04) | <0.001 | 1.74 (1.45, 2.09) | <0.001 | 1.91 (1.61, 2.28) | <0.001 |
| American Indian/Alaska Native | 1.10 (0.59, 2.05) | 0.775 | 1.16 (0.71, 1.90) | 0.544 | 1.10 (0.59, 2.07) | 0.756 | 1.18 (0.66, 2.09) | 0.578 |
| Asian | 2.78 (2.01, 3.84) | <0.001 | 2.22 (1.7, 2.91) | <0.001 | 2.75 (2.00, 3.80) | <0.001 | 3.19 (2.34, 4.37) | <0.001 |
| Native Hawaiian/Other Pacific Islander | 1.20 (0.75, 1.93) | 0.437 | 1.34 (0.94, 1.91) | 0.104 | 1.19 (0.75, 1.91) | 0.461 | 1.31 (0.84, 2.06) | 0.238 |
| Multiracial | 1.73 (1.13, 2.67) | 0.013 | 1.40 (0.97, 2.04) | 0.076 | 1.64 (1.06, 2.56) | 0.028 | 2.07 (1.41, 3.05) | <0.001 |
| Unknown Race | 1.38 (1.07, 1.77) | 0.012 | 1.08 (0.88, 1.34) | 0.465 | 1.34 (1.04, 1.73) | 0.023 | 1.38 (1.08, 1.75) | 0.009 |
| **Ethnicity (Ref: Not Hispanic or Latino)** | | | | | | | | |
| Hispanic or Latino | 1.17 (0.93, 1.48) | 0.182 | 1.14 (0.94, 1.40) | 0.180 | 1.15 (0.91, 1.46) | 0.251 | 1.20 (0.95, 1.50) | 0.121 |
| Declined to Answer | 0.72 (0.39, 1.35) | 0.309 | 0.93 (0.57, 1.52) | 0.770 | 0.89 (0.50, 1.58) | 0.683 | 1.18 (0.72, 1.93) | 0.502 |
| Unknown Ethnicity | 1.26 (0.96, 1.64) | 0.090 | 1.10 (0.88, 1.37) | 0.405 | 1.21 (0.92, 1.58) | 0.172 | 1.33 (1.03, 1.71) | 0.026 |
| **Sex (Ref: Female)** | | | | | | | | |
| Male | 0.85 (0.70, 1.04) | 0.111 | 0.76 (0.65, 0.89) | 0.001 | 0.83 (0.68, 1.00) | 0.052 | 0.80 (0.67, 0.96) | 0.015 |
| Unknown Sex | 0.96 (0.69, 1.32) | 0.789 | 0.56 (0.44, 0.70) | <0.001 | 1.02 (0.75, 1.40) | 0.883 | 0.98 (0.72, 1.31) | 0.868 |
| **HC Utilization in Year Prior to Study Period (Primary Care Visits) (Ref: [0,1])** | | | | | | | | |
| (1 Visit, 100 Visits] | 0.96 (0.84, 1.11) | 0.606 | 1.28 (1.14, 1.45) | <0.001 | 1.04 (0.91, 1.20) | 0.571 | 0.95 (0.84, 1.09) | 0.482 |

**eTable 6: ANOVA table from Completion of Primary Series Sensitivity Analyses (secondary outcome, likelihood ratio test for effects: chi-square, degrees of freedom, and p-value)**

|  | **Primary Analysis** | **No PC Restriction** | **Medicare Removed** | **IZ Gateway Added** |
| --- | --- | --- | --- | --- |
| Intervention | 0.143, DF = 1 (0.706) | 0.272, DF = 1 (0.602) | 0.388, DF = 1 (0.533) | 0.04, DF = 1 (0.842) |
| Baseline Vaccination Rate | 0.371, DF = 1 (0.542) | 0, DF = 1 (0.995) | 1, DF = 1 (0.317) | 0.009, DF = 1 (0.926) |
| VISN Code | 9.006, DF = 1 (0.003) | 7.271, DF = 1 (0.007) | 12.35, DF = 1 (< 0.001) | 4.309, DF = 1 (0.038) |
| Age Categories | 22.691, DF = 2 (< 0.001) | 11.175, DF = 2 (0.004) | 44.401, DF = 2 (< 0.001) | 46.031, DF = 2 (< 0.001) |
| Race | 70.926, DF = 6 (< 0.001) | 81.913, DF = 6 (< 0.001) | 65.968, DF = 6 (< 0.001) | 96.953, DF = 6 (< 0.001) |
| Ethnicity | 5.755, DF = 3 (0.124) | 2.407, DF = 3 (0.492) | 3.16, DF = 3 (0.368) | 6.24, DF = 3 (0.100) |
| Sex | 2.914, DF = 2 (0.233) | 24.342, DF = 2 (< 0.001) | 5.551, DF = 2 (0.062) | 7.393, DF = 2 (0.025) |
| Healthcare Use | 0.266, DF = 1 (0.606) | 16.853, DF = 1 (< 0.001) | 0.322, DF = 1 (0.571) | 0.494, DF = 1 (0.482) |

**eTable 7: Assumed Misclassification Rates in Primary Outcome for Health Care Systems**

| **Intervention Arm** | **Site Code** | **Misclass**  **Prop**  **(MP)** | **Unsure**  **Proportion**  **(UP)** | **Low**  **(MP + MP**  **of UP)** | **Medium**  **(MP + 25%**  **of UP)** | **High**  **(MP + 50%**  **of UP)** | **Maximum** |
| --- | --- | --- | --- | --- | --- | --- | --- |
| UC | 100 | 0.061 | 0.120 | 0.068 | 0.091 | 0.121 | 0.181 |
|  | 200 | 0.058 | 0.147 | 0.067 | 0.095 | 0.132 | 0.205 |
|  | 300 | 0.039 | 0.307 | 0.051 | 0.116 | 0.193 | 0.346 |
|  | 600 | 0.063 | 0.130 | 0.071 | 0.096 | 0.128 | 0.193 |
|  | 900 | 0.031 | 0.113 | 0.035 | 0.059 | 0.088 | 0.140 |
|  |  |  |  |  |  |  |  |
| MI | 400 | 0.064 | 0.113 | 0.071 | 0.092 | 0.121 | 0.177 |
|  | 500 | 0.064 | 0.092 | 0.070 | 0.087 | 0.110 | 0.156 |
|  | 700 | 0.074 | 0.109 | 0.082 | 0.101 | 0.129 | 0.183 |
|  | 800 | 0.058 | 0.147 | 0.067 | 0.095 | 0.132 | 0.205 |
|  | 1000 | 0.063 | 0.115 | 0.070 | 0.092 | 0.121 | 0.178 |

**eTable 8: Assumed Misclassification Rates in Eligibility of Secondary Outcome for Health Care Systems**

| **Intervention** | **Site Code** | **Misclass Prop**  **(MP)** | **Unsure Proportion (UP)** | **Low**  **(MP + 25% of UP)** | **Medium (MP + 50% of UP)** | **High**  **(MP + 66% of UP)** | **Maximum** |
| --- | --- | --- | --- | --- | --- | --- | --- |
| UC | 100 | 0.096 | 0.130 | 0.129 | 0.161 | 0.182 | 0.226 |
|  | 200 | 0.096 | 0.158 | 0.136 | 0.175 | 0.200 | 0.254 |
|  | 300 | 0.065 | 0.313 | 0.143 | 0.222 | 0.272 | 0.378 |
|  | 600 | 0.052 | 0.131 | 0.085 | 0.118 | 0.138 | 0.183 |
|  | 900 | 0.022 | 0.122 | 0.053 | 0.083 | 0.103 | 0.144 |
|  |  |  |  |  |  |  |  |
| MI | 400 | 0.148 | 0.126 | 0.180 | 0.211 | 0.232 | 0.274 |
|  | 500 | 0.133 | 0.095 | 0.157 | 0.181 | 0.196 | 0.228 |
|  | 700 | 0.088 | 0.111 | 0.116 | 0.144 | 0.161 | 0.199 |
|  | 800 | 0.096 | 0.158 | 0.136 | 0.175 | 0.200 | 0.254 |
|  | 1000 | 0.136 | 0.121 | 0.166 | 0.197 | 0.216 | 0.257 |

**eTable 9: Bayesian misclassification sensitivity results for any dose (primary outcome)**

| **Misclassification**  **Rate in UC** | **Misclassification**  **Rate in MI** | **Post Mean**  **Odds Ratio** | **Lower OR** | **Upper OR** | ***Prob(OR>1)*** | ***Prob(OR>***  ***GLMM OR)*** |
| --- | --- | --- | --- | --- | --- | --- |
| Low | Low | 1.018 | 0.638 | 1.515 | 0.496 | 0.333 |
| Low | Medium | 1.073 | 0.686 | 1.564 | 0.613 | 0.443 |
| Low | High | 1.162 | 0.753 | 1.674 | 0.771 | 0.625 |
| Medium | Low | 0.916 | 0.567 | 1.402 | 0.288 | 0.173 |
| Medium | Medium | 0.967 | 0.603 | 1.445 | 0.386 | 0.247 |
| Medium | High | 1.043 | 0.660 | 1.537 | 0.549 | 0.385 |
| High | Low | 0.810 | 0.490 | 1.273 | 0.138 | 0.082 |
| High | Medium | 0.856 | 0.526 | 1.326 | 0.191 | 0.113 |
| High | High | 0.924 | 0.569 | 1.413 | 0.302 | 0.186 |

**eTable 10: Bayesian misclassification sensitivity results for completion of primary series (secondary outcome)**

| **Misclassification**  **Rate in UC** | **Misclassification**  **Rate in MI** | **Post Mean**  **Odds Ratio** | **Lower**  **OR** | **Upper**  **OR** | ***Prob(OR>1)*** | ***Prob(OR>***  ***GLMM OR)*** |
| --- | --- | --- | --- | --- | --- | --- |
| Low | Low | 0.756 | 0.265 | 1.333 | 0.156 | 0.100 |
| Low | Medium | 0.755 | 0.276 | 1.323 | 0.155 | 0.099 |
| Low | High | 0.752 | 0.271 | 1.311 | 0.153 | 0.097 |
| Medium | Low | 0.761 | 0.275 | 1.332 | 0.160 | 0.101 |
| Medium | Medium | 0.757 | 0.272 | 1.315 | 0.157 | 0.099 |
| Medium | High | 0.759 | 0.271 | 1.313 | 0.155 | 0.096 |
| High | Low | 0.758 | 0.285 | 1.300 | 0.150 | 0.094 |
| High | Medium | 0.745 | 0.278 | 1.290 | 0.136 | 0.084 |
| High | High | 0.748 | 0.277 | 1.295 | 0.141 | 0.088 |

**eTable 11: Model Estimates after Removal of Utilization Adjustment for COVID Outcomes**

|  | **Any Dose** | | **Primary Series Completion** | | **Receipt of Booster** | |
| --- | --- | --- | --- | --- | --- | --- |
|  | **Odds Ratio**  **(95% CI)** | ***p*** | **Odds Ratio**  **(95% CI)** | ***p*** | **Odds Ratio**  **(95% CI)** | ***p*** |
| **Reference Levels** | | | | | | |
| Intercept Value | 0.07 (0.06, 0.09) | <0.001 | 0.01 (0.01, 0.01) | <0.001 | 0.13 (0.10, 0.16) | <0.001 |
| **Intervention Arm (Ref: Usual Care)** | | | | | | |
| Intervention: MI | 1.09 (0.82, 1.46) | 0.535 | 0.94 (0.67, 1.32) | 0.722 | 1.06 (0.81, 1.38) | 0.692 |
| **A-Priori Adjustments** | | | | | | |
| Normalized Baseline Vaccination Rate | 1.01 (0.98, 1.04) | 0.439 | 1.06 (0.88, 1.27) | 0.547 | 1.00 (0.98, 1.03) | 0.763 |
| VISN 21 (Ref: VISN 16) | 1.97 (1.48, 2.62) | <0.001 | 1.79 (1.23, 2.62) | 0.003 | 1.99 (1.49, 2.66) | <0.001 |
| **Age (Ref: <= 49)** | | | | | | |
| (49, 74] | 4.33 (4.23, 4.44) | <0.001 | 0.70 (0.61, 0.81) | <0.001 | 3.86 (3.75, 3.96) | <0.001 |
| (74, 120] | 6.83 (6.65, 7.02) | <0.001 | 0.76 (0.60, 0.95) | 0.017 | 5.21 (5.06, 5.37) | <0.001 |
| **Race (Ref: White)** | | | | | | |
| Black/AA | 1.63 (1.60, 1.66) | <0.001 | 1.80 (1.50, 2.15) | <0.001 | 1.47 (1.43, 1.50) | <0.001 |
| American Indian/Alaska Native | 0.95 (0.88, 1.03) | 0.246 | 1.09 (0.58, 2.05) | 0.778 | 1.03 (0.94, 1.12) | 0.554 |
| Asian | 1.80 (1.72, 1.87) | <0.001 | 2.78 (2.01, 3.85) | <0.001 | 1.54 (1.47, 1.61) | <0.001 |
| Native Hawaiian/Other Pacific Islander | 1.17 (1.11, 1.23) | <0.001 | 1.21 (0.75, 1.93) | 0.435 | 1.09 (1.03, 1.15) | 0.004 |
| Multiracial | 1.26 (1.19, 1.34) | <0.001 | 1.73 (1.12, 2.67) | 0.013 | 1.22 (1.15, 1.31) | <0.001 |
| Unknown Race | 1.05 (1.02, 1.09) | 0.004 | 1.38 (1.07, 1.77) | 0.011 | 1.04 (1.00, 1.08) | 0.057 |
| **Ethnicity (Ref: Not Hispanic or Latino)** | | | | | | |
| Hispanic or Latino | 1.19 (1.15, 1.22) | <0.001 | 1.17 (0.93, 1.49) | 0.182 | 1.13 (1.10, 1.17) | <0.001 |
| Ethnicity: Declined to Answer | 0.93 (0.87, 1.00) | 0.045 | 0.72 (0.39, 1.35) | 0.307 | 0.94 (0.87, 1.01) | 0.099 |
| Unknown Ethnicity | 1.03 (0.99, 1.07) | 0.141 | 1.26 (0.96, 1.64) | 0.093 | 0.97 (0.93, 1.01) | 0.169 |
| **Sex (Ref: Female)** | | | | | | |
| Male | 0.95 (0.93, 0.98) | <0.001 | 0.86 (0.71, 1.04) | 0.119 | 0.95 (0.92, 0.98) | 0.001 |
| Unknown Sex | 0.41 (0.38, 0.44) | <0.001 | 0.97 (0.70, 1.33) | 0.842 | 0.66 (0.60, 0.72) | <0.001 |
